# Highly diverse flavobacterial phages isolated from North Sea spring blooms

**DOI:** 10.1038/s41396-021-01097-4

**Published:** 2021-09-02

**Authors:** Nina Bartlau, Antje Wichels, Georg Krohne, Evelien M. Adriaenssens, Anneke Heins, Bernhard M. Fuchs, Rudolf Amann, Cristina Moraru

**Affiliations:** 1 Max Planck Institute for Marine Microbiology, Bremen, Germany; 2 Alfred Wegener Institute Helmholtz Center for Polar and Marine Research, Biologische Anstalt Helgoland, Heligoland, Germany; 3 Imaging Core Facility, Biocenter, University of Würzburg, Würzburg, Germany; 4 Quadram Institute Bioscience, Norwich Research Park, Norwich, UK; 5 Institute for Chemistry and Biology of the Marine Environment, University of Oldenburg, Oldenburg, Germany

**Keywords:** Bacteriophages, Water microbiology

## Abstract

It is generally recognized that phages are a mortality factor for their bacterial hosts. This could be particularly true in spring phytoplankton blooms, which are known to be closely followed by a highly specialized bacterial community. We hypothesized that phages modulate these dense heterotrophic bacteria successions following phytoplankton blooms. In this study, we focused on *Flavobacteriia*, because they are main responders during these blooms and have an important role in the degradation of polysaccharides. A cultivation-based approach was used, obtaining 44 lytic flavobacterial phages (flavophages), representing twelve new species from two viral realms. Taxonomic analysis allowed us to delineate ten new phage genera and ten new families, from which nine and four, respectively, had no previously cultivated representatives. Genomic analysis predicted various life styles and genomic replication strategies. A likely eukaryote-associated host habitat was reflected in the gene content of some of the flavophages. Detection in cellular metagenomes and by direct-plating showed that part of these phages were actively replicating in the environment during the 2018 spring bloom. Furthermore, CRISPR/Cas spacers and re-isolation during two consecutive years suggested that, at least part of the new flavophages are stable components of the microbial community in the North Sea. Together, our results indicate that these diverse flavophages have the potential to modulate their respective host populations.

## Introduction

Marine bacteriophages outnumber their hosts by one order of magnitude in surface seawater and infect 10–45% of the bacterial cells at any given time [1–3]. They have a major impact on bacterioplankton dynamics. This impact can be density dependent [4] and take many forms. By lysing infected cells, viruses decrease the abundance of their host population, shifting the dominant bacterial population, and recycling the intracellular nutrients inside the same trophic level [5]. By expressing auxiliary metabolic genes, phages likely enhance the metabolic capabilities of the virocells [6, 7]. By transferring pieces of host DNA, they can drive bacterial evolution [8]. By blocking superinfections with other phages [9], they can protect from immediate lysis. Potentially phages even influence carbon export to the deep ocean due to aggregation of cell debris resulted from cell lysis, called the viral shuttle [10, 11]. Marine phages modulate not only their hosts, but also the diversity and function of whole ecosystems. This global impact is reflected in a high phage abundance [12] and diversity [13, 14].

Phages are also well known for modulating bacterial communities in temperate coastal oceans. Here, the increase in temperature and solar radiation in spring induces the formation of phytoplankton blooms, which are often dominated by diatoms [15], and are globally important components of the marine carbon cycle. These ephemeral events release high amounts of organic matter, which fuels subsequent blooms of heterotrophic bacteria. *Flavobacteriia* belong to the main responders [16, 17] and their increase is linked to the release of phytoplankton derived polysaccharides [18, 19]. These polysaccharides are produced by microalgae as storage compounds, cell wall building blocks, and exudates [20–22]. This highly complex organic matter is likely converted by the *Flavobacteriia* to low molecular weight compounds and thus they are important for the carbon turnover during phytoplankton blooms [18, 23–25]. Recurrent genera like *Polaribacter, Maribacter*, and *Tenacibaculum* succeed each other in a highly dynamic fashion [19]. This bacterial succession is likely triggered by the availability and quality of substrates such as polysaccharides [18, 19], yet it cannot be fully understood without considering mortality factors such as grazing by protists, and viral lysis. Grazing by protists is mostly size dependent [26], whereas viral lysis is highly host specific [27].

Based on the availability of suitable host bacteria, marine phages can be obtained with standard techniques. Over the years notable numbers of phages infecting marine *Alphaproteobacteria* (e.g., [28, 29]), *Gammproteobacteria* (e.g., [30]), and *Cyanobacteria* (e.g., [31–36]) have been isolated. Despite the importance of *Flavobacteriia* as primary degraders of high molecular weight algal derived matter only few marine flavobacterial phages, to which we refer in the following as flavophages, have been characterized. This includes several *Cellulophaga* phage isolates from the Baltic Sea, covering all phage morphotypes in the realm *Duplodnaviria* and also two different phage groups in the realm *Monodnaviria* [27, 37]. Phages were also isolated for members of the genera *Polaribacter* [38], *Flavobacterium* [39, 40], *Croceibacter* [41], or *Nonlabens* [42] and included eight tailed phages, one having a myoviral and the rest having a siphoviral morphology. However, the coverage of the class *Flavobacteriia* and the diversity of marine flavophages remains low. With the exception of the *Cellulophaga* phages, most of the other flavophages have only been briefly characterized in genome announcements.

In the context of a large project investigating bacterioplankton successions during North Sea spring bloom season, we isolated and characterized new flavophages, with the purpose of assessing their ecological impact and diversity. In total, more than 100 phage isolates were obtained, sequenced, annotated, and classified. This diverse collection is here presented in the context of virus and bacterioplankton abundances. Metagenomes obtained for Helgoland waters of different size fractions were mapped to all newly isolated flavophage genomes, testing the environmental relevance of the flavophage isolates. This study indicates that flavophages are indeed a mortality factor during spring blooms in temperate coastal seas. Furthermore it provides twelve novel phage-host systems of six genera of *Flavobacteriia*, doubling the number of known hosts.

## Material and methods

### Sampling campaigns

Surface water samples were taken off the island Helgoland at the long term ecological research station Kabeltonne (54° 11.3′ N, 7° 54.0′ E). The water depth was fluctuating from 7 to 10 m over the tidal cycle. In 2017, a weekly sampling was conducted over five weeks starting on March 14 (Julian days 73–106) and covered the beginning of a spring phytoplankton bloom. In 2018, a weekly sampling was conducted over eight weeks starting on March 29 and ending on May 24 (Julian days 88–145). It covered the full phytoplankton bloom. Additional measurements were performed to determine the chlorophyll concentration, total bacterial cell counts, absolute *Bacteroidetes* cell numbers and total virus abundances (SI file 1 text). Viruses were counted both by epifluorescence microscopy of SYBR Gold stained samples and by transmission electron microscopy (TEM) of uranyl acetate stained samples (SI file 1 text). The *Bacteroidetes* cell numbers were determined by 16S rRNA targeted fluorescence in situ hybridization (FISH) with specific probes (SI file 1 text).

### Phage isolation

Phage isolates were obtained either after an intermediate liquid enrichment step or by direct plating on host lawns (SI file 1 Table 1). In both cases, seawater serially filtered through 10, 3, 0.2 µm polycarbonate pore size filters served as phage source. To ensure purity, three subsequent isolation rounds were performed by picking single phage plaques and using them as inoculum for new plaque assays. Then, a phage stock was prepared. For more details, see SI file 1 text. These phage stocks were then used for assessing phage morphology, host range and genome size, and for DNA extraction (SI file 1 text).

### Determination of phage genomes

Phage DNA was extracted using the Wizard resin kit (Promega, Madison, USA) and eluted in TE buffer (after [43]). The DNA was sequenced on an Illumina HiSeq3000 applying the paired-end 2 × 150 bp read mode. For most *Cellulophaga* phages (except Ingeline) and *Maribacter* phages a ChIP-seq (chromatin immunoprecipitation DNA-sequencing) library was prepared. This was done to generate a NGS library for ssDNA phages and due to library preparation issues with the *Maribacter* phages for Illumina and PacBio. For the other phages a DNA FS library was prepared. The raw reads were quality trimmed and checked, then assembled using SPAdes (v3.13.0, [44]) and Tadpole (v35.14, sourceforge.net/projects/bbmap/). Assembly quality was checked with Bandage [32]. The genome ends were predicted using PhageTerm [33], but not experimentally verified. For more details about all these procedures, see SI file 1 text.

### Retrieval of related phage genomes and taxonomic assignment

Several publicly available datasets of cultivated and environmental phage genomes were queried for sequences related with the flavophages isolated in this study, in a multistep procedure (SI file 1 text). The datasets included GenBank Viral (ftp://ftp.ncbi.nlm.nih.gov/genomes/genbank/viral/, downloaded on 17.03.2021), the GOV2 dataset, IMG/VR2, as well as further environmental datasets [13, 45–50]. To determine the relationship of the new flavophages and their relatives with taxa recognized by the International Committee of Taxonomy of Viruses (ICTV), we have added these phages to larger datasets, including ICTV recognized phages (for details, see SI file 1 text).

To determine the family-level classification of the flavophages, we used VirClust ([51], www.virclust.icbm.de), ViPTree [52] and VICTOR [53] to calculate protein-based hierarchical clustering trees. For dsDNA flavophages, a VirClust hierarchical tree was first calculated for the isolates, their relatives, and the ICTV dataset. Based on this, a reduced dataset was compiled, from family level clades containing our flavophages. The reduced dsDNA flavophages dataset and the complete ssDNA flavophage dataset were further analyzed with VirClust, VipTree and VICTOR. The parameters for VirClust were: (i) protein clustering based on “evalue”, after reciprocal BLASTP hits were removed if *e*-value >0.0001 and bitscore <50; (ii) hierarchical clustering based on protein clusters, agglomeration method “complete”, 1000 bootstraps, tree cut at a distance of 0.9. The parameters for VICTOR were “amino acid” data type and the “d6” intergenomic distance formula. In addition to phylogenetic trees, VICTOR used the following predetermined distance thresholds to suggest taxon boundaries at subfamily (0.888940) and family (0.985225) level [53]. Furthermore, the web service of GRAViTy (http://gravity.cvr.gla.ac.uk, [54]) was used to determine the similarity of ssDNA phages and their relatives with other ssDNA viruses in the Baltimore Group II, *Papillomaviridae* and *Polyomaviridae* (VMRv34).

To determine the intra-familial relationships, smaller phage genome datasets corresponding to each family were analyzed using (i) nucleic acid-based intergenomic similarities calculated with VIRIDIC [55] and (ii) core protein phylogeny. The thresholds used for species and genus definition were 95% and 70% intergenomic similarity, respectively. The core protein analysis was conducted as follows: (i) core genes were calculated with the VirClust web tool [51], based on protein clusters calculated with the above parameters; (ii) duplicated proteins were removed; (iii) a multiple alignment of all concatenated core proteins was constructed with MUSCLE (v3.8.425, [56]) and (iv) used for the calculation of IQ-Trees with SH-aLRT [57] and ultrafast bootstrap values [58] using ModelFinder [59]. All phylogenetic trees were visualized using FigTree v1.4.4. [60], available at http://tree.bio.ed.ac.uk/software/figtree/).

### Phage genome annotation

All phage genomes analyzed in this study were annotated using a custom bioinformatics pipeline described elsewhere [28], with modifications (SI file 1 text). The final protein annotations were evaluated manually.

### Host assignment for environmental phage genomes

To determine potential hosts for the environmental phage genomes, several methods were used. First we did, a BLASTN [61] search (standard parameters) against the nucleotide collection (nr/nt, taxid:2, bacteria), for all phages and environmental contigs belonging to the newly defined viral families. The hit with the highest bitscore and annotated genes was chosen to indicate the host. Second, with the same genomes a BLASTN against the CRISPR/cas bacterial spacers from the metagenomic and isolate spacer database was run with standard settings using the IMG/VR website (https://img.jgi.doe.gov/cgi-bin/vr/main.cgi). Third, WIsH [62] was used to predict hosts (standard parameters) with the GEM metagenomic contig database [63] as host database.

### Detection of flavophages and their hosts in Helgoland metagenomes by read mapping

The presence of flavophages, flavobacterial hosts and environmental phages in unassembled metagenomes from the North Sea and their relative abundances were determined by read mapping, using a custom bioinformatics pipeline [28]. Two datasets were analyzed: (i) cellular metagenomes (0.2–3 µm fraction) from the spring 2016 algal bloom (SI file 1 Table 2) and (ii) cellular metagenomes (0.2–3 µm, 3–10 µm and >10 µm fractions) from the spring 2018 algal bloom (SI file 1 Table 2).

A bacterium was considered present when >60% of the genome was covered by reads with at least 95% identity. A phage was present when >75% of its genome was covered by reads with at least 90% identity. Relatives of a phage were present when >60% of its genome was covered by reads with at least 70% identity. The relative abundance of a phage/host genome in a sample was calculated by the following formula: “number of bases at ≥ x% identity aligning to the genome/genome size in bases/library size in gigabases (Gb)”.

### Host 16S rRNA analysis

The genomes of bacteria from which phages were isolated were sequenced with Sequel I technology (Pacific Biosciences, Menlo Park, USA) (SI file 1 text). After genome assembly the quality was checked and 16S rRNA operons were retrieved using the MiGA online platform [64]. Additionally, the 16S rRNA gene from all the other bacterial strains was amplified and sequenced using the Sanger technology (see SI file 1 text).

For phylogenetic analysis, a neighbor joining tree with Jukes-Cantor correction and a RAxML tree (version 8, [65]) were calculated using ARB [66]. The reference data set Ref132 was used, with the termini filter and *Capnocytophaga* as outgroup [67]. Afterwards, a consensus tree was calculated.

### CRISPR spacer search

CRISPR spacers and cas systems were identified in the host genomes by CRISPRCasFinder [68]. Extracted spacers were mapped with the Geneious Assembler to the flavophage genomes in highest sensitivity mode without trimming. Gaps were allowed up to 20% of the spacer and with a maximum size of 5, word length was 10, and a maximum of 50% mismatches per spacer was allowed. Gaps were counted as mismatches and only results up to 1 mismatch were considered for the phage assignment to the hosts used in this study.

The IMG/VR [48] web service was used to search for spacers targeting the flavophage isolates and the related environmental genomes. A BLASTN against the viral spacer database and the metagenome spacer database were run with standard parameters (e-value of 1e-5). Only hits with less than two mismatches were taken into account.

## Results

**Fig. 1:**
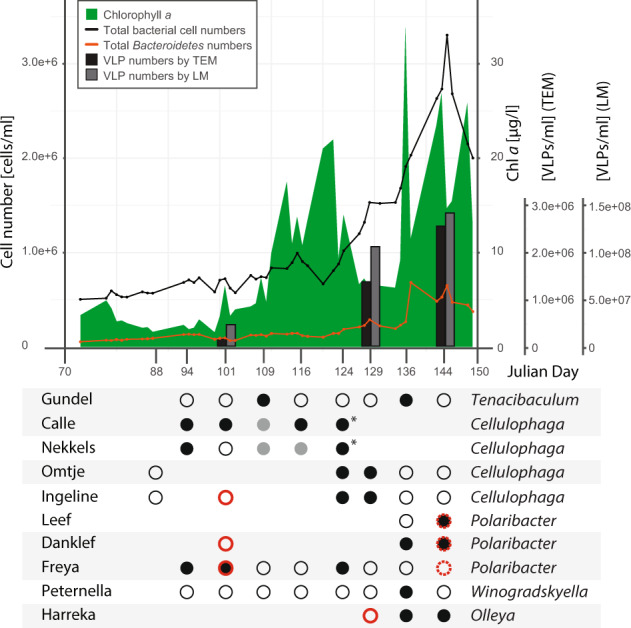
Flavophage detection during the 2018 spring phytoplankton bloom, as inferred from phage isolation and metagenome read mapping. Upper panel: Results are presented in the context of chlorophyll *a* concentration (green), total bacterial cell numbers (black line), *Bacteroidetes* numbers (orange line), phage numbers by transmission electron microscopy (TEM, black bar), and phage numbers by epifluorescence light microscopy (LM, gray bar). Lower panel: Phage isolation is shown for different time points (*x* axis, Julian days), with the phage identity verified by sequencing (black dots) or not determined (gray dots). Most of the isolations were done by enrichment, and some by direct plating (asterisks). Phage detection in metagenomes was performed by read mapping, with a 90% read identity threshold for phages in the same species (full red circles) and 70% read identity threshold for related phages (dashed red circles). Alternating gray shading of isolates indicates phages belonging to the same family.

**Fig. 2:**
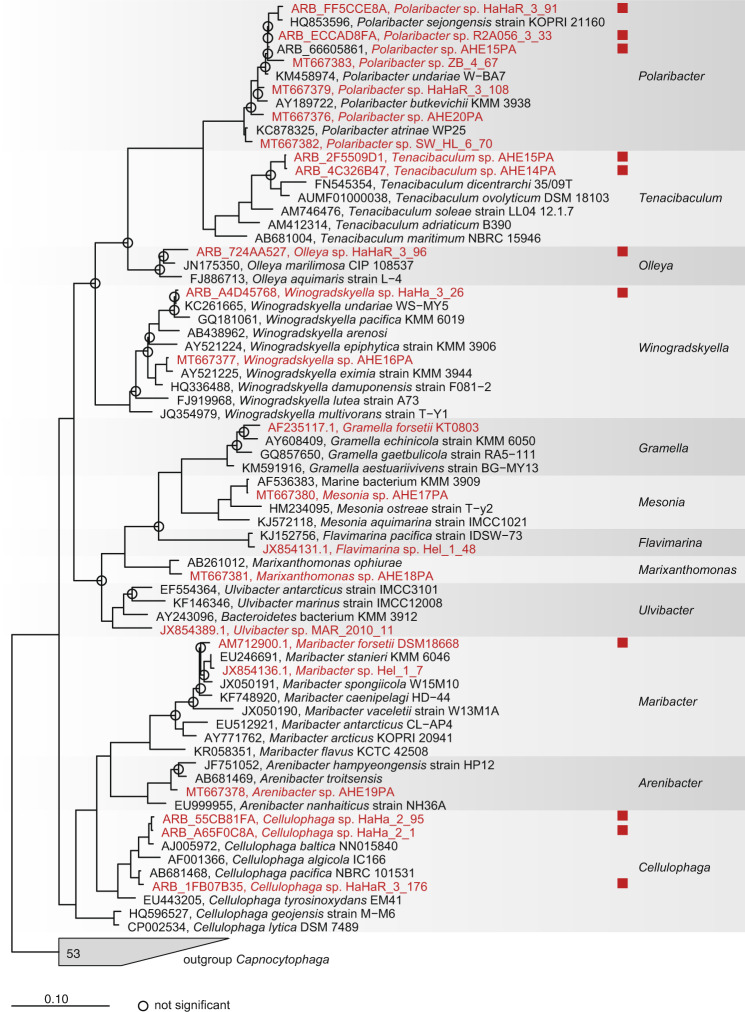
Phylogenetic tree (consensus between RAxML and neighbor-joining) of the 16S rRNA gene from all bacterial strains used to enrich for phages in 2017 and 2018 (red), plus reference genomes. Red squares indicate successful phage isolation.

Spring phytoplankton blooms were monitored by chlorophyll *a* measurements (Fig. 1, SI file 1 Fig. 1). In 2018, the bloom had two chlorophyll *a* peaks, and it was more prominent than in 2017. Diatoms and green algae dominated the 2018 bloom (SI file 1 Fig. 2). During both blooms, bacterial cell numbers almost tripled, from ~6.5 × 10^5^ cells ml^−1^ to ~2 × 10^6^ cells ml^−1^. The *Bacteroidetes* population showed a similar trend, as revealed by 16S rRNA FISH data (Fig. 1).

### Viral counts

**Fig. 3:**
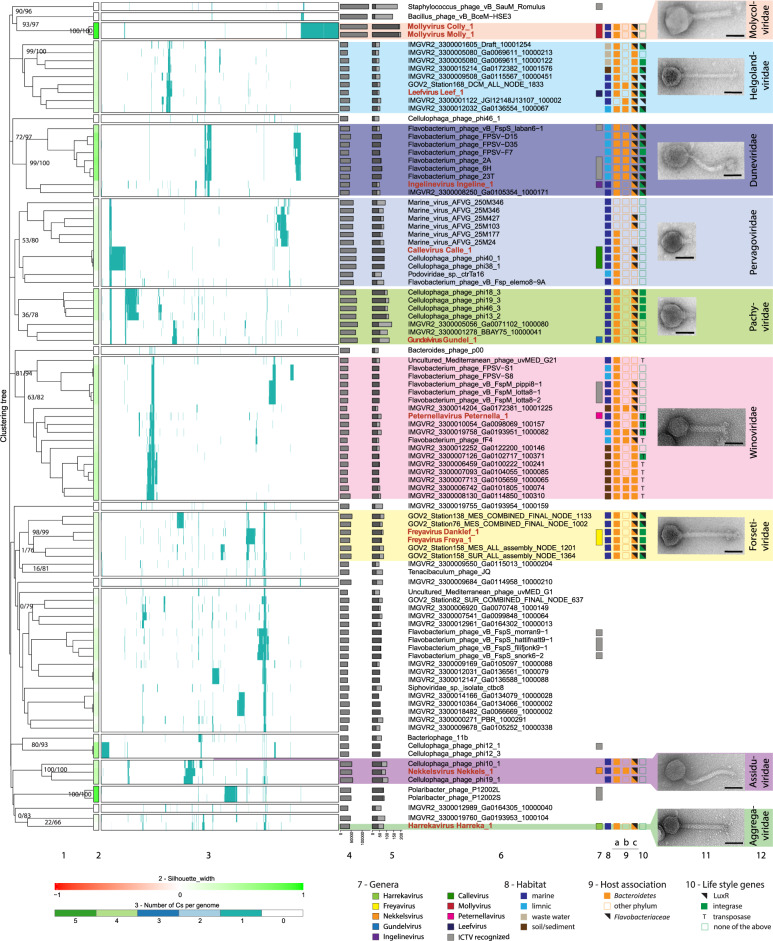
VirClust hierarchical clustering of the new dsDNA flavophages and their relatives (reduced dataset), based on intergenomic distances calculated using the protein cluster content. For an extended tree, including the larger ICTV dataset, see SI file 3. **1.** Hierarchical clustering tree. Two support values, selective inference (si, [99]) and approximately unbiased (au, [100]), are indicated at branching points (si/au) only for the major clades (see SI file 1 Figs. 6 and 7 for all support values). The tree was cut into smaller viral genome clusters (VGCs) using a 0.9 distance threshold. Each VGC containing our flavophages was proposed here as a new family. Exceptions were made for the “Aggregaviridae” and “Forsetiviridae”, for which only part of the VGC were included in the families, to exclude some genomes with lower support values. Each VGC is framed in a rectangle in 2 and 3. **2.** Silhouette width, measures how related is a virus with other viruses in the same VGCs. Similarity to other VGCs is indicated by values closer to -1 (red). Similarity to viruses in the same VGC is indicated by values closer to 1 (green). **3.** Distribution of the protein clusters (PCs) in the viral genomes. **4.** Genome length (bps). **5.** Fraction of proteins shared with other viruses (dark gray), based on protein assignment to PCs. **6.** Virus names, with flavophages isolated in this study marked in red. **7.** Genus assignment. Gray bars indicate already defined genera by the ICTV with the following names from top to bottom: *Silviavirus*, *Labanvirus*, *Unahavirus*, *Pippivirus*, *Lillamyvirus*, *Muminvirus*, *Lillamyvirus*, *Helsingorvirus*, and *Incheonvirus*. **8.** Habitat. **9.** Host association, as determined by: 9a) BlastN; 9b) CRISPR spacers; 9c) WIsH with host database GEM. **10.** Life style genes. **11.** TEM images of the new flavophages, uranyl acetate negative staining. Scale bar in each TEM image has 50 nm **12.** Newly proposed families. Full name of environmental phages in “Winoviridae” and unclassified: AP013511.1_Uncultured_Mediterranean_phage_uvMED,_group_G21,_isolate__uvMED-CGR-C117A-MedDCM-OCT-S32-C49 and, AP013358.1_Uncultured_Mediterranean_phage_uvMED,_group_G1,_isolate__uvMED-CGR-U-MedDCM-OCT-S27-C45, respectively.

**Fig. 4:**
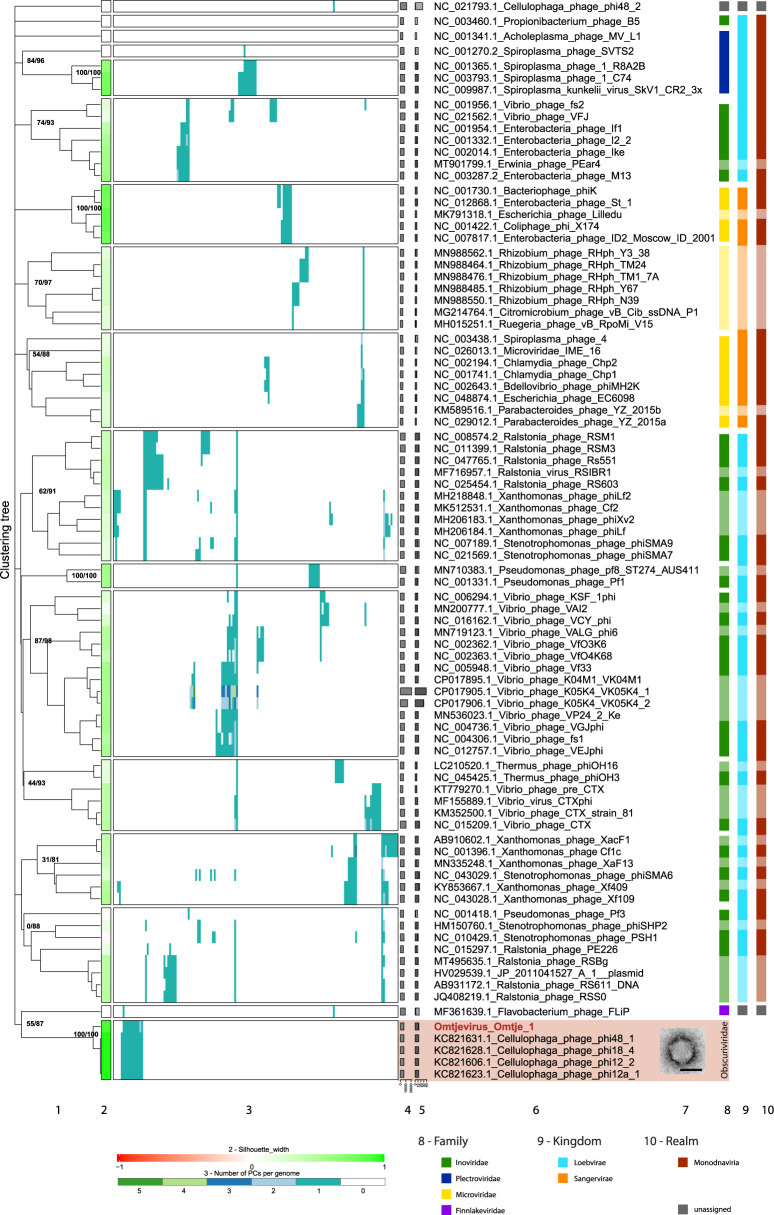
VirClust hierarchical clustering of the new ssDNA flavophages and their relatives, based on intergenomic distances calculated using the protein cluster content. **1**. Hierarchical clustering tree. Two support values, selective inference (si [99]) and approximately unbiased (au [100]) are indicated at branching points (si/au) only for the major clades (see SI file 1 Figs. 16 and 17 for all support values). The tree was cut into smaller viral genome clusters (VGCs) using a 0.9 distance threshold. Each VGC is framed in a rectangle in 2 and 3. **2**. Silhouette width, measures how related is a virus with other viruses in the same VGCs. Similarity to other VGCs is indicated by values closer to -1 (red). Similarity to viruses in the same VGC is indicated by values closer to 1 (green). **3**. Distribution of the protein clusters (PCs) in the viral genomes. **4**. Genome length (bps). **5**. Fraction of proteins shared with other viruses (dark gray), based on protein assignment to PCs. **6**. Virus names, with flavophages isolated in this study marked in red. **7**. TEM image of the new flavophage, uranyl acetate negative staining. Scale bar in TEM image has 50 nm. **8**. Family (ICTV). **9**. Kingdom (ICTV). **10**. Realm (ICTV). Lighter colors in columns 8–10 represent phages not recognized by the ICTV, but by publications.

Viral particles were counted at three time points during the 2018 bloom, both by SYBR Gold staining and TEM. Numbers determined by TEM were almost two orders of magnitude lower than those determined by SYBR Gold (Fig. 1), a typical phenomenon when comparing these two methods [69]. However, both methods showed a strong increase of viral particle numbers over the course of the bloom. All viruses counted by TEM were tailed, a strong indication that they were infecting bacteria or archaea, but not algae [70] (SI file 1 Fig. 3). The capsid size ranged between 54 and 61 nm, without any significant differences between the three time points (SI file 1 Fig. 4). The virus to bacteria ratio increased throughout the bloom, almost doubling (Fig. 1, SI file 1 Table 4).

### Flavophage isolation and classification

For phage enrichment, 23 bacterial strains previously isolated from algal blooms in the North Sea were used as potential hosts (SI file 1 Table 1). In 2017, we implemented a method for enriching flavophages on six host bacteria. A much larger and more diverse collection of 21 mostly recently isolated *Flavobacteriia* was used in 2018. A total of 108 phage isolates were obtained for 10 of the bacterial strains, either by direct plating or by enrichment (see Table 1) These were affiliated with the bacterial genera *Polaribacter*, *Cellulophaga*, *Olleya, Tenacibaculum*, *Winogradskyella*, and *Maribacter* (Fig. 2, SI file 1 Table 5).

**Table 1:** Phylogenetic characterization and isolation details of each phage group. The names have a Frisian origin, to reflect the flavophage place of isolation.

Exemplar phage abbreviation	Phage species	Phage genus	Phage family	Year of isolation	Isolation sources	Number of strains
Molly	“Mollyvirus Molly”	“Mollyvirus”	“Molycolviridae”	2017	Enrichment	6
Colly	“Mollyvirus Colly”	“Mollyvirus”	“Molycolviridae”	2017	Enrichment	1
Gundel	“Gundelvirus Gundel”	“Gundelvirus”	“Pachyviridae”	2018	Enrichment	1
Calle	“Callevirus Calle”	“Callevirus”	“Pervagoviridae”	2018	Enrichment, direct plating	3
Nekkels	“Nekkelsvirus Nekkels”	“Nekkelsvirus”	“Assiduviridae”	2018	Enrichment, direct plating	2
Omtje	“Omtjevirus Omtje”	“Omtjevirus”	“Obscuriviridae”	2017 & 2018	Enrichment	5
Ingeline	“Ingelinevirus Ingeline”	“Ingelinevirus”	“Duneviridae”	2017 & 2018	Enrichment	8
Leef	“Leefvirus Leef”	“Leefvirus”	“Helgolandviridae”	2018	Enrichment	1
Danklef	“Freyavirus Danklef”	“Freyavirus”	“Forsetiviridae”	2018	Enrichment	5
Freya	“Freyavirus Freya”	“Freyavirus”	“Forsetiviridae”	2018	Enrichment	10
Peternella	“Peternellavirus Peternella”	“Peternellavirus”	“Winoviridae”	2018	Enrichment	1
Harreka	“Harrekavirus Harreka”	“Harrekavirus”	“Aggregaviridae”	2018	Enrichment	1

Intergenomic similarities at the nucleic acid level allowed the grouping of the 108 flavophages into 44 strains (100% similarity threshold) and 12 species (95% similarity threshold) (SI file 2). A summary of the new phage species and their exemplar isolate phage, including their binomial name and isolation data, is found in Table 1. For brevity, we are mentioning here only the short exemplar isolate phage names of each new species: Harreka, Gundel, Molly, Colly, Peternella, Danklef, Freya, Leef, Nekkels, Ingeline, Calle and Omtje.

**Fig. 5:**
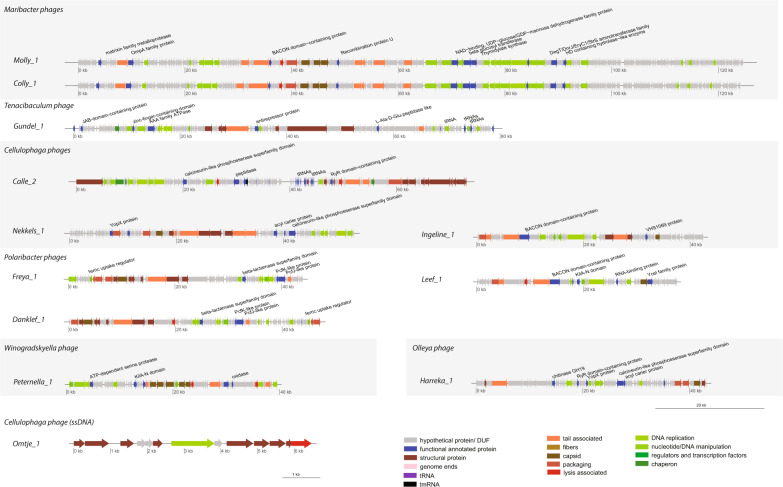
Genome maps of phage isolates with color-coded gene annotations. Genomes of one representative of each new phage species are shown and depicted at the same scale to allow phage genome size comparisons. Due to its small genome Omtje is not at the same scale. Genes are color-coded by annotation and specific functional annotated proteins are indicated at genomes. Alternating gray shading indicates phages of the same host genus.

Virion morphology as determined by TEM showed that 11 of the exemplar phages were tailed (Fig. 3). According to the new megataxonomy of viruses, tailed phages belong to the realm *Duplodnaviria*, kingdom *Heunggongvirae*, phylum *Uroviricota*, class *Caudoviricetes*, order *Caudovirales* [71]. The only non-tailed, icosahedral phage was Omtje (Fig. 4). Further digestion experiments with different nucleases showed that Omtje has a ssDNA genome (SI file 1 Fig. 5).

Hierarchical clustering with VirClust placed the new dsDNA, tailed flavophages into 9 clades of similar rank with the current eleven *Caudovirales* families (Fig. 3, SI file 1 Figs. 6 and 7, SI file 3). Similar clades were obtained with VICTOR and ViPTree (SI file 1 Figs. 8 and 9). These clades formed individual clusters when the VirClust tree was cut at a 0.9 distance threshold (Fig. 3), which was shown to delineate the majority of *Caudovirales* families [51]. In agreement, these clades were assigned to different subfamilies by the VICTOR analysis (SI file 1 Fig. 8), which, in the light of the current changes in the ICTV phage classification, represent different families [72]. Therefore, we are proposing that the 9 clades correspond to 9 new families, which we tentatively named “Helgolandviridae”, “Duneviridae”, “Winoviridae”, “Molycolviridae”, “Pachyviridae”, “Pervagoviridae” “Assiduviridae”, “Forsetiviridae” and “Aggregaviridae” (Fig. 3). The core proteins for each family consisted mainly of structural proteins (Table 2). With the exception of few proteins from “Pachyviridae” and “Pervagoviridae”, the core proteins were not shared between the families (they belonged to separate protein clusters, see Fig. 3 and Table 2). Four of the new families (“Aggregaviridae”, “Forsetiviridae”, “Molycolviridae” and “Helgolandviridae”) were formed only from new flavophages and from environmental phage genomes (Fig. 3, SI file 1 Fig. 8). The remaining five families also contained previously cultivated phages, infecting bacteria from the genus *Cellulophaga* (“Pervagoviridae”, “Pachyviridae” and “Assiduviridae”) and *Flavobacterium* (“Duneviridae”, “Winoviridae”). Part of the cultivated flavobacterial phages in the “Duneviridae” and “Winoviridae” are currently classified by ICTV in three genera in the families *Siphoviridae* and *Myoviridae*. However, because the *Siphoviridae* and *Myoviridae* families are based on phage morphologies, they are being slowly dissolved and split into new families, based on sequence data [73].

**Table 2:** Core genes of the newly defined families.

Family	Total core genes	Core gene annotation	Number of core genes used for phylogeny	Core genes used for phylogeny
“Forsetiviridae”	8	Tape measure protein, major capsid protein, portal protein, structural proteins (5)	7	Tape measure protein, major capsid protein, portal protein, structural proteins (4)
“Winoviridae”	10	Clp protease, neck protein, major capsid protein, sheath, phage protein D, head morphogenesis protein/portal protein, hp (4)	9	Clp protease, neck protein, major capsid protein, sheath, phage protein D, hp (4)
“Pachyviridae”	11	Portal protein^a^, sheath, structural protein^b^ (5), hp^c^ (4)	9	Portal protein, structural protein (4), hp (4)
“Pervagoviridae”	8	Portal protein^a^, chaperonin cpn10, structural protein^b^ (4), hp^c^ (2)	3	Chaperonin cpn10, structural protein (2)
“Duneviridae”	9	Major capsid protein, portal protein, adaptor protein, hp (5)	7	Major capsid protein, adaptor protein, hp (4)
“Helgolandviridae”	6	hp (6)	5	hp (5)
“Assiduviridae”	33	Phage tail tape measure protein, DNA primase/helicase, NinG recombination protein, pectate lyase (2), structural protein, hp (27)	n.d.	
“Obscuriviridae”	11	Replication initiation factor, mannosyl-glycoprotein endo-beta-N-acetylglucosaminidase, structural protein (8), hp	10	Replication initiation factor, mannosyl-glycoprotein endo-beta-N-acetylglucosaminidase, structural protein (8)

^a^The portal proteins are shared (belongs to the same protein cluster) between *Pachyviridae* and *Pevagoviridae.*

^b^Two structural proteins each are shared (belong to the same protein cluster) between *Pachyviridae* and *Pevagoviridae.*

^c^One hypothetical protein is shared (belongs to the same protein cluster) between *Pachyviridae* and *Pevagoviridae.*

Number of core genes with the same annotation are indicated in parentheses.

Using a 70% threshold for the intergenomic similarities at nucleotide level indicated, that Harreka, Nekkels, Gundel, Peternella, Leef and Ingeline phages form genera on their own, tentatively named here “Harrekavirus”, “Nekkelsvirus”, “Gundelvirus”, “Peternellavirus”, “Leefvirus” and “Ingelinevirus”. The other new flavophages formed genera together with isolates from this study or with previously isolated flavophages, as follows: the genus “Freyavirus” formed by Danklef and Freya, the genus “Callevirus” formed by Calle, Cellulophaga phage phi38:1, Cellulophaga phage phi40:1, and the genus “Mollyvirus” formed by Molly and Colly (SI file 4). The assignment to new genera was supported by the core proteins phylogenetic analysis (SI file 1 Figs. 10–15).

Hierarchical clustering using VirClust (Fig. 4, SI file 1 Figs. 16 and 17), ViPTree (SI file 1 Fig. 18), and VICTOR (SI file 1 Fig. 19) of a dataset including Omtje and all related and reference ssDNA phages showed that Omtje is clustering with previously isolated ssDNA phages infecting *Cellulophaga*, separately from other ssDNA phage families, the *Microviridae, Inoviridae*, and *Plectroviridae*. This was supported also by GRAViTy (SI file 5). Only one protein cluster was shared outside this cluster, with Flavobacterium phage FliP (Fig. 4), even when forming protein-superclusters based on HMM similarities (SI file 1 Fig. 20). We propose here that this cluster represents a new family, tentatively called here “Obscuriviridae”. The placement of this family into higher taxonomic ranks, including the realm, remains to be determined in the future. Intergenomic similarity calculations (SI file 6), supported by core gene phylogenetic analysis (SI file 1 Fig. 21), indicate that Omtje forms a genus by itself, tentatively named here “Omtjevirus”.

### Features of the new flavophage isolates

#### Phages of *Polaribacter*

Three *Polaribacter* phages were obtained: (i) Danklef and Freya, part of the family “Forsetiviridae”, infected *Polaribacter* sp. R2A056_3_33 and HaHaR_3_91, respectively; and (ii) Leef, part of the family “Helgolandviridae”, infected *Polaribacter* sp. AHE13PA (SI file 1 Fig. 1). These phages infected only their isolation host (SI file 1 Fig. 22). They all had a siphoviral morphology (Fig. 3 and Table 3).

**Table 3:** Phage characteristics of each phage group. Because Colly has the same characteristics as Molly, it is not especially mentioned in the table. Lysis genes are stated as N-acetylmuramidase (M), N-acetylmuramoyl-L-alanine-amidase (A), Glycosid Hydrolase 19 (GH), L-alanine-D-glutamine-peptidase (P), spanin (S), holin (H).

Phage genus	Host genus	Genome size [kbp]	%GC	ORFs	# tRNAs	# tmRNAs	# integrases	Lysis genes	Genome ends	Circularly closed	Capsid diameter [nm]	Tail length [nm]	Tail width [nm]	Morphology
Molly	*Maribacter*	124.2	36.2	193–201	0	0	0	A	n.d.	yes	74.9 ± 3.6	101.5 ± 6.3	18.1 ± 1.6	myoviridal
Gundel	*Tenacibaculum*	78.5	30.4	118	10	0	0	P	short DTRs	yes	60.5 ± 5.2	22.7 ± 3.2	n.a.	podoviridal
Calle	*Cellulophaga*	73.0	38.1	85–86	20	1	0	A	n.d.	yes	60.3 ± 3.0	23.0 ± 5.5	13.5 ± 2.4	podoviridal
Nekkels	*Cellulophaga*	53.3–54.3	31.5	93–94	0	0	0	GH, S	n.d.	yes	57.8 ± 5.0	141.0 ± 7.9	13.0 ± 1.5	siphoviridal
Omtje	*Cellulophaga*	6.6	31.2	13	0	0	0	A	n.d.	yes	52.3 ± 4.6	n.a.	n.a.	non tailed
Ingeline	*Cellulophaga*	42.6	32.2	49–50	0	0	2	S	n.d.	yes	59.0 ± 5.3	132.9 ± 19.3	11.2 ± 1.7	siphoviridal
Leef	*Polaribacter*	37.5	29.7	48	0	0	2	M	Cos 3'	yes	49.2 ± 3.6	138.7 ± 9.6	11.1 ± 2.0	siphoviridal
Danklef	*Polaribacter*	47.2–48.2	28.9	76–78	1	0	1	M	n.d.	yes	46.1 ± 2.2	157.4 ± 4.6	12.1 ± 1.8	siphoviridal
Freya	*Polaribacter*	44.0–48.9	28.9	66–78	0	0	1	0	n.d.	yes	53.9 ± 4.7	151.0 ± 8.2	13.1 ± 2.0	siphoviridal
Peternella	*Winogradskyella*	39.6	35.3	62	0	0	1	A, H	Mu-like	no	52.3 ± 4.2	105.8 ± 7.4	16.4 ± 2.4	myoviridal
Harreka	*Olleya*	43.2	32.0	80	0	0	0	GH	n.d.	yes	44.3 ± 3.6	123.8 ± 8.0	14.3 ± 2.0	myoviridal

Genome ends were predicted with PhageTerm. Due to sequencing method and ambiguous results not for all phage genomes ends were determined (n.d.). For Omtje and Gundel, having no tail and a very pointy tail, respectively, the tail length and tail width measurements were not applicable (n.a.).

The genome size ranged between ~38 kbp and ~49 kbp. The G + C content was very low (28.9–29.7%). For Leef, PhageTerm predicted genome ends of type Cos 3′ (Table 3). Three types of structural proteins were identified in all phages, a major capsid protein, a tail tape measure protein, and a portal protein. Several genes for DNA replication, modification and nucleotide metabolism genes were found (Fig. 5 and SI file 7). An N-acetylmuramidase, potentially functioning as an endolysin, was detected in Danklef and Leef, surrounded by transmembrane domain (TMD) containing proteins. All three phages encoded an integrase, and thus have the potential to undergo a temperate lifestyle. Leef had also a LuxR protein, which is a quorum-sensing dependent transcriptional activator, and a pectin lyase.

#### Phages of *Cellulophaga*

Four Cellulophaga phages were obtained: (i) Calle, part of the family “Pervagoviridae”, infected *Cellulophaga* sp. HaHa_2_95; (ii) Nekkels, part of the family “Assiduviridae”, infected *Cellulophaga* sp. HaHa_2_1; (iii) Ingeline, part of the family “Duneviridae”, and Omtje, part of the family “Obscuriviridae”, infected *Cellulophaga* sp. HaHaR_3_176 (SI file 1 Fig. 1). Nekkels and Calle also infected other bacterial strains than their isolation host, although at a low efficiency: *Polaribacter* sp. AHE13PA (Nekkels) and *Cellulophaga* sp. HaHa_2_1 (Calle) (SI file 1 Fig. 22). The virion morphology varied from icosahedral, non-tailed, microvirus-like for Omtje, to tailed, podovirus-like for Calle and siphovirus-like for Nekkels and Ingeline (Fig. 3 and Table 3).

The genome size ranged from ~6.5 kbp (Omtje) to ~73 kbp (Calle). The G + C content varied between 31.2 and 38.1% (Table 3). The few structural genes recognized were in agreement with the virion morphology (Fig. 5). For example, Ingeline and Nekkels had a tape measure protein, and a portal protein was found in all three-tailed phages. From the replication genes, we detected in Omtje a replication initiation protein specific for ssDNA phages, and in Calle a DNA polymerase A (Fig. 5).

Potential endolysins found were the mannosyl-glycoprotein endo-beta-N-acetylglucosaminidase in Omtje and the N-acetylmuramoyl-L-alanine-amidase in Calle. The latter had in its vicinity two proteins with a TMD. In Ingeline we annotated a unimolecular spanin. Nekkels encoded a glucoside-hydrolase of the GH19 family, with a peptidoglycan-binding domain. In its vicinity there were three proteins with three to six TMDs and one potential unimolecular spanin (Table 3, SI file 7).

Pectin and chondroitin/alginate lyase domains were found in Ingeline and Nekkels, as part of bigger proteins, also carrying signaling peptides. Nekkels also encoded a Yersinia outer protein X (YopX), a potential virulence factor against eukaryotes. Ingeline encoded an integrase and a LuxR gene, pointing toward a potential temperate life style. Calle had 20 tRNAs and one tmRNA gene (Fig. 5, SI file 7).

#### Phages infecting other *Flavobacteriaceae*

Five phages were obtained from four other flavobacterial hosts: (i) Harreka, part of “Aggregaviridae”, infected *Olleya* sp. HaHaR_3_96; (ii) Peternella, part of “Winoviridae”, infected *Winogradskyella* sp. HaHa_3_26; (iii) Gundel, part of “Pachyviridae”, infected *Tenacibaculum* sp. AHE14PA and AHE15PA; and (iv) Molly and Colly, part of “Molycolviridae”, infected *Maribacter forsetii* DSM18668 (Fig. 3). Harreka infected also *Tenacibaculum* sp. AHE14PA and AHE15PA with a significantly lower infection efficiency (SI file 1 Fig. 22). All virions were tailed, with a podoviral morphology for Gundel, and a myoviral morphology for Molly, Peternella and Harreka (Table 3). The genome size ranged from ~40 kbp to ~125 kbp and the G + C content between 30.4 and 36.2%. For Gundel short direct terminal repeats were predicted as genome ends (Table 3).

In these phages we recognized several structural, DNA replication and modification, and nucleotide metabolism genes (Fig. 5). With respect to replication, Molly and Colly had a DNA polymerase I gene, plus a helicase and a primase. In Gundel and Harreka we only found a DNA replication protein. In Peternella we found a MuA transposase, several structural genes similar to the Mu phage [74], and hybrid phage/host genome reads, indicating that Peternella is likely a transducing phage.

Potential endolysins were a N-acetylmuramoyl-L-alanine-amidase in Molly and Peternella and a L-alanine-D-glutamine-peptidase in Gundel. Harreka had a glycoside-hydrolase of the GH19 family. In the genomic vicinity of the potential lysins, several proteins having 1–4 TMDs were found, including a holin in Peternella (Table 3, SI file 7).

Additional features of these phages were: (i) ten tRNA genes in Gundel; (ii) a relatively short (199 aa) zinc-dependent metallopeptidase, formed from a lipoprotein domain and the peptidase domain in Molly, and (iii) a YopX protein in Harreka (Fig. 5).

### Environmental phage genomes

Six of the nine proposed new families (“Forsetiviridae”, “Pachyviridae”, “Pervagoviridae”, “Winoviridae”, “Helgolandviridae” and “Duneviridae”) include members for whose genomes were assembled from environmental metagenomes (Fig. 3). We have briefly investigated which bacterial groups are potential hosts for these phages. Most of them gave BLASTN hits with a length between 74 and 5844 bases with bacterial genomes from the *Bacteroidetes* phylum (Fig. 3, SI file 1 Tables 6 and 7), likely due to the presence of prophages and horizontal gene transfer events. Some of the environmental viral genomes gave *Bacteroidetes* associated hits against the metagenome CRISPR spacer database (SI file 1 Table 8). The host prediction using WIsH supported the results by BLASTN and CRISPR spacer matching, and predicted members of *Bacteroidetes* as hosts for most of the environmental viral genomes. Some hosts were identified down to the family level, as *Flavobacteriaceae* (SI file 1 Table 9). Only phages in “Winoviridae”, “Pervagoviridae” and “Helgolandviridae” had other families than *Flavobacteriaceae* as hosts, but all in the *Bacteroidetes* phylum (Fig. 3). In addition, several marine contigs from the “Helgolandviridae” contained *Bacteroidetes* Associated Carbohydrate-binding Often N-terminal (BACON) domains (SI file 1 Table 10). Together, these results suggest that most of the environmental viral genomes in the new families are infecting members of the phylum *Bacteroidetes*.

Integrase encoding genes were found on all environmental phages from the “Forsetiviridae”, and some of the genomes from “Winoviridae”, “Pachyviridae”, “Helgolandviridae” and “Duneviridae”. LuxR encoding genes were found in many genomes from “Helgolandviridae”, “Duneviridae” and “Forsetiviridae”, and one had also a transporter for the auto-inducer 2 (Fig. 3, SI file 1 Table 10). In the “Winoviridae”, all phages encoded Mu-like structural proteins, including the MuD terminase, and two environmental viral genomes also encoded a MuA transposase.

### Flavophages in the environment

#### CRISPR/Cas spacers indicate flavophage presence in the environment

CRISPR/Cas systems were identified in *Polaribacter* sp. HaHaR_3_91 and *Polaribacter* sp. R2A056_3_33 genomes. Spacers from the first strain matched Freya genomes. From the second strain, several spacers matched Danklef genomes, one matched Freya and another Leef (SI file 1 Table 11). This shows that Freya, Danklef and Leef, or their relatives, have infected *Polaribacter* strains in the Helgoland sampling site before 2016, when the host *Polaribacter* strains were isolated. Spacers matching Nekkels were found in a metagenome of a *Rhodophyta* associated bacterial community (SI file 1 Table 7), showing the presence of Nekkels or its relatives in this habitat.

#### Read mapping for phages and hosts show presence in North Sea waters

To assess the presence and dynamics of flavophages in the North Sea, we mined by read mapping cellular metagenomes (>10 µm, 3–10 µm, 0.2–3 µm) from the 2016 and 2018 spring blooms. We found five of the new flavophages in the cellular metagenomes from the 2018 spring phytoplankton bloom, at three different time points (Table 4). The complete genomes of Freya, Harreka and Ingeline were covered by reads with 100% identity, signifying that these exact phage isolates were present in the environment. About 85% from Danklef’s genome was covered with reads having 100% identity, indicating that close relatives of this phage (e.g., same species) were present. The genome of Leef was covered only 62% with reads of >70% identity, suggesting that more distant relatives (e.g., genus level) were detected. All phages and their relatives were exclusively found in the >3 µm and >10 µm metagenomes. The most abundant flavophages were Freya and Danklef, reaching 53.8 and 10.4 normalized genome coverage, respectively.

**Table 4:** Read mapping results from 2018 metagenomes for isolated flavophages and their hosts.

Julian day/Gregorian date	Fraction	Phage		Host		Phage/Host ratio
		Name	Norm. genome coverage	Name	Norm. genome coverage	
102/12.04.2018	10 µm	Freya^a^	53.8	*Polaribacter* sp. HaHaR_3_91 and R2A056_3_33	0.14	384
		Freya relatives	63.3			454
		Danklef	10.4			74
		Danklef relatives	45.8			327
		Ingeline^a^	0.7	not detected	-	-
		Ingeline relatives	0.8			-
		not detected	-	*Olleya* sp. HaHaR_3_96	0.05	-
116/26.04.2018	10 µm	not detected	-	*Polaribacter* sp. HaHaR_3_91 and R2A056_3_33	0.06	-
		not detected	-	*Olleya* sp. HaHaR_3_96	0.03	-
122/02.05.2018	0.2 µm	not detected	-	*Polaribacter* sp. HaHaR_3_91 and R2A056_3_33	0.07	-
		not detected	-	*Olleya* sp. HaHaR_3_96	0.05	-
128/08.05.2018	3 µm	Harreka^a^	1.8	not detected	-	-
		Harreka relatives	5.7			-
142/22.05.2018	10 µm	Freya relatives	0.04	*Polaribacter* sp. HaHaR_3_91, R2A056_3_33 and AHE13PA	0.46	0.09
		Danklef relatives	0.03			0.07
		Leef relatives	0.02			0.04

^a^Indicates those phages covered by reads with 100% identity over the whole genome length.

Further, we searched for the presence of the five flavophage hosts in the 2018 spring bloom (Table 4). *Polaribacter* sp. was found in the >10 µm and 0.2–3 µm fractions, at different time points during the bloom, including the same two samples in which its phages (Freya, Danklef, and Leef) and their relatives were detected. At the beginning of the bloom, in April, significantly more phages than hosts were present in the >10 µm fraction. The phage/host genome ratio was 384 for Freya, and 74 for Danklef. One and a half months later, only relatives of Freya, Danklef, and Leef were found, the phage/host ratio being lower than 0.1. Discrimination between the different *Polaribacter* strains was not possible, due to their high similarity (>98% ANI). *Olleya* sp. was found in the 0.2–3 µm and >10 µm fractions, at low abundances and dates preceding the detection of its phage, Harreka. *Cellulophaga* sp., the host for Ingeline, was not found (SI file 1 Tables 12–16).

## Discussion

We have performed a cultivation-based assessment of the diversity of flavophages potentially modulating *Flavobacteriia*, a key group of heterotrophic bacteria, in the ecological context of spring bloom events in two consecutive years. In contrast to high-throughput viromics-based studies, our approach enabled us to link phages to their host species and even to strains.

Highly diverse flavophages, belonging to two distinct viral realms were isolated. As a point of reference, a viral realm is the equivalent of a domain in the cellular world [71]. Furthermore, four of the ten new families, and nine of the ten new genera had no previously cultivated representatives. This study not only uncovers a novel flavophage diversity, but also, structures a substantial part of the known marine flavophage diversity into families. These novel flavophage families are relevant not only for the marine environment. Besides cultivated flavophages, six of the families also include environmental phages from marine, freshwater, wastewater, and soil samples, which most likely infect *Bacteroidetes*.

During the phylogenetic analysis we have worked closely with ICTV members, to ensure a good quality of the phage taxonomic affiliations. Two taxonomic proposals for the new defined taxa are being submitted, one for flavophages in *Duplodnaviria* and one for “Obscuriviridae”.

Genomic analysis indicates that the new flavophages have various life styles and diverse replication strategy characteristics. Some families are dominated by potentially temperate phages, and others by potentially strictly lytic phages, as indicated by the presence/absence of integrases. Genome replication can take place (i) through long concatemers [75] (Gundel and Leef), (ii) replicative transposition [76] (Peternella), and (iii) the rolling circle mechanism [77] (Omtje).

The lysis mechanism in the new dsDNA flavophages likely follows the canonical holin/endolysins model, as suggested by the lack of membrane binding domains in the potential endolysins. Harreka and Nekkels do not encode easily recognizable lysis enzymes. Instead, they encode each a GH19. Usually, this hydrolase family is known for chitin degradation yet peptidoglycan may also be degraded [78]. A phage GH19 expressed in *Escherichia coli* caused cellular lysis [79]. Furthermore, in Harreka and Nekkels, the vicinity with potential holins, antiholins, and spanin, and the peptidoglycan-binding domain in Nekkels, suggest that the GH19 proteins of these two phages likely function as endolysins and degrade bacterial peptidoglycan. It cannot be excluded though that these enzymes have a dual function. Once released extracellularly due to lysis, the endolysins could degrade chitin, an abundant polysaccharide in the marine environment, produced for example by green-algae or copepods [80].

The presence of lyases in the genomes of Leef (pectin lyase), Ingeline (pectin lyase) and Nekkels (pectin and alginate lyases) suggests that their bacterial hosts, *Polaribacter* sp. and *Cellulophaga* sp., are surrounded by polysaccharides. In the marine environment, both alginate and pectin, which are produced in large quantities by micro- and macro-algae [81–83], serve as substrate for marine *Flavobacteriia*, especially *Polaribacter* [84–86]. Bacteria are not only able to degrade these polysaccharides, but also to produce them and form capsules or an extracellular matrix of biofilms [87–89]. In phages, such polysaccharide degrading enzymes are usually located on the tails, as part of proteins with multiple domains to reach the bacterial cell membrane. Another enzyme potentially degrading proteins in the extracellular matrix was the zinc-dependent metallopeptidase of Molly, a “Mollyvirus”, which infected a *Maribacter* strain. By depolymerizing the extracellular matrix surrounding the cells, lyases and peptidases help the phage to reach the bacterial membranes for infection or allow the new progeny to escape the cell debris and the extracellular matrix [90, 91]. It remains to be proven if phages carrying these enzymes contribute significantly to the degradation of algal excreted polysaccharides, as a byproduct of their quest to infect new bacterial cells.

Previous studies indicate that flavobacteriia can exhibit a surface-associated life style [92]. Our results paint a similar picture. For example, we detected phages for *Polaribacter, Cellulophaga* and *Olleya*, as well as the *Polaribacter* and *Olleya* genomes themselves in the particulate fraction of the cellular metagenomes. Therefore, it is likely that these bacteria are associated with particles, protists, phytoplankton or zooplankton. Spacers in metagenomes matching Nekkels, a *Cellulophaga* phage, suggest an association with red macro-algae (SI file 1 Table 7). An association with eukaryotes is also supported by the presence of YopX proteins in Harreka, infecting *Olleya*, and in all three “Assiduviridae” phages infecting *Cellulophaga*. The ability for an attached lifestyle is indicated by scanning electron microscopy images of *Cellulophaga* sp. HaHaR_3_176 cells showing high amounts of extracellular material (SI file 1 Fig. 23).

The presence of integrases and LuxR genes in phages from the “Helgolandviridae” and “Duneviridae” (Fig. 3 and SI file 1 Table 10) suggests that they likely respond to changes in the host cell densities, for example by switching from the temperate to the lytic cycle, as recently observed in *Vibrio cholerae* (pro)-phages [93, 94]. This type of quorum-sensing response would make sense in a habitat in which the host cells can achieve high densities, as for example in association with planktonic organisms or particles.

All of our flavophages, except Molly and Colly, replicated successfully at different times throughout the 2018 phytoplankton bloom. The isolation by enrichment or direct-plating indicated their presence in the viral fraction, and the retrieval by read-mapping indicated a likely presence inside the host or association with surfaces. Calle and Nekkels, infecting *Cellulophaga*, although not detected in the cellular metagenomes, were present in high abundance (at least 100 plaque-forming units ml^−1^) in the viral fraction of our samples, as revealed by successful isolation by direct-plating without previous enrichment. This apparent gap between the presences in either the viral or cellular fraction, could be either explained by phage lysis prior the metagenome collection, or by the fact that their host habitat was not sampled for metagenomics. A potential explanation is that members of the genus *Cellulophaga* are known to grow predominantly on macro-algae [95]. The detection of Omtje, Peternella, and Gundel in enrichment cultures, but not by direct-plating or in the cellular metagenomes, indicate that their presence in the environment is low. Further investigations of the specific habitat of both phage and host are necessary to confirm these findings.

For flavophages with temperate potential, the ratio between phage and host normalized read abundance can be used to predict their lytic or temperate state in the environmental samples. *Cellulophaga*, the host of Ingeline, was not detected throughout the spring bloom. However, because Ingeline was detected presumably in the particle fraction and thus might be inside its host, we can hypothesize that either its host was in a low abundance, or its genome was degraded under the phage influence. Either way, it points toward Ingeline being in a lytic cycle at the time of detection. For Freya, Danklef, and their relatives, the high phage to host genome ratios (as high as 454×) from April suggest that these phages were actively replicating and lysing their cells. In contrast, when they reappeared in May, these phages were not only three orders of magnitude less abundant than in April, but also approximately ten times less abundant than their host. This indicates that in May, only a small proportion of the *Polaribacter* cells were infected with relatives of Freya and Danklef, either in a lytic or a temperate state.

A temporal dynamics in phage populations is also reflected in the appearance of Freya and Gundel. Moreover, it is reminiscent of the boom and bust behavior previously observed with T4-like marine phages [96]. In addition, the read mapping results indicated a change in dominant phage genotypes, from the same species and even strain with Danklef and Freya in April, towards more distant relatives at the genus or even family level in May. This suggests a selection of resistant *Polaribacter* strains after the April infection and differs from the genotypic changes previously observed for marine phages, which were at the species level [97]. Persistence over longer times in the environment was shown for *Polaribacter* phages by the CRISPR/Cas spacers, and for Omtje and Ingeline, by their isolation in two consecutive years.

The North Sea flavophages isolated in this study display a very high taxonomic, genomic and life style diversity. They are a stable and active part of the microbial community in Helgoland waters. With their influence on the dynamics of their host populations by either lysis or with regard to specific bacterial population by substrate facilitation, they are modulating the carbon cycling in coastal shelf seas. The increase in bacterial numbers, reflected in the phage numbers and the ratio of phage to bacterial cells, indicate that phages actively replicate through the 2018 phytoplankton bloom, matching previous observations of the North Sea microbial community [98]. Our read mapping data indicate complex dynamics, which can now be further investigated with a large number of valuable novel phage-host systems obtained in this study.

## Supplementary information

SI_File_1

SI_File_2

SI_File_3

SI_File_4

SI_File_5

SI_File_6

SI_file_7

## Data Availability

Sequencing data and phage genomes are available at the National Center for Biotechnology Information (NCBI) with the accession number PRJNA639310 (for details see SI file 1 Tables 1 and 3). Phages (DSM111231-111236, DSM111238-111241, DSM111252, and DSM111256-111257) and bacterial hosts (DSM111037-111041, DSM111044, DSM111047-111048, DSM111061, and DSM111152) were deposited at the German Collection of Microorganisms and Cell Cultures GmbH, Braunschweig, Germany.

## References

[1] Proctor LM, Fuhrman JA (1990). Viral mortality of marine bacteria and cyanobacteria. Nature..

[2] Steward G, Wikner J, Cochlan W, Smith D, Azam F (1992). Estimation of virus production in the sea: II. field results. Mar Microb Food Webs.

[3] Suttle CA (1994). The significance of viruses to mortality in aquatic microbial communities. Microb Ecol.

[4] Thingstad TF (2000). Elements of a theory for the mechanisms controlling abundance, diversity, and biogeochemical role of lytic bacterial viruses in aquatic systems. Limnol Oceanogr.

[5] Wilhelm SW, Suttle CA (1999). Viruses and nutrient cycles in the sea: viruses play critical roles in the structure and function of aquatic food webs. BioScience..

[6] Breitbart M, Thompson LR, Suttle CA, Sullivan MB (2007). Exploring the vast diversity of marine viruses. Oceanography.

[7] Coutinho FH, Silveira CB, Gregoracci GB, Thompson CC, Edwards RA, Brussaard CPD (2017). Marine viruses discovered via metagenomics shed light on viral strategies throughout the oceans. Nat Commun.

[8] Touchon M, Moura de Sousa JA, Rocha EPC (2017). Embracing the enemy: the diversification of microbial gene repertoires by phage-mediated horizontal gene transfer. Curr Opin Microbiol.

[9] Knowles B, Silveira CB, Bailey BA, Barott K, Cantu VA, Cobián-Güemes AG (2016). Lytic to temperate switching of viral communities. Nature..

[10] Yamada Y, Tomaru Y, Fukuda H, Nagata T. Aggregate formation during the viral lysis of a marine diatom. Front Mar Sci. 2018;5.

[11] Nissimov JI, Vandzura R, Johns CT, Natale F, Haramaty L, Bidle KD (2018). Dynamics of transparent exopolymer particle production and aggregation during viral infection of the coccolithophore, *Emiliania huxleyi*. Environ Microbiol.

[12] Bergh Ø, BØrsheim KY, Bratbak G, Heldal M (1989). High abundance of viruses found in aquatic environments. Nature..

[13] Gregory AC, Zayed AA, Conceição-Neto N, Temperton B, Bolduc B, Alberti A (2019). Marine DNA viral macro- and microdiversity from pole to pole. Cell..

[14] Roux S, Brum JR, Dutilh BE, Sunagawa S, Duhaime MB, Loy A (2016). Ecogenomics and potential biogeochemical impacts of globally abundant ocean viruses. Nature..

[15] Gerdts G, Wichels A, Döpke H, Klings K-W, Gunkel W, Schütt C (2004). 40-year long-term study of microbial parameters near Helgoland (German Bight, North Sea): historical view and future perspectives. Helgol Mar Res.

[16] Pinhassi J, Sala MM, Havskum H, Peters F, Guadayol Ò, Malits A (2004). Changes in bacterioplankton composition under different phytoplankton regimens. Appl Environ Microbiol.

[17] Simon M, Glöckner F, Amann R (1999). Different community structure and temperature optima of heterotrophic picoplankton in various regions of the Southern Ocean. Aquat Microb Ecol.

[18] Teeling H, Fuchs BM, Becher D, Klockow C, Gardebrecht A, Bennke CM (2012). Substrate-controlled succession of marine bacterioplankton populations induced by a phytoplankton bloom. Science..

[19] Teeling H, Fuchs BM, Bennke CM, Kruger K, Chafee M, Kappelmann L (2016). Recurring patterns in bacterioplankton dynamics during coastal spring algae blooms. eLife..

[20] Gügi B, Le Costaouec T, Burel C, Lerouge P, Helbert W, Bardor M (2015). Diatom-specific oligosaccharide and polysaccharide structures help to unravel biosynthetic capabilities in diatoms. Mar Drugs.

[21] Haug A, Myklestad S (1976). Polysaccharides of marine diatoms with special reference to *Chaetoceros* species. Mar Biol.

[22] Painter TJ. Algal polysaccharides. In: Aspinall GO (eds). The polysaccharides. (Academic Press, New York, 1983) pp 195–285.

[23] Unfried F, Becker S, Robb CS, Hehemann J-H, Markert S, Heiden SE (2018). Adaptive mechanisms that provide competitive advantages to marine *Bacteroidetes* during microalgal blooms. ISME J.

[24] Reintjes G, Arnosti C, Fuchs BM, Amann R (2017). An alternative polysaccharide uptake mechanism of marine bacteria. ISME J.

[25] Thomas F, Le Duff N, Wu T-D, Cébron A, Uroz S, Riera P, et al. Isotopic tracing reveals single-cell assimilation of a macroalgal polysaccharide by a few marine Flavobacteria and Gammaproteobacteria. ISME J. 2021. e-pub ahead of print 5 May 2021. 10.1038/s41396-021-00987-x PMC8443679 33953365

[26] Gonzalez JM, Sherr EB, Sherr BF (1990). Size-selective grazing on bacteria by natural assemblages of estuarine flagellates and ciliates. Appl Environ Microbiol.

[27] Holmfeldt K, Middelboe M, Nybroe O, Riemann L (2007). Large variabilities in host strain susceptibility and phage host range govern interactions between lytic marine phages and their *Flavobacterium* hosts. Appl Environ Microbiol.

[28] Bischoff V, Bunk B, Meier-Kolthoff JP, Spröer C, Poehlein A, Dogs M (2019). Cobaviruses – a new globally distributed phage group infecting *Rhodobacteraceae* in marine ecosystems. ISME J.

[29] Chan JZM, Millard AD, Mann NH, Schäfer H (2014). Comparative genomics defines the core genome of the growing N4-like phage genus and identifies N4-like Roseophage specific genes. Front Microbiol.

[30] Wichels A, Biel SS, Gelderblom HR, Brinkhoff T, Muyzer G, Schütt C (1998). Bacteriophage diversity in the North Sea. Appl Environ Microbiol.

[31] Sabehi G, Shaulov L, Silver DH, Yanai I, Harel A, Lindell D (2012). A novel lineage of myoviruses infecting cyanobacteria is widespread in the oceans. Proc Natl Acad Sci.

[32] Suttle CA, Chan AM (1993). Marine cyanophages infecting oceanic and coastal strains of *Synechococcus*: abundance, morphology, cross-infectivity and growth characteristics. Mar Ecol Prog Ser.

[33] Wilson WH, Carr NG, Mann NH (1996). The effect of phosphate status on the kinetics of cyanophage infection in the oceanic cyanobacterium *Synechococcus* sp. WH78031. J Phycol.

[34] Fuller NJ, Wilson WH, Joint IR, Mann NH (1998). Occurrence of a sequence in marine cyanophages similar to that of T4 g20 and its application to PCR-based detection and quantification techniques. Appl Environ Microbiol.

[35] Proctor LM, Fuhrman JA (1990). Viral mortality of marine bacteria and cyanobacteria. Nature..

[36] Suttle CA, Chan AM, Cottrell MT (1990). Infection of phytoplankton by viruses and reduction of primary productivity. Nature.

[37] Holmfeldt K, Odić D, Sullivan MB, Middelboe M, Riemann L (2012). Cultivated single-stranded DNA phages that infect marine *Bacteroidetes* prove difficult to detect with DNA-binding stains. Appl Environ Microbiol.

[38] Kang I, Jang H, Cho J-C (2015). Complete genome sequences of bacteriophages P12002L and P12002S, two lytic phages that infect a marine *Polaribacter* strain. Stand Genom Sci..

[39] Borriss M, Helmke E, Hanschke R, Schweder T (2003). Isolation and characterization of marine psychrophilic phage-host systems from Arctic sea ice. Extremophiles..

[40] Jiang SC, Kellogg CA, Paul JH (1998). Characterization of marine temperate phage-host systems isolated from Mamala Bay, Oahu, Hawaii. Appl Environ Microbiol.

[41] Kang I, Kang D, Cho J-C (2012). Complete genome sequence of *Croceibacter* bacteriophage P2559S. J Virol.

[42] Kang I, Jang H, Cho J-C (2012). Complete genome sequences of two *Persicivirga* bacteriophages, P12024S and P12024L. J Virol.

[43] Sullivan MB, Huang KH, Ignacio-Espinoza JC, Berlin AM, Kelly L, Weigele PR (2010). Genomic analysis of oceanic cyanobacterial myoviruses compared with T4-like myoviruses from diverse hosts and environments. Environ Microbiol.

[44] Bankevich A, Nurk S, Antipov D, Gurevich AA, Dvorkin M, Kulikov AS (2012). SPAdes: a new genome assembly algorithm and its applications to single-cell sequencing. J Comput Biol.

[45] Mizuno CM, Ghai R, Saghaï A, López-García P, Rodriguez-Valera F (2016). Genomes of abundant and widespread viruses from the deep ocean. mBio..

[46] Mizuno CM, Rodriguez-Valera F, Kimes NE, Ghai R (2013). Expanding the marine virosphere using metagenomics. PLOS Genet.

[47] Nishimura Y, Watai H, Honda T, Mihara T, Omae K, Roux S (2017). Environmental viral genomes shed new light on virus-host interactions in the ocean. mSphere..

[48] Paez-Espino D, Roux S, Chen IMA, Palaniappan K, Ratner A, Chu K (2019). IMG/VR v.2.0: an integrated data management and analysis system for cultivated and environmental viral genomes. Nucleic Acids Res.

[49] Labonté JM, Swan BK, Poulos B, Luo H, Koren S, Hallam SJ (2015). Single-cell genomics-based analysis of virus–host interactions in marine surface bacterioplankton. ISME J.

[50] Martinez-Hernandez F, Fornas O, Lluesma Gomez M, Bolduc B, de la Cruz Peña MJ, Martínez JM (2017). Single-virus genomics reveals hidden cosmopolitan and abundant viruses. Nat Commun.

[51] Moraru C. VirClust, a tool for hierarchical clustering, core gene detection and annotation of (prokaryotic) viruses. bioRxiv. 2021:2021.06.14.448304. 10.3390/v15041007 PMC10143988 37112988

[52] Nishimura Y, Yoshida T, Kuronishi M, Uehara H, Ogata H, Goto S (2017). ViPTree: the viral proteomic tree server. Bioinformatics..

[53] Meier-Kolthoff JP, Göker M (2017). VICTOR: genome-based phylogeny and classification of prokaryotic viruses. Bioinformatics..

[54] Aiewsakun P, Adriaenssens EM, Lavigne R, Kropinski AM, Simmonds P (2018). Evaluation of the genomic diversity of viruses infecting bacteria, archaea and eukaryotes using a common bioinformatic platform: steps towards a unified taxonomy. J Gen Virol.

[55] Moraru C, Varsani A, Kropinski AM (2020). VIRIDIC-A novel tool to calculate the intergenomic similarities of prokaryote-infecting viruses. Viruses..

[56] Edgar RC (2004). MUSCLE: multiple sequence alignment with high accuracy and high throughput. Nucleic Acids Res.

[57] Nguyen L-T, Schmidt HA, von Haeseler A, Minh BQ (2014). IQ-TREE: a fast and effective stochastic algorithm for estimating maximum-likelihood phylogenies. Mol Biol Evolut.

[58] Hoang DT, Chernomor O, von Haeseler A, Minh BQ, Vinh LS (2017). UFBoot2: improving the ultrafast bootstrap approximation. Mol Biol Evolut.

[59] Kalyaanamoorthy S, Minh BQ, Wong TKF, von Haeseler A, Jermiin LS (2017). ModelFinder: fast model selection for accurate phylogenetic estimates. Nat Methods.

[60] Rambaut A. 1.4.4—a graphical viewer of phylogenetic trees and a program for producing publication-ready figures. 2018.

[61] Camacho C, Coulouris G, Avagyan V, Ma N, Papadopoulos J, Bealer K (2009). BLAST+: architecture and applications. BMC Bioinform.

[62] Galiez C, Siebert M, Enault F, Vincent J, Söding J (2017). WIsH: who is the host? Predicting prokaryotic hosts from metagenomic phage contigs. Bioinformatics..

[63] Nayfach S, Roux S, Seshadri R, Udwary D, Varghese N, Schulz F (2020). A genomic catalog of Earth’s microbiomes. Nat Biotechnol.

[64] Rodriguez-R LM, Gunturu S, Harvey WT, Rosselló-Mora R, Tiedje JM, Cole JR (2018). The Microbial Genomes Atlas (MiGA) webserver: taxonomic and gene diversity analysis of Archaea and Bacteria at the whole genome level. Nucleic Acids Res.

[65] Stamatakis A (2014). RAxML version 8: a tool for phylogenetic analysis and post-analysis of large phylogenies. Bioinformatics..

[66] Ludwig W, Strunk O, Westram R, Richter L, Meier H, Yadhukumar (2004). ARB: a software environment for sequence data. Nucleic Acids Res.

[67] Peplies J, Kottmann R, Ludwig W, Glöckner FO (2008). A standard operating procedure for phylogenetic inference (SOPPI) using (rRNA) marker genes. Syst Appl Microbiol.

[68] Couvin D, Bernheim A, Toffano-Nioche C, Touchon M, Michalik J, Néron B (2018). CRISPRCasFinder, an update of CRISRFinder, includes a portable version, enhanced performance and integrates search for Cas proteins. Nucleic Acids Res.

[69] Forterre P, Soler N, Krupovic M, Marguet E, Ackermann H-W (2013). Fake virus particles generated by fluorescence microscopy. Trends Microbiol.

[70] Nagasaki K (2008). Dinoflagellates, diatoms, and their viruses. J Microbiol.

[71] Koonin EV, Dolja VV, Krupovic M, Varsani A, Wolf YI, Yutin N (2020). Global organization and proposed megataxonomy of the virus world. Microbiol Mol Biol Rev.

[72] Barylski J, Enault F, Dutilh BE, Schuller MB, Edwards RA, Gillis A (2019). Analysis of Spounaviruses as a case study for the overdue reclassification of tailed phages. Syst Biol.

[73] Turner D, Kropinski AM, Adriaenssens EM (2021). A roadmap for genome-based phage taxonomy. Viruses..

[74] Taylor AL (1963). Bacteriophage-induced mutation in *Escherichia coli*. Proc Natl Acad Sci USA..

[75] Casjens SR, Gilcrease EB. Determining DNA packaging strategy by analysis of the termini of the chromosomes in tailed-bacteriophage virions. In: Clokie MRJ, Kropinski AM (eds). Bacteriophages: methods and protocols, Volume 2 Molecular and Applied Aspects. (Humana Press, Totowa, NJ, 2009) pp 91–111. 10.1007/978-1-60327-565-1_7 PMC3082370 19082553

[76] Montaño SP, Pigli YZ, Rice PA (2012). The Mu transpososome structure sheds light on DDE recombinase evolution. Nature..

[77] Krupovic M (2013). Networks of evolutionary interactions underlying the polyphyletic origin of ssDNA viruses. Curr Opin Virol.

[78] Wohlkönig A, Huet J, Looze Y, Wintjens R (2010). Structural relationships in the lysozyme superfamily: significant evidence for glycoside hydrolase signature motifs. PLoS One.

[79] Dziewit L, Oscik K, Bartosik D, Radlinska M (2014). Molecular characterization of a novel temperate *Sinorhizobium* bacteriophage, ФLM21, encoding DNA methyltransferase with CcrM-like specificity. J Virol.

[80] Souza CP, Almeida BC, Colwell RR, Rivera ING (2011). The importance of chitin in the marine environment. Mar Biotechnol.

[81] Desikachary TV, Dweltz NE (1961). The chemical composition of the diatom frustule. Proc Indian Acad Sci—Sect B.

[82] Khotimchenko Y, Khozhaenko E, Kovalev V, Khotimchenko M (2012). Cerium binding activity of pectins isolated from the seagrasses *Zostera marina* and *Phyllospadix iwatensis*. Mar Drugs.

[83] Hay ID, Ur Rehman Z, Moradali MF, Wang Y, Rehm BHA (2013). Microbial alginate production, modification and its applications. Microb Biotechnol.

[84] Krüger K, Chafee M, Francis TB, Glavina del Rio T, Becher D, Schweder T (2019). In marine *Bacteroidetes* the bulk of glycan degradation during algae blooms is mediated by few clades using a restricted set of genes. ISME J.

[85] Avcı B, Krüger K, Fuchs BM, Teeling H, Amann RI (2020). Polysaccharide niche partitioning of distinct *Polaribacter* clades during North Sea spring algal blooms. ISME J.

[86] Kappelmann L, Krüger K, Hehemann J-H, Harder J, Markert S, Unfried F (2019). Polysaccharide utilization loci of North Sea *Flavobacteriia* as basis for using SusC/D-protein expression for predicting major phytoplankton glycans. ISME J.

[87] Maleki S, Almaas E, Zotchev S, Valla S, Ertesvåg H (2016). Alginate biosynthesis factories in *Pseudomonas fluorescens*: localization and correlation with alginate production level. Appl Environ Microbiol.

[88] Singh JK, Adams FG, Brown MH (2019). Diversity and function of capsular polysaccharide in Acinetobacter baumannii. Front Microbiol.

[89] Limoli DH, Jones CJ, Wozniak DJ. Bacterial extracellular polysaccharides in biofilm formation and function. Microbiol Spect. 2015;3. 10.1128/microbiolspec.MB-0011-2014 PMC4657554 26185074

[90] Latka A, Maciejewska B, Majkowska-Skrobek G, Briers Y, Drulis-Kawa Z (2017). Bacteriophage-encoded virion-associated enzymes to overcome the carbohydrate barriers during the infection process. Appl Microbiol Biotechnol.

[91] Pires DP, Oliveira H, Melo LDR, Sillankorva S, Azeredo J (2016). Bacteriophage-encoded depolymerases: their diversity and biotechnological applications. Appl Microbiol Biotechnol.

[92] Bižić-Ionescu M, Zeder M, Ionescu D, Orlić S, Fuchs BM, Grossart H-P (2015). Comparison of bacterial communities on limnic versus coastal marine particles reveals profound differences in colonization. Environ Microbiol.

[93] Silpe JE, Bassler BL (2019). Phage-encoded LuxR-type receptors responsive to host-produced bacterial quorum-sensing autoinducers. mBio..

[94] Silpe JE, Bassler BL (2019). A host-produced quorum-sensing autoinducer controls a phage lysis-lysogeny decision. Cell..

[95] Bowman JP. The marine clade of the family *Flavobacteriaceae*: the genera *Aequorivita, Arenibacter, Cellulophaga, Croceibacter, Formosa, Gelidibacter, Gillisia, Maribacter, Mesonia, Muricauda, Polaribacter, Psychroflexus, Psychroserpens, Robiginitalea, Salegentibacter, Tenacibaculum, Ulvibacter, Vitellibacter and Zobellia*. In: Dworkin M, Falkow S, Rosenberg E, Schleifer K-H, Stackebrandt E (eds). The Prokaryotes: Volume 7: Proteobacteria: Delta, Epsilon Subclass. (Springer New York, New York, NY, 2006) pp 677–94.

[96] Needham DM, Chow C-ET, Cram JA, Sachdeva R, Parada A, Fuhrman JA (2013). Short-term observations of marine bacterial and viral communities: patterns, connections and resilience. ISME J.

[97] Ignacio-Espinoza JC, Ahlgren NA, Fuhrman JA (2020). Long-term stability and Red Queen-like strain dynamics in marine viruses. Nat Microbiol..

[98] Wiltshire KH, Kraberg A, Bartsch I, Boersma M, Franke H-D, Freund J (2010). Helgoland Roads, North Sea: 45 years of change. Estuaries Coasts.

[99] Shimodaira H, Terada Y (2019). Selective inference for testing trees and edges in phylogenetics. Front Ecol Evolut.

[100] Suzuki R, Shimodaira H (2006). Pvclust: an R package for assessing the uncertainty in hierarchical clustering. Bioinformatics..

