# Supplementary material for: Highly diverse flavobacterial phages isolated from North Sea spring blooms
Source: ISME J. 2021 Sep 2;16(2):555–68. doi: 10.1038/s41396-021-01097-4 (PMC8776804; doi:10.1038/s41396-021-01097-4)
Supplement: Supplementary file 1 — SI_File_1 [file 41396_2021_1097_MOESM1_ESM.pdf]

Supplementary Information_file_1

Materials and Methods

Environmental samples

Chlorophyll measurements

Chlorophyll *a,* green algae, and diatoms were measured via fluorescence using an algal group analyser (bbe moldaenke, Kiel-Kronshagen, Germany).

Total bacterial counts and *Bacteroidetes* counts

Samples for total cell counts were fixed with a final concentration of 1% formaldehyde for 1 h at room temperature. Aliquots of 10 ml were filtered on 0.2 µm polycarbonate filters (Merck Millipore, Burlington, USA), and stained with 4',6-diamidino-2-phenylindole (DAPI, 1µg/ml) for 20 min at room temperature and shortly washed with MilliQ water and 80% ethanol. Dried filters were automatically counted with the Zeiss Axio Imager.Z2 (Carl Zeiss MicroImaging GmbH, Jena, Germany) and quantified with the modified ACMEtool3.0 (Zeder, M. 2005-2010, Software for Biology, https://www.mpi-bremen.de/en/automated-microscopy.html) after (1).

On the same filter, catalysed reporter deposition – fluorescence *in situ* hybridization (CARD-FISH) was performed with the CF319a probe (TGGTCCGTGTCTCAGTAC) (2) according to (3) with modifications. Hybridization was done with horseradish peroxidase (HRP)-labeled oligonucleotide probes at 35% formamide concentration.

Determination of virus-like particle (VLP) numbers using epifluorescence microscopy

Epifluorescence microscopy of fluorescently stained samples was used to count virus like particles (VLPs). For this purpose 0.5 ml of the of 0.2 µm filtrate were filtered through a 0.02 µm pore size Anodisc filter (GE Healthcare Life Sciences, Maidstone, UK), and stained with SYBR Gold (Invitrogen, Carlsbad, USA) (25x final concentration) for 14 min at room temperature. At least 10 fields of view were counted (4, 5) using the Zeiss AxioImager.D2 (Carl Zeiss MicroImaging GmbH).

Determination of phage numbers using transmission electron microscopy (TEM)

Phages were also counted by transmission electron microscopy (TEM) with the JEM-2100 (JEOL, Tokyo, Japan). Samples were filtered through a 3 µm pore-sized filter to remove big aggregates, fixed with a final concentration of 2% glutaraldehyde, and stored at 4°C. For electron microscope grid preparation, 10 ml were centrifuged in a SW40TI (Beckman Coulter, Brea, USA) at 20,000 rpm for 1 hour at 4°C on two carbon coated copper grids with a polyvinyl butyral support film. Samples were negative stained for 1 min with 0.5% uranyl acetate and images of 2-3 meshes were taken, with a TemCam F416 (TVIPS, Gauting, Germany) camera, a beam of 44 µA and 80 kV, and 12,000x magnification. A detailed description of the method can be found in (6).

Phage isolation

Media preparation

2216 Difco Marine Broth (MB)

Medium was prepared following the manufacturer's recommendation. In short, 37.4 g Difco Marine Broth (BD Biosciences, San Jose, CA, USA) were dissolved in 1 l MilliQ water and autoclaved at 121°C for 20 min. This medium was used in 2017.

Marine Broth (MB) from native sea water

North Sea surface water was filtered through a 0.2 µm pore-sized membrane (Merck Millipore) diluted with MilliQ water to a final concentration of 75%. Both, 1 g l^{-1} Bacto Yeast Extract (BD Biosciences) and 5 g l^{-1} Bacto Tryptone (BD Biosciences) were dissolved, the pH was adjusted to 7.6, and the medium was autoclaved at 121°C for 20 min. This medium was used in 2018, because the bacteria grew better in this medium.

Agar for Plaque Assay and Spot Assay

For the bottom agar, 15 g l^{-1} Bacto Agar (BD Biosciences) was added to the above described media before autoclaving, but after pH adjustment. 10 ml were poured in a 92 mm vented Petri dish (Sarstedt) and dried for one day at room temperature. Plates were stored at 4°C and again dried for at least one hour before usage.

For the top agar, 6 g l^{-1} Bacto Agar (BD Biosciences) was added to the above described media before autoclaving, but after pH adjustment. 3 ml were sterile poured into 15 ml tubes (Sarstedt). Tubes were stored at 4°C and melted in boiling water prior usage. In order to avoid killing the bacterium it was cooled down to 42°C in a water bath.

Spot Assay

After transferring 500 µl of densely grown host culture to the molten top agar (42°C), the mixture was poured on top of the bottom agar and spread by swirling. After solidification 10 µl of phage stock was transferred to the upper third of the plate. If several samples were tested on a single plate, those drops were in a horizontal line. Plates were tilted in order to start a slow flow of the drops which was stopped before a drop was reaching the rim of the Petri dish. Plates were incubated at 18°C for one to two days, depending on the host growth.

Plaque Assay

To obtain single plaques, a decadal dilution series of the phage stock was performed with saline magnesium (SM) buffer (5.8 g l^{-1} NaCl, 2.0 g l^{-1} $MgSO_4 * 7 H_2O$, and 50 mM Tris-HCl (pH 7.5), dissolved in MilliQ water, and autoclaved). For each dilution a single plate was used following the same procedure: 100 µl of phage liquid was transferred to the middle of the bottom agar plate. 500 µl of densely grown host culture was transferred to molten top agar. This mixture was poured on the bottom agar and spread to cover the whole plate. After solidification, plates were incubated at 18°C for one to two days, depending on the host growth.

Phage isolation from enrichments and direct-plating

Phages were enriched by mixing 740 ml seawater consecutively pre-filtered through 10 µm, 3 µm, and 0.2 µm pore-size polycarbonate filters (Merck Millipore, Burlington, USA) in 1 l glass bottles (Schott AG, Mainz, Germany) with 27 ml 10x MB medium (7) in a final concentration of 0.3 x 2216 marine broth medium (see above). To this mix 27 ml of flavobacterial culture was added pre-grown at 18°C to logarithmic phase in MB medium. The enrichments were incubated for four days at 18°C with 100 rpm shaking. In total, 23 bacterial strains were tested (6 in 2017, 21 in 2018) (Tab.1). Afterwards, the enriched phage fraction was obtained by filtering 10 ml through a 0.2 µm syringe filter (Merck Millipore). Successful enrichments were detected by a spot test (8) with the host used for enrichment. If clearing zones were obtained, indicating phage lysis, phage dilutions were plated. Three plaques each were picked and used as inoculum for new plaque assays. This procedure was repeated three times before a phage stock was prepared.

Phage stock preparation

At least three single plaques were picked from the plates of the plaque assay with a 1 µl plastic inoculation tube. Those plaques were dissolved in modified SM buffer, diluted, and plated again. This procedure was repeated three times to ensure the purity of the final phage stock. With a fourth dilution series the stock was prepared by adding 10 ml SM buffer to a plate with confluent lysis (9). After 1 h at room temperature, the liquid was filtered through a 0.2 µm syringe filter (Merck Millipore) and stored in glass tubes at 4°C.

Host range determination

For host range determination all bacterial isolates used for the enrichment cultures were tested with each obtained phage group. An agar overlay was performed and 10 µl of phage stock were spotted on the plate (see spot test). Four to five stocks were tested per plate. If lysis was observed an additional overlay was done with only one phage stock per plate. Lysis showing combinations were tested again with a plaque assay. In addition, the hosts were checked for purity by plating and 16S rRNA sequencing.

Determination of phage morphology using TEM

Phage stocks obtained from lysates were fixed with glutaraldehyde at a final concentration of 0.5% (2.5% glutaraldehyde, 2.5 mM $MgCl_2$, 50 mM KCl, 50 mM cacodylic acid, pH 7.2). Approximately 10 µl of fixed phages were transferred onto glow discharged carbon coated copper grids. After 2 min the solution was removed from the grids with filter paper, grids were washed for 10 sec with deionized H_2O and then negative stained with 0.5% uranyl acetate for 1 min. The samples were visualized with a Zeiss EM900 TEM (Zeiss, Oberkochen, Germany). TEM negatives were digitalized with an Epson Perfection V700 Photo scanner.

Determination of phage genomes

Phage DNA extraction

First, free nucleic acids were digested with DNase 1 (Ambion, 0.004 U/µl) and RNase 1 (Ambion, 0.1 U/µl). Then, the capsid was opened with Proteinase K (0.05 U/ml) and SDS (0.5%). Finally, the phage DNA was loaded on Wizard columns (Promega, Madison, USA) by mixing 1 ml of phage lysate with the Wizard resin. A vacuum manifold was used for the extraction. DNA was eluted in TE Buffer (after (10)).

Phage genome sequencing

Both types of phages were sequenced with the Illumina HiSeq3000 (paired-end read 2 x 150 bp). For potentially ssDNA phages and *Maribacter* phages a ChIPSeq library was prepared with the DNA SMART ChIPSeq Kit (Takara Bio Europe S.A., Saint-Germain-en-Laye, France) and 12-18 PCR cycles. For dsDNA phages a DNA FS library was prepared using the DNA Ultra II kit (New England Biolabs GmbH, Frankfurt am Main, Germany) with fragmentation by S2 tubes (Covaris, Woburn, MA, USA) or with chemical fragmentation for 9 min and 3-12 PCR cycles.

Phage genome assembly

The raw reads were quality trimmed with BBDuk (v35.14, sourceforge.net/projects/bbmap/), using the parameters “qtrim=rl trimq=20, maq=20 minlen=30 ordered t=8". The cleaned reads were then assembled both with SPAdes (v3.13.0, (11)) and Tadpole (v35.14, sourceforge.net/projects/bbmap/). The parameters for Tadpole were "k=50 t=8". For the first batch of genomes the parameters for SPAdes were "-k 35,55,75 --sc". In a second batch the parameters for SPAdes were “-k 31, 41, 51, 61, 71, 81, 91, 101, 111, 121, 127 -m 500”. If more than one contig was obtained, a normalization of the reads with BBNorm (v35.14, sourceforge.net/projects/bbmap/) was performed with the parameters "target=100 min=5", followed by an assembly as above. BBDuk, Tadpole and BBNorm are part of the BBTools package (https://jgi.doe.gov/data-and-tools/bbtools/).

Bandage (12) was used to assess the quality of the assembly. If the phage genome had several nodes, or if the genome was linear, primers were designed and amplified fragments were sequenced using the Sanger technology (see below). In order to validate the genome size of the assembly, Pulsed Field Gel Electrophoresis was performed with agarose plugs containing phage stock solution (see below).

Determination of phage genome ends

Gap closure of phage genomes

Primers were designed with SnapGene (GSL Biotech LLC) around the missing/questionable region. The amplified DNA was Sanger-sequenced with a 3130xl Genetic Analyzer (ABI PRISM), manually trimmed with FinchTV (v1.4.0, Geospiza Inc.) and mapped on the genome with the Geneious mapper with standard parameters.

Determination of phage genome size by Pulsed Field Gel Electrophoresis (PFGE)

For plug preparation 50 µl of 2% low melting agarose (invitrogen, UltraPure™ LMP Agarose) were mixed with 50 µl of phage stock solution and transferred into a plug mold (BIO-RAD, CHEF® Mapper XA System). Plugs were incubated in modified SM buffer with sodium dodecyl sulphate (SDS) (0.5%) and Proteinase K (Macherey-Nagel, 0.26 U/ml) at 56°C for 24 hours. Plugs were washed two times in TE Buffer (10 mM Tris-HCl pH 8, 0.1 mM EDTA) at room temperature for 24 hours. Samples were run in a 1% Pulsed Field Gel (BIO-RAD, Pulsed Field Certified Agarose) in 0.5 x TBE Buffer (0.045 M Tris, 0.045 Boric Acid, 0.001M EDTA) with a CHEF-DR® III System and cooling module (BIO-RAD). The initial switch time was 0.3 sec and was increased by a linear ramp to 11.5 sec. The run time was 19.5 hours with 6 V/cm, an included angle of 120°, in 0.5 x TBE buffer in a 14°C cooled system. Gels were with ethidium bromide and images were taken with an Intas UV-System Gel iX Box (13).

Phage genome enzymatic digestion

DNA of Omtje_3 was extracted with the Wizard DNA extraction resin (1,08 µg/µl), and digested at room temperature for 40 min with the Exonuclease III (Thermo Fisher, final concentration 25 U/µl) and DNase I (Ambion, final concentration 0.5 U/µl). Digestion products were analysed with a fragment analyzer system 5200 (Agilent Technologies, Santa Clara, CA, USA) with the HS Genomic DNA Kit (Agilent Technologies).

Retrieval of related phage genomes from public sequence databases

ICTV recognized dsDNA phages

As reference for the ICTV dataset, the Master Species List 2020.v1 (March 2021, https://talk.ictvonline.org/files/master-species-lists/m/msl/12314). Therefore, the *Caudovirales* dataset consisted of eleven ICTV recognized families: *Herelleviridae*, *Demerecviridae*, *Autographiviridae*, *Ackermannviridae*, *Drexlerviridae*, *Chaseviridae*, *Salasmaviridae*, *Rountreeviridae*, *Schitoviridae*, *Zobellviridae*, *Guelinviridae*. *Myoviridae, Podoviridae* and *Siphoviridae* were excluded, because phages were assigned to these families based on their morphology and most likely, these families will be dissolved in time.

Retrieval of phage genomes related to dsDNA flavophages from GenBank Viral

All viral genomes from the following dsDNA viral taxa were downloaded on 17.03.2021: phylum *Uroviricota,* and families *Corticoviridae*, *Tectiviridae*, *Autolykiviridae*, *Plasmaviridae, Ampullaviridae, Bicaudaviridae, Fuselloviridae, Globuloviridae, Guttaviridae, Lipothrixviridae, Rudiviridae* and *Halspiviridae*. These viral genomes were compressed in a single file, called from here on gbViral_DB. To find relatives for our isolated flavophages, the flavophage genomes were used as query, and the gbViral_DB as search database. For both query and gbViral genomes, ORFs were predicted using MetaGeneAnnotator (14) and translated into proteins with a custom R script using the seqinr package and the translation code 11. The gbViral proteins were pooled into a single BLAST database, named here gbViral_prot_DB, using makeblastdb tool from the BLAST+ 2.6.0 package (15). Then, each flavophage protein was used as query in a BLASTP search ("-evalue 0.01 -max_target_seqs 'number of DB proteins'") against gbViral_prot_DB. A gbViral_DB genome was considered a flavophage relative if: i) it had in common with a query flavophage genome at least 5% of its proteins (bitscore > 50); and ii) it had a genome size not smaller than 50% and not bigger than 150% compared with the respective flavophage genome.

Retrieval of phage genomes related to dsDNA from environmental datasets

First, the environmental datasets (16-22) were prepared. For this, contigs smaller than 10 kb were removed. Open reading frames (ORFs) were predicted with MetaGeneAnnotator (14). The ORFs were then translated into proteins with a custom R script using the seqinr package and the translation code 11. All predicted proteins were pooled into a single BLAST database, named here ENV_DB, using makeblastdb tool from the BLAST+ 2.6.0 package (15). Second, flavophage related contigs were found as follows. The proteins of the isolated flavobacterial phages were used as query for a BLASTP search against ENV_DB, with the parameters "-evalue 0.001 -max_target_seqs 10000". Protein hits with a bitscore lower than 50 were removed. All contigs from ENV_DB with at least four protein hits with a flavophage query were selected. And third, all flavophage genomes and the above selected environmental genomes were clustered using vConTact2 (23, 24) with the standard parameters and cultivated phages of the

ProkaryoticViralRefSeq85-ICTV. All environmental phage contigs and the reference genomes that formed one cluster with the flavophage isolates were selected for further analysis. Only environmental contigs with a length >80% compared to their related flavophage isolate were kept.

Retrieval of phage genomes related to ssDNA flavophages from NCBI Virus

All our ssDNA flavophage genomes and their proteins were used as query in a BLASTN / BLASTP search against the NT/NR database from NCBI (https://blast.ncbi.nlm.nih.gov/Blast.cgi, (15)). The phage genomes corresponding to the BLAST results and to further reference *Monodnaviria* phages were downloaded from NCBI Virus (https://www.ncbi.nlm.nih.gov/labs/virus/vssi/#/). The official taxonomy of the reference phages was collected from the ICTV database (the Master Species List 2020.v1 (March 2021, https://talk.ictvonline.org/files/master-species-lists/m/msl/12314).

Phage genome annotation

First, the ORFs were predicted using MetaGeneAnnotator and translated using a custom R script, with the seqinr package and translation code 11. Second, tRNAs and tmRNAs where predicted using tRNAscan 2.0 (parameters “-q -B –D”) (25, 26) and Aragorn v1.2.38 (parameters “-m -fo - gcbact –fon”) (27), integrated in a custom R script. The InterProScan (28) plugin from Geneious v 11.1.5 (http://www.geneious.com, (29) was used to predict the cellular localization of the protein domains and presence of signal peptides.

Isolation and cultivation of particle-associated heterotrophic bacterial strains

Bacterial strains were isolated in spring 2017 from particle-enriched surface seawater samples on 2216 plates and on an artificial seawater plate medium with 2 g L^{-1} laminarin as major carbon source (modified from (30)). They were incubated at 12°C in the dark. Further details are shown in Table 17.

Host imaging by Scanning Electron Microscopy (SEM)

Bacterial colonies grown on agar plates were excised together with the underlying agar and fixed for 12-24 hours at 4°C with 6.25% glutaraldehyde (Merck, Darmstadt, Germany), 50 mM Soerensen phosphate buffer, pH 7.4. Samples were then washed three times with 66 mM Soerensen phosphate buffer, pH 7.4. Samples were stepwise dehydrated with acetone, critical point dried (critical point dryer: BAL-TEC CPD 030) and metal coated (sputter coater BAL-TEC SCD 005) with gold-palladium. Specimens were inspected with a field emission scanning electron microscope (JEOL JSM-7500F) at 5 kV using a detector for secondary electrons (LEI detector).

Sequence analysis of host 16S rRNA genes

Host strains were grown on 2216 medium plates. Single colonies were picked and transferred to 20 µl PCR grade water. After three freeze-thaw cycles a PCR was performed with GM3_F and GM4_R as primers to amplify the 16S rRNA gene. Products were purified and the sequencing reaction with the primers GM3_4, GM4_R, GM1_F, and GM1_R (31) was performed. After purification, the products were sequenced with a Sanger machine 3130xl Genetic Analyzer (ABI PRISM), manually trimmed with FinchTV (v1.4.0, Geospiza Inc.), and assembled with Geneious (29).

Host genome sequencing

Isolates from phage – host systems were additionally genome sequenced with a Sequel I (Pacific Biosciences, Menlo Park, USA) using 16mer barcodes. The library was prepared according to 20 kb template preparation for Sequel Systems using the SMRT bell Template Prep Kit 1.0 SPv3 (Pacific Biosciences). After using the Covaris g-tube fragmentation for 9 kb fragments (Woburn, USA), a size selection on a Blue Pippin (Sage Science, Beverly, USA) was done to enrich for fragments above 8 kb in a 0.75% cassette.

Host genome assembly and analysis

Reads were assembled with the HGAP4 assembler (32) implemented in PacBio SMRT Link v 6 with standard parameters or the CANU assembler v 1.8 (33) with circular consensus sequences (CCS), pacbio-corrected, and a genome size of 5Mb. The CCS were generated with SMRT Link and a minimum predicted accuracy of 0.9 and minimum number of passes of 3. Assemblies were manually edited to remove duplicated overlapping regions. The 16S rRNA genes were retrieved using the MiGA online platform (34). The average nucleotide identity (ANI) was calculated with the enveomics command line package (35).

Polaribacter Phages

“Freyavirus Freya" species

The “Freyavirus Freya” was isolated very early in the bloom with its host *Polaribacter* sp. HaHaR_3_91 (DSM111048). The ten strains obtained have a nucleotide identity of 94.68-99.05%. All genomes are circular, ranging from 43,978 up to 48,920 bp and had a GC content of 28.9%. The diameter of the capsid was 53.9 ± 4.7 nm, the tail was 151.0 ± 8.2 nm long, and 13.1 ± 2.0 nm wide. The morphology was siphoviral-like. Genomes varied in size by 11 genes, which were all annotated as hypothetical proteins. Genes encoding capsid, tail, tape measure, terminase, and portal proteins were annotated. Closely related sequences are also from the Norwegian Sea and are 16.2% and 13.7% similar.

“Freyavirus Danklef” species

“Freyavirus Danklef” with its five strains Danklef_1-5 (identity > 95.6%) were isolated late in the 2018 bloom, infecting *Polaribacter* sp. R2A056_3_33 (DSM111047). The capsid of Danklef was 46.1 ± 2.2 nm in diameter, the tail was 157.4 ± 4.6 nm long, and 12.1 ± 1.8 nm wide. Danklef had a siphoviral morphology. Its circular genome had a size of 47,177-47,426 bp with a GC content of 28.9%. Danklef’s major capsid protein was HK97-like. It had genes coding for a portal, tape measure, terminase, integrase, and N-acetylmuramidase protein. Interestingly, Danklef had a ferric uptake regulator family related gene. Danklef’s closest environmental relatives are coming from the Norwegian Sea and are 16.8 and 15.14% similar.

Freya and Danklef were closely related (71.38%) and belong to the same genus. Comparing the two phages, Freya had a peptidase and a DNA replication protein, whereas Danklef had two endonucleases, a methylase, and an endolysin, which were not found in Freya.

“Leefvirus Leef” species

“Leefvirus Leef” infected *Polaribacter* sp. AHE13PA (DSM111061). Leef was isolated at the peak of the bacterial bloom. It had a capsid size of 49.2 ± 3.6 nm, a tail length of 138.7 ± 9.6 nm, and tail width of 11.1 ± 2.0 nm. The appearance was siphoviral. Its circular genome had Cos 3’ ends, a size of 37,547 bp and a GC content of 29.7%. Leef encoded proteins related to the HK97 Phage, a LuxR, a BACON (Bacteroidetes-Associated Carbohydrate-binding Often N-terminal) domain, a pectin lyase, and two integrases. Genes encoding capsid, tail tape measure, and neck proteins were annotated along with a terminase and a portal protein. An N-acetylmuramidase was detected in Leef, surrounded by transmembrane domains (TMDs) containing proteins. The closest relative to Leef was node 1833 from the GOV2 dataset, which was sampled in the Barents Sea. They were 30.4% similar.

Cellulophaga Phages

“Omtjevirus Omtje” species

With *Cellulophaga* sp. HaHaR_3_176 (DSM111152) a set of five closely related phages were isolated, to which we refer as Omtje_1-5 (sequence identity > 99.9%). They are all belonging to the species “Omtjevirus Omtje”. Only Omtje_3 was isolated in 2017 and 2018. Omtje had a capsid diameter of 52.3 ± 4.6 nm, and lacked a tail. Thin sections of the phages show a potential lipid layer inside the capsid. DNA digestion revealed that they are ssDNA viruses. Their small circular genome of 6,558 bp with 31.2% GC content also suggests that they belong to the tail-less ssDNA phages. ORF prediction revealed 13 genes, which mostly encoded for structural proteins. In addition, a replication initiation factor and a lysis protein (N-aceylmuramoyl-L-alanine-amidase) were identified. Omtje were 57.6% similar to the Cellulophaga phage phi12:2 (NC_021797.1), which is a ssDNA phage isolated from the Baltic Sea in 2000 (36, 37).

“Ingelinevirus Ingeline” species

The “Ingelinevirus Ingeline” infected *Cellulophaga* sp. HaHaR_3_176 (DSM111152). This phage group is very diverse, as indicated by eight isolates, Ingeline 1-8, with high nucleotide identity above 99.9%. Ingeline_7 and Ingeline_8 were isolated in 2017 and 2018. Ingeline had a capsid diameter of 59.0 ± 5.3 nm and a tail, which was 132.9 ± 19.3 nm long, and 11.2 ± 1.7 nm wide. It had a circular genome ranging between 42,624 and 42,797 bp and a GC content of 32.2%. We annotated genes encoding a capsid, tail, tail tape measure, adaptor, portal, and a potential spanin protein. The morphology observed by TEM was that of a siphovirus. Interestingly, the genome also encodes a LuxR gene and a BACON domain-containing protein. Its closest environmental relative is Ga0105354_1000171 from the Norwegian Sea with 12.4% identity. Although Ingeline is lytic to its original *Cellulophaga* host, it contains two integrases, indicating the potential for lysogeny.

“Callevirus Calle” species

With *Cellulophaga* sp. HaHa_2_95 (DSM111037) the podoviral “Callevirus Calle” was isolated. Three strains Calle_1-3, which were 99.95-99.98% similar, belong to this species. This species was present throughout the 2018 phytoplankton bloom. The capsid had a diameter of 60.3 ± 3.0 nm. The tail was 23.0 ± 5.5 nm long, and 13.5 ± 2.4 nm wide. The circular genome length of the strains ranged from 72,979

to 72,980 bp and the GC content was 38.1%. The genome encoded a capsid, tail, and DNA polymerase protein. In addition, it contained 20 tRNAs and a tRNA-splicing ligase RtcB. This phage also encoded a tmRNA. Both types of RNA are suggesting a more efficient phage replication and might increase the host range. Indeed, Calle is able to infect another *Cellulophaga* strain (HaHa_2_1). Two chaperonin proteins, which are associated with the GroEL system, were also encoded in the genome. Its closest relative was the Cellulophaga phage phi38:1 (KC821614.1) with 92.2% identity, which was isolated in the Baltic Sea in 2005 (36) with a closely related host (99.5% 16S rRNA sequence identity and 94.2% ANI). Phi38:1 and Calle belong to an abundant cluster of marine phages (38).

“Nekkelsvirus Nekkels” species

The “Nekkelsvirus Nekkels” was isolated by direct plating of sea surface water with the host *Cellulophaga* sp. HaHa_2_1 (DSM111038). Spot tests indicated that this phage group is present during most of the sampling period. The second strain, Nekkels_2, had a nucleotide identity of 97.1%. Nekkels had a capsid diameter of 54.8 ± 5.0 nm, its tail was 141.0 ± 7.9 nm long, and 13.0 ± 1.5 nm wide. The two genomes varied in length. Nekkels_2 encoded in its 54,332 bp genome two ORFs more than Nekkels_1 (53,385 bp). Both circular genomes had a 31.5% GC content. The genomes encoded a major capsid, tail tape measure, neck, terminase, pectate lyase, a lysozyme (GH19), and a potential spanin protein. Additional genes were the acyl carrier protein and a Yersinia outer protein X (YopX). Although Nekkels had a 40.5% nucleotide identity with the *Cellulophaga phage phi19:1* (KC821607.1). Nekkels was able to infect another flavobacterial host: AHE13PA, a *Polaribacter* sp., but with a lower efficiency.

Other flavophages

“Harrekavirus Harreka” species

The “Harrekavirus Harreka” was isolated during the bacterial bloom in 2018 with the host *Olleya* sp. HaHaR_3_96 (DSM111044). The capsid diameter was 44.3 ± 3.6 nm, the tail was 123.8 ± 8.0 nm long and 14.3 ± 2.0 nm wide. Harreka had a myoviral morphology. In total seven stocks were obtained and all of them revealed the 100% identical 43,175 bp circular genome with a GC content of 32%. Genes for capsid, tail, portal, replication proteins, GH19 and YopX were identified. Harreka was able to infect the two closely related *Tenacibaculum* strains AHE14PA and AHE15PA.

“Peternellavirus Peternella” species

From *Winogradskyella* sp. HaHa_3_26 (DSM111041) “Peternellavirus Peternella” was isolated once in 2018 during the bacterial bloom. It has a capsid diameter of 52.3 ± 4.2 nm, a tail length of 105.8 ± 7.4 nm, and a tail width of 16.4 ± 2.4 nm. The morphology was myoviral. From four phage stocks, the same 39,649 bp long linear genome with 35.3% GC content was retrieved. This phage belongs to the Mu-like phages, due to its overlapping reads with the host genome and the typical genes like the MuA transposase and several structural proteins like the capsid, tail, tail fiber, baseplate, neck. This phage also encodes a portal, a holin, and a L-Alanine-D-glutamine-peptidase protein.

“Mollyvirus Molly” species

Using the type strain *Maribacter forsetii* T (DSM 18668) the “Mollyvirus Molly” was isolated from two time points in 2017. Six highly similar 99.56 to 99.99% strains (Molly1-7) were obtained. The phage had a capsid diameter of 74.9 ± 3.6 nm, a tail length of 101.5 ± 6.3 nm, and a tail width of 18.1 ± 1.6 nm. The morphology was myoviral-like. The circular genome had 124,169 to 124,898 bp with a GC% of 36.2. This phage group was very difficult to sequence, which might be due to a high degree of DNA modifications indicated by the respective genes. Furthermore, genes encoding a baseplate, tail fiber, tail, tail sheath, tape measure, neck, portal, major capsid protein, and a DNA polymerase I were identified. Additionally, it had a ribonucleotide reductase with two subunits A and B and a relatively short (199 aa) zinc-dependent metallopeptidase, formed from a lipoprotein domain and the peptidase domain.

“Mollyvirus Colly” species

“Mollyvirus Colly” was 94% similar to “Mollyvirus Molly”. Following the ICTV guidelines it is a different species in the genus *Mollyvirus*. Colly had the same functional genes as Molly. The difference is due to genes encoding hypothetical proteins.

“Gundelvirus Gundel” species

The “Gundelvirus Gundel” was isolated twice, before and after the phytoplankton peak in 2018, with AHE14PA (DSM111040) and AHE15PA (DSM111039). It had a podoviral morphology with a capsid diameter of 60.5 ± 5.2 nm and a tail length of 22.7 ± 3.2 nm. Ten isolates were obtained, all having a genome size of 78,511 bp and a GC content of 30.4%. The circular genome had short direct terminal repeats (DTRs) at the ends. Gundel can only infect its two hosts of isolation, AHE14PA and AHE15PA, which had 99.87% similar 16S rRNA and an ANI of 99.99%. Gundel had genes coding for tail fiber, portal, L-alanine-D-glutamine-peptidase, and a phage antirepressor protein. In addition, Gundel had 10 tRNAs.

Tables and Figures

Figures

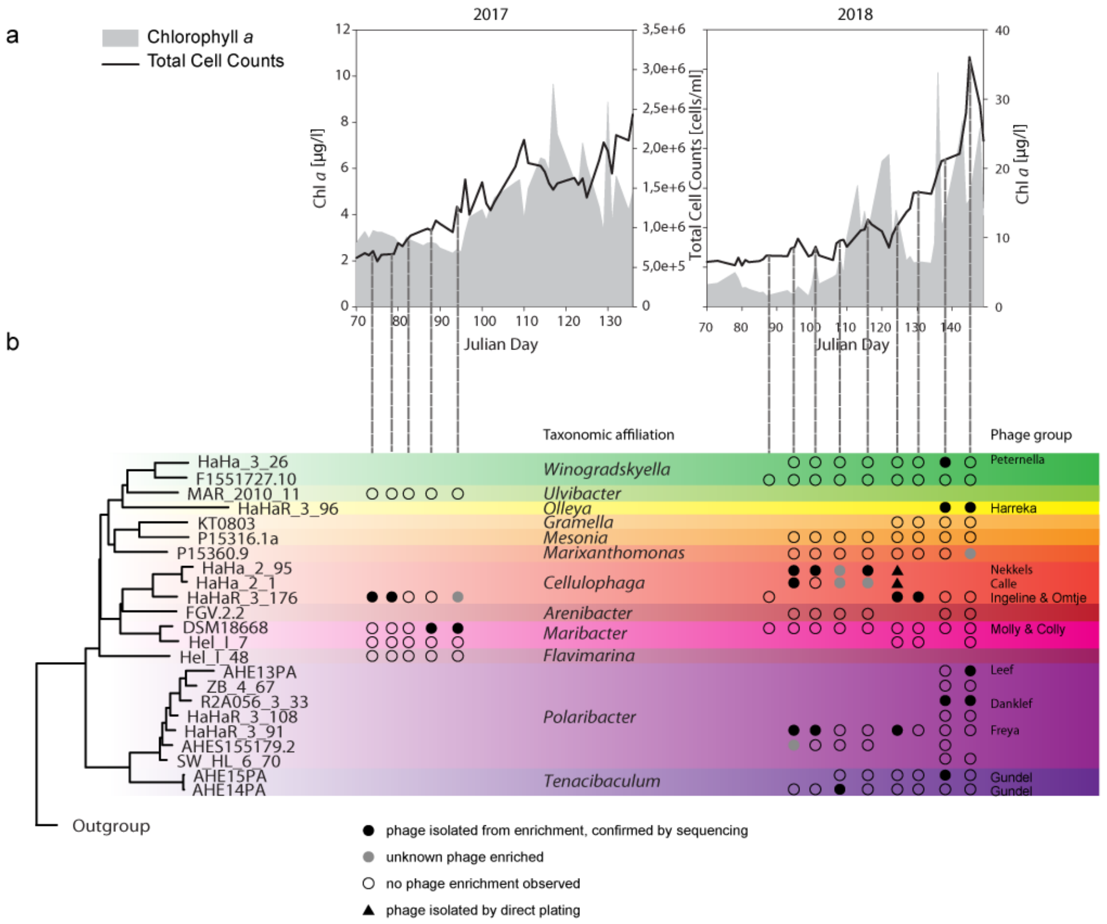

Figure 1: Chlorophyll *a* concentration and total cell counts during the sampling and phage enumeration with TEM and epifluorescence microscopy (a). Neighbor – joining tree with bacterial isolates and type strains on the basis of 16S rRNA and the isolation success of the corresponding phages (b).

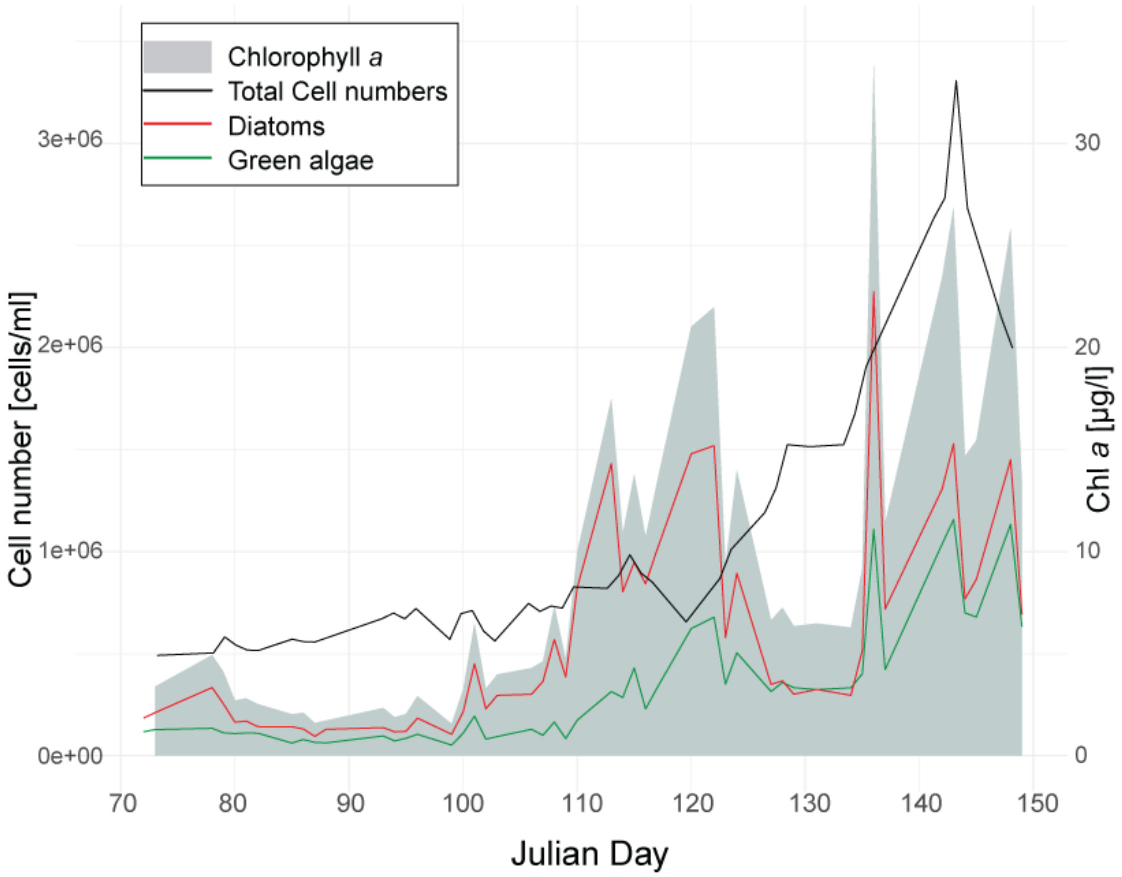

Figure 2: Pigment concentration of green algae and diatoms measured by fluorescence over the course of the bloom 2018.

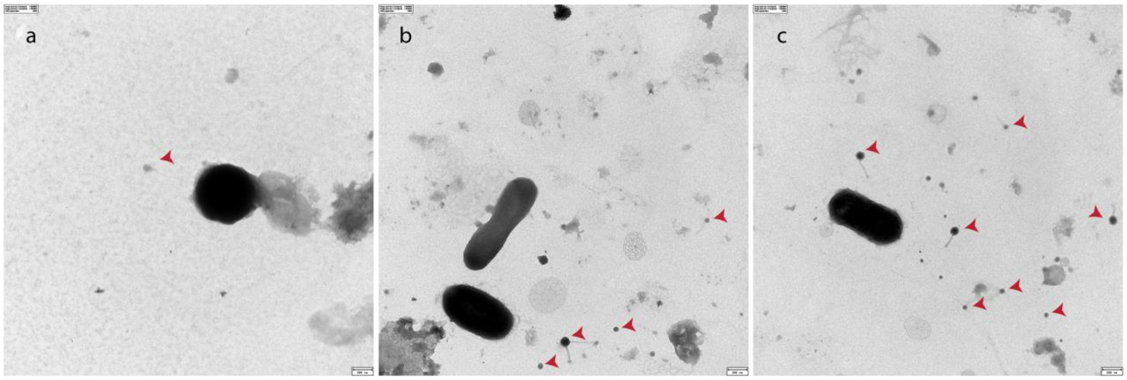

Figure 3: Example images of TEM virus counts from Julian Day 102 (a), 128 (b), and 144 (c). Phage particles are marked by arrowheads.

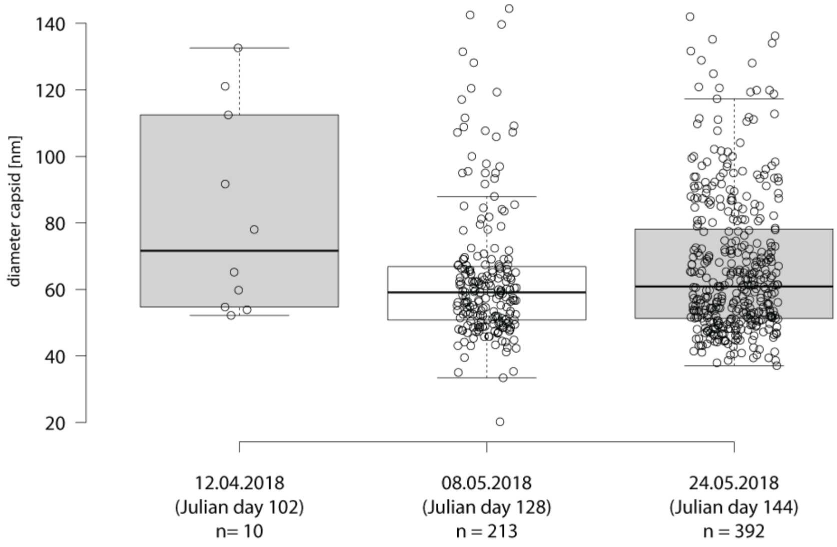

Figure 4: Capsid size distribution during the spring bloom 2018 from TEM images taken for virus counts.

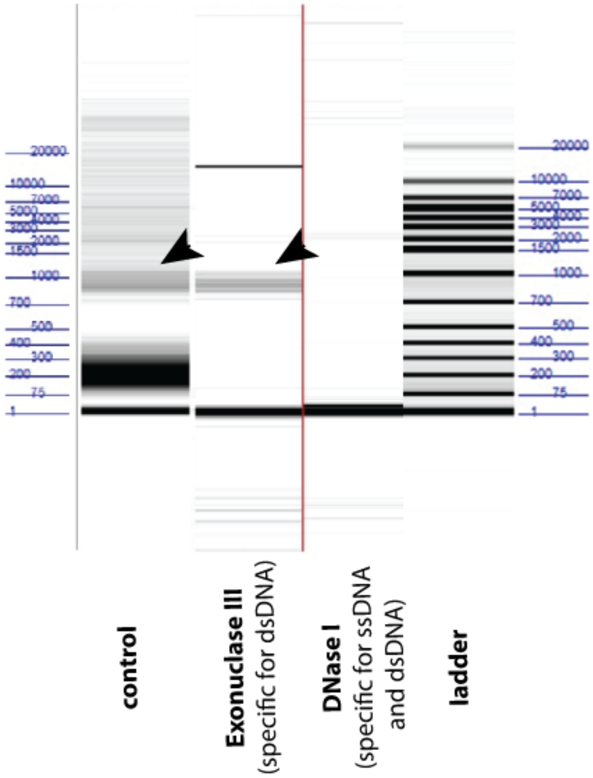

Figure 5: DNA digestion of *Cellulophaga* phage Omtje_1 visualized with Fragment Analyzer. Arrow heads indicate phage DNA band.

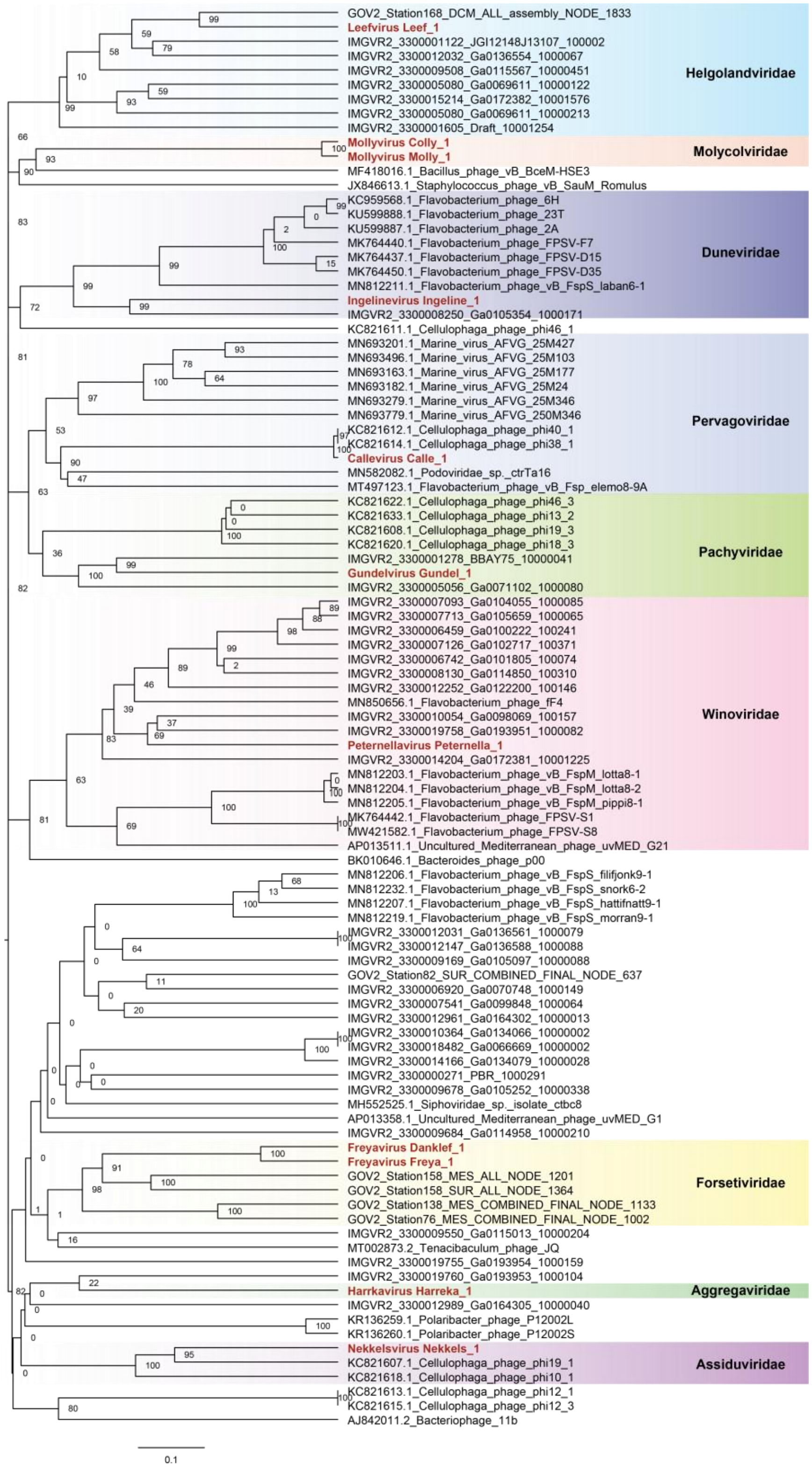

Figure 6: VirClust hierarchical clustering of the new dsDNA flavophages and their relatives, based on intergenomic distances calculated using the protein cluster content. Support values of selective inference (si (39)) for hierarchical clustering tree are indicated at branching points.

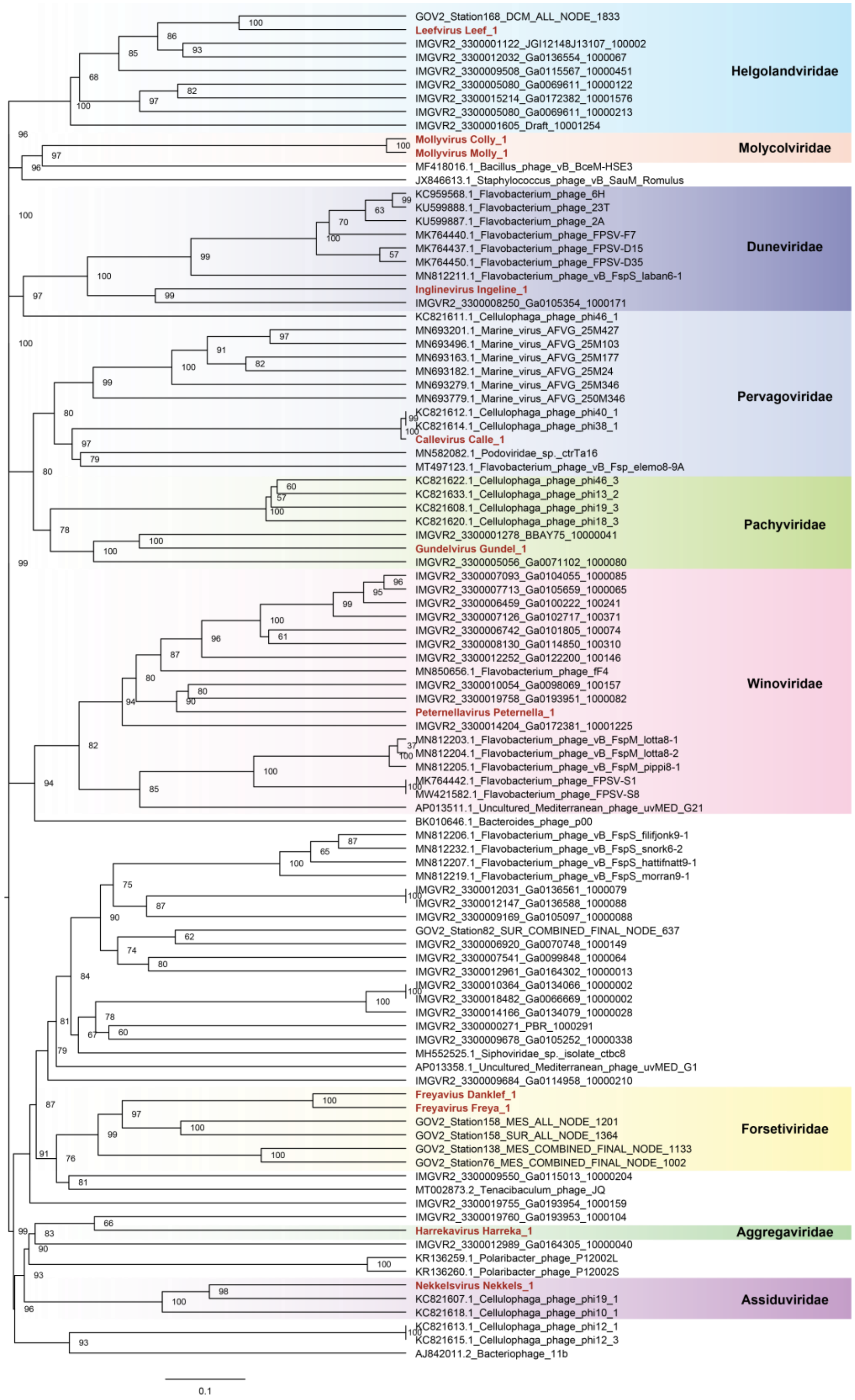

Figure 7. VirClust hierarchical clustering of the new dsDNA flavophages and their relatives, based on intergenomic distances calculated using the protein cluster content. Support values of approximately unbiased (au (40)) for hierarchical clustering tree are indicated at branching points.

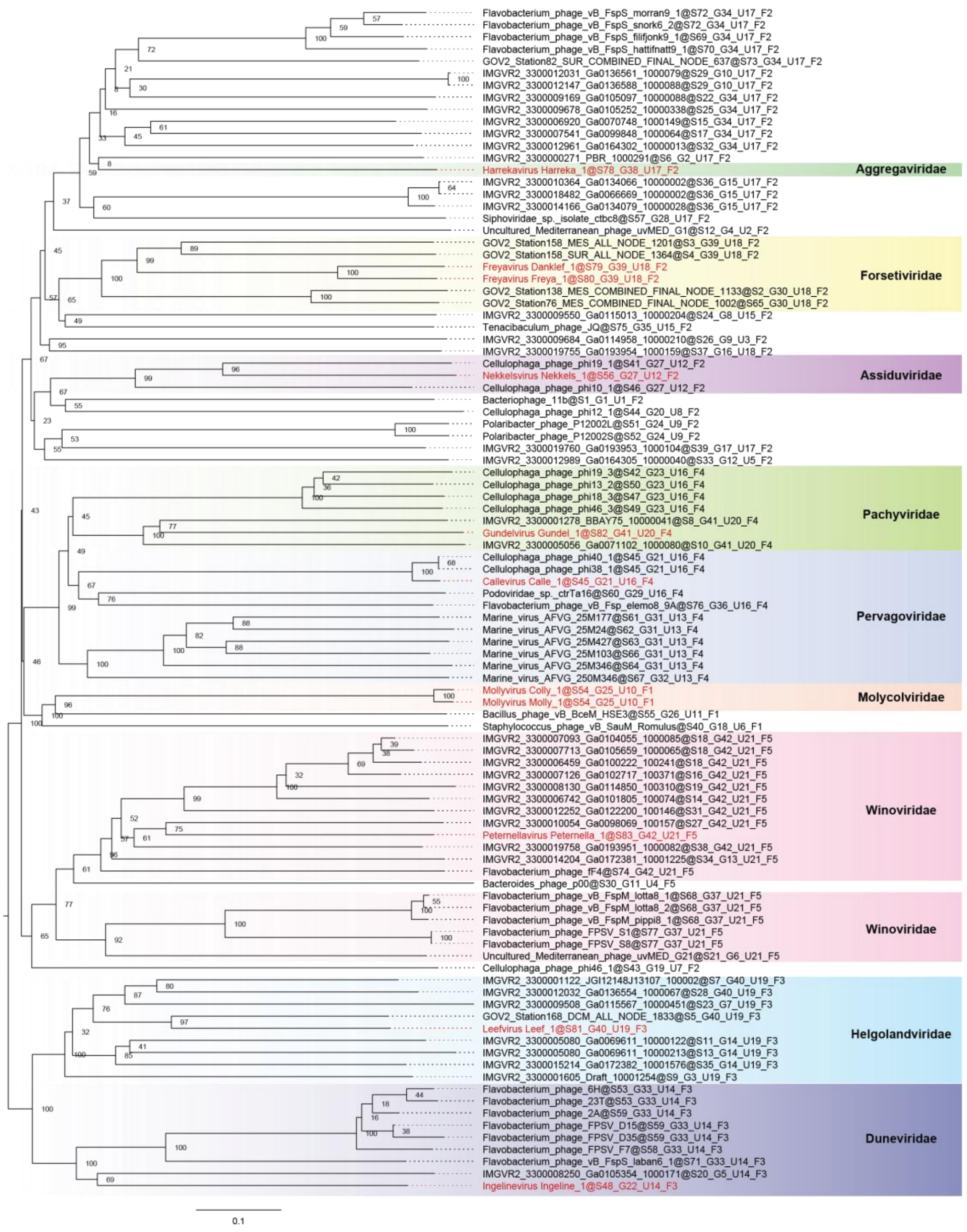

Figure 8: Whole-genome phylogeny determined with VICTOR (amino-acid based) for the dsDNA flavophages, including isolates and their relative phage genomes. Our new phage isolates are depicted in red. Pseudo-bootstrap values are indicated at branches. Family (F) and subfamily (U) clustering is indicated at the end of the genome names.

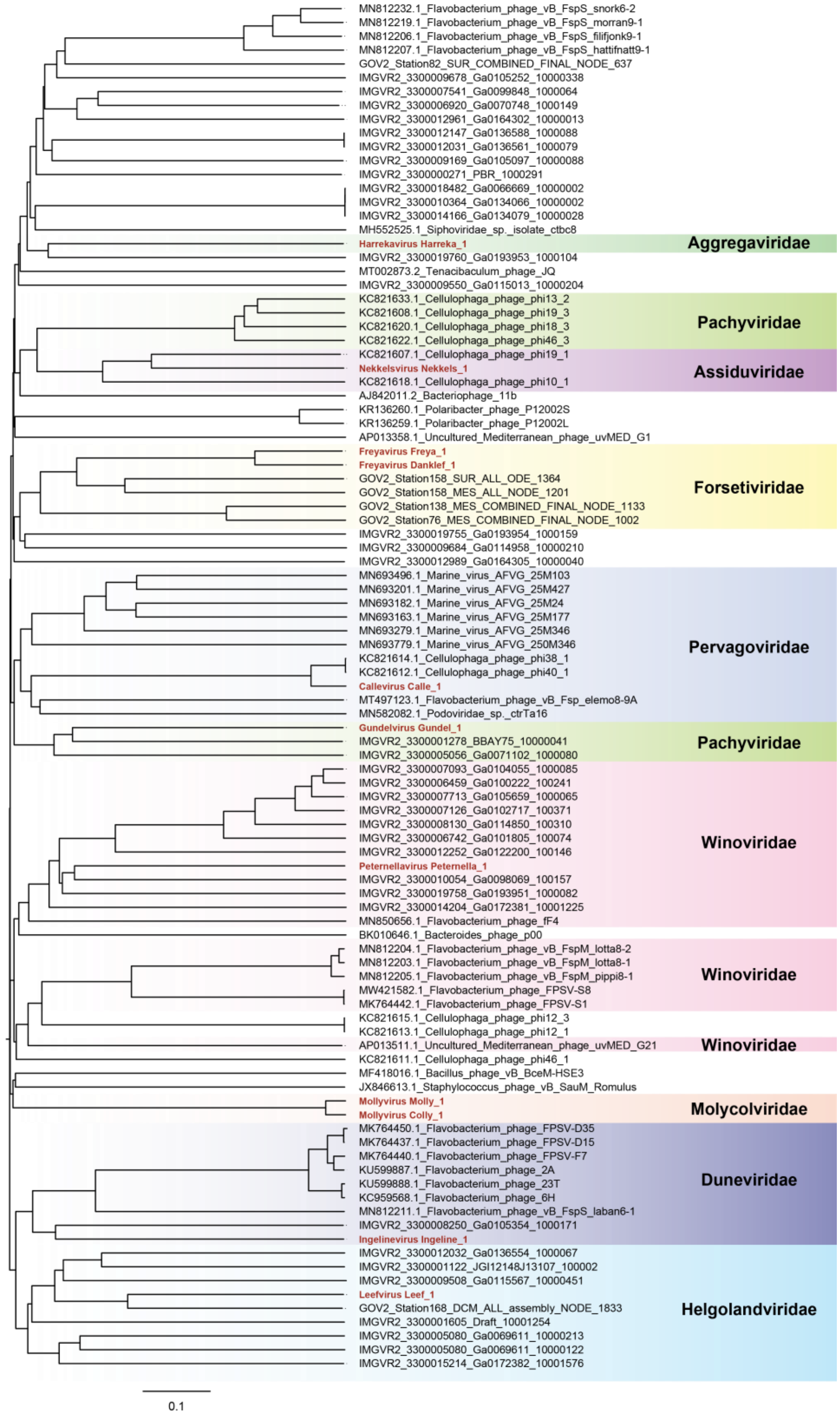

Figure 9: Whole-genome clustering determined with ViPTree (amino-acid based) for the dsDNA flavophages, including isolates and their relative phage genomes.

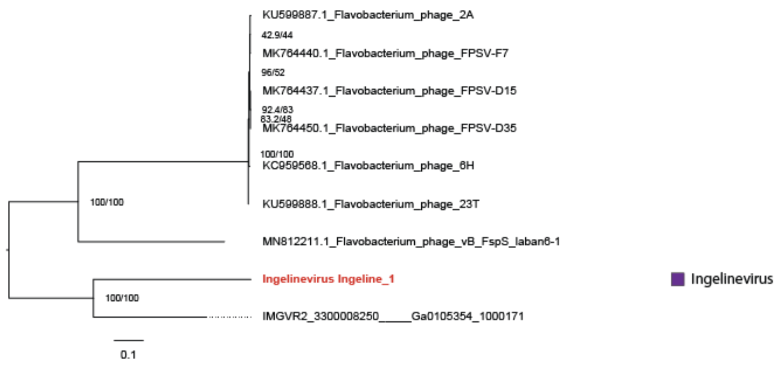

Figure 10: Core gene phylogeny of "Duneviridae" using MUSCLE aligned core proteins and IQ-Tree. This phylogeny is based on seven core genes which can be found in the annotation file of Ingeline in the following protein clusters: 3 (hp), 4 (hp), 5 (major capsid protein), 7 (hp), 8 (adaptor protein), 14 (hp), 15 (hp) (SI file 7). The first branch support value is the SH-aLRT support in %, the second value is the ultrafast bootstrap support. Using the model finder the VT+F+I substitution model was determined as best fitting substitution model and used for the tree calculation.

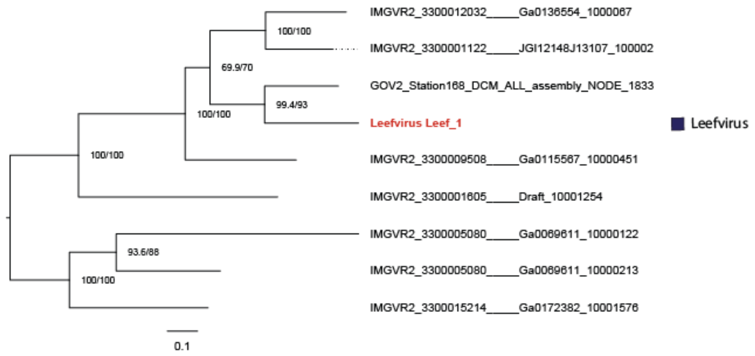

Figure 11: Core gene phylogeny of "Helgolandviridae" using MUSCLE aligned core proteins and IQ-Tree. This phylogeny is based on five core genes which can be found in the annotation file of Leef in the following protein clusters: 5 (hp), 9 (hp), 10 (hp), 11 (YceI family protein), 13 (hp) (SI file 7). The first branch support value is the SH-aLRT support in %, the second value is the ultrafast bootstrap support. Using the model finder the VT+F+I+G4 substitution model was determined as best fitting substitution model and used for the tree calculation.

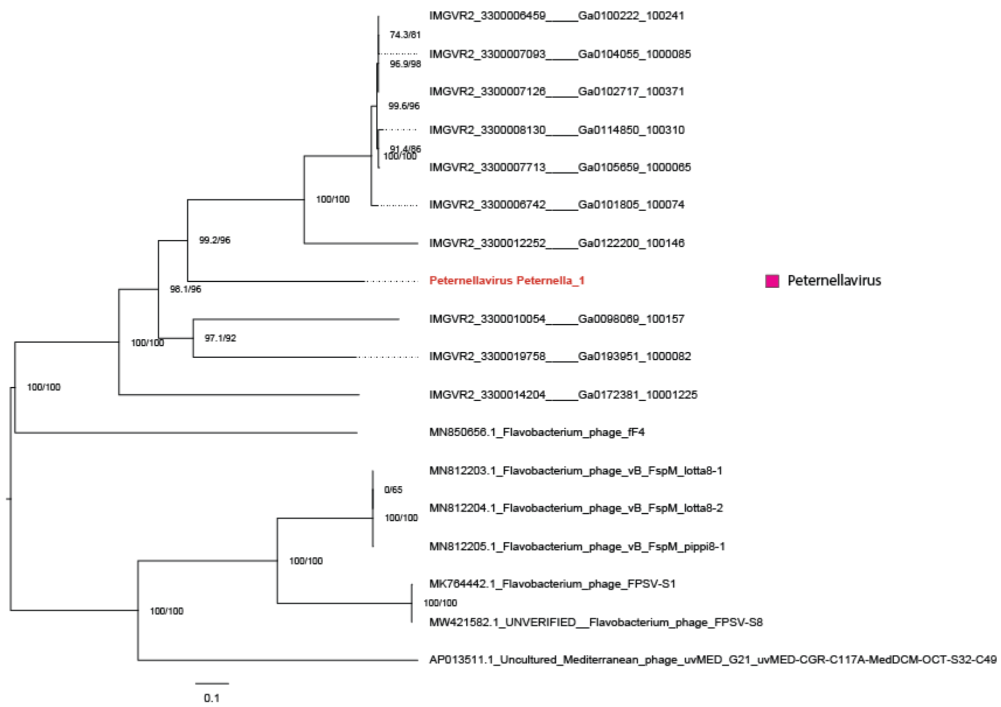

Figure 12; Core gene phylogeny of “Winoviridae” using MUSCLE aligned core proteins and IQ-Tree. This phylogeny is based on nine core genes which can be found in the annotation file of Peternella in the following protein clusters: 3 (major capsid protein), 4 (Clp protease), 6 (DUF2586/sheath), 8 (hp), 10 (Mu-like prophage protein gpG/neck), 11 (hp), 13 (phage protein D), 15 (oxidase), 18 (nucleotidyltransferase) (SI file 7). The first branch support value is the SH-aLRT support in %, the second value is the ultrafast bootstrap support. Using the model finder the VT+F+I+G4 substitution model was determined as best fitting substitution model and used for the tree calculation.

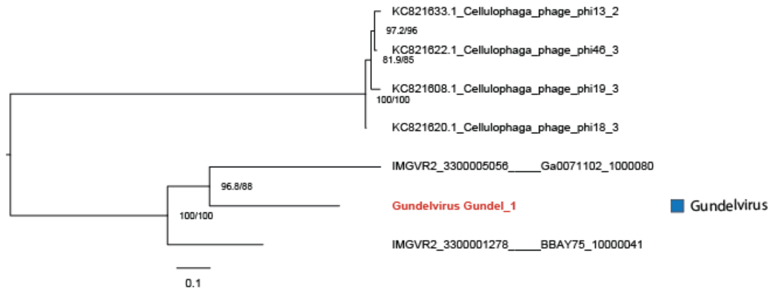

Figure 13: Core gene phylogeny of “Pachyviridae” using MUSCLE aligned core proteins and IQ-Tree. This phylogeny is based on nine core genes which can be found in the annotation file of Gundel in the following protein clusters: 4 (hp), 5 (structural protein), 6 (structural protein), 7 (hp), 8 (portal protein), 9 (structural protein), 10 (structural protein), 11 (hp), 12 (hp) (SI file 7). The first branch support value is the SH-aLRT support in %, the second value is the ultrafast bootstrap support. Using the model finder the VT+F+G4 substitution model was determined as best fitting substitution model and used for the tree calculation.

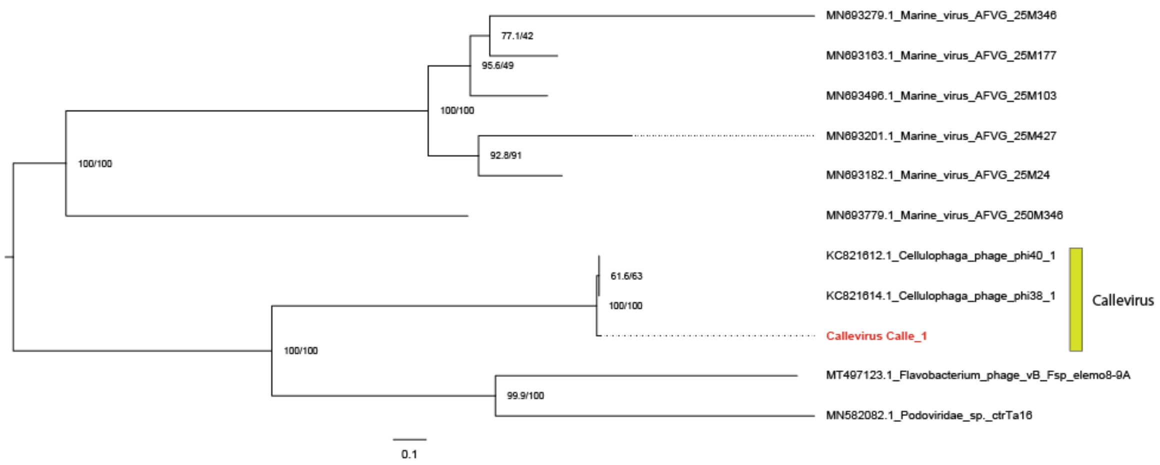

Figure 14: Core gene phylogeny of "Pervagoviridae" using MUSCLE aligned core proteins and IQ-Tree. This phylogeny is based on three core genes which can be found in the annotation file of Calle in the following protein clusters: 4 (chaperonin cpn10), 8 (structural protein), 13 (structural protein) (SI file 7). The first branch support value is the SH-aLRT support in %, the second value is the ultrafast bootstrap support. Using the model finder the LG+F+G4 substitution model was determined as best fitting substitution model and used for the tree calculation.

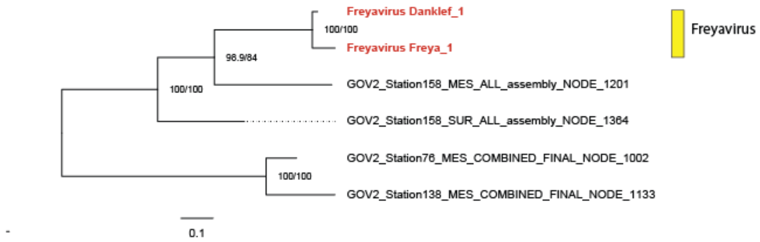

Figure 15: Core gene phylogeny of "Pervagoviridae" using MUSCLE aligned core proteins and IQ-Tree. This phylogeny is based on seven core genes which can be found in the annotation file of Freya in the following protein clusters: 1 (tape measure protein), 3 (structural protein), 4 (portal protein), 5 (structural protein), 7(structural protein), 11 (structural protein), 17 (major capsid protein) (SI file 7). The first branch support value is the SH-aLRT support in %, the second value is the ultrafast bootstrap support. Using the model finder the VT+F+I substitution model was determined as best fitting substitution model and used for the tree calculation.

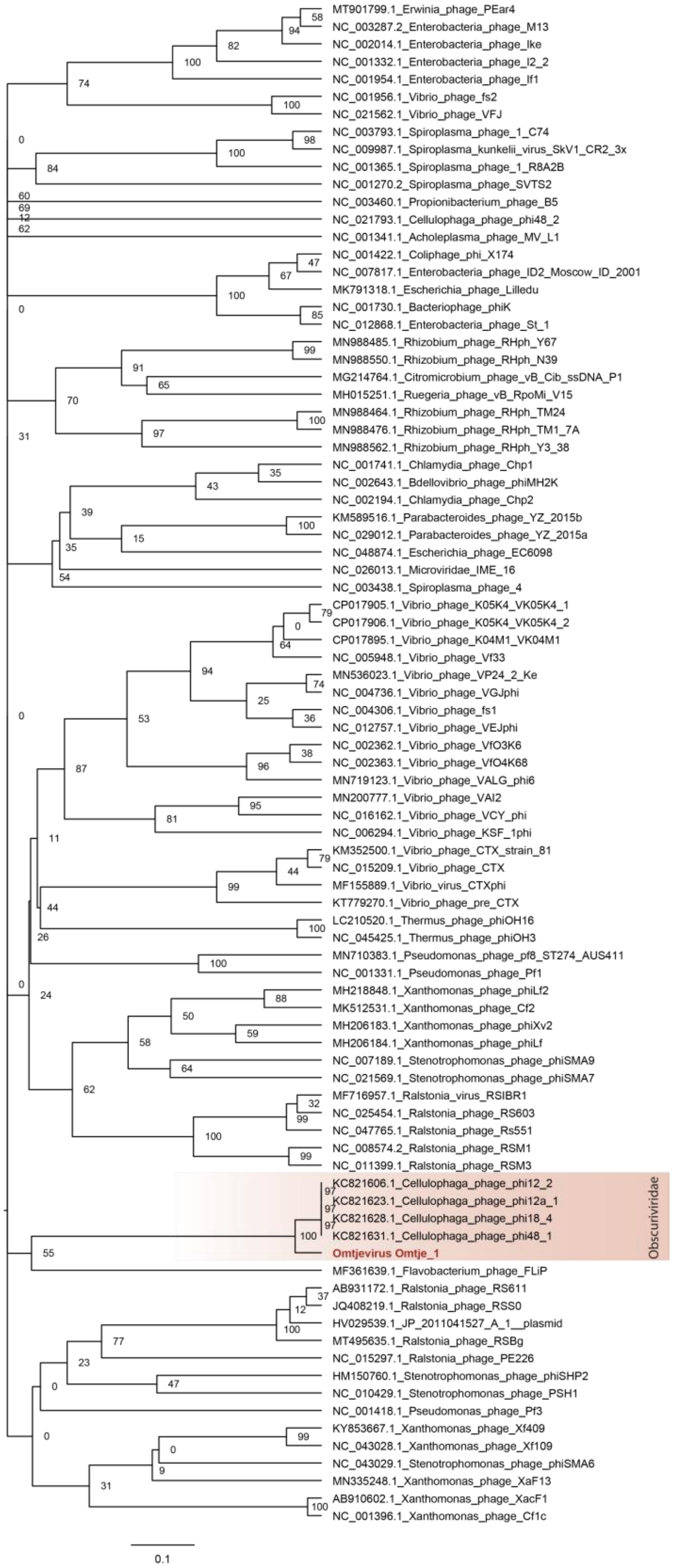

Figure 16: VirClust hierarchical clustering of the new ssDNA flavophages and their relatives, based on intergenomic distances calculated using the protein cluster content. Support values of selective inference (si (39)) for hierarchical clustering tree are indicated at branching points.

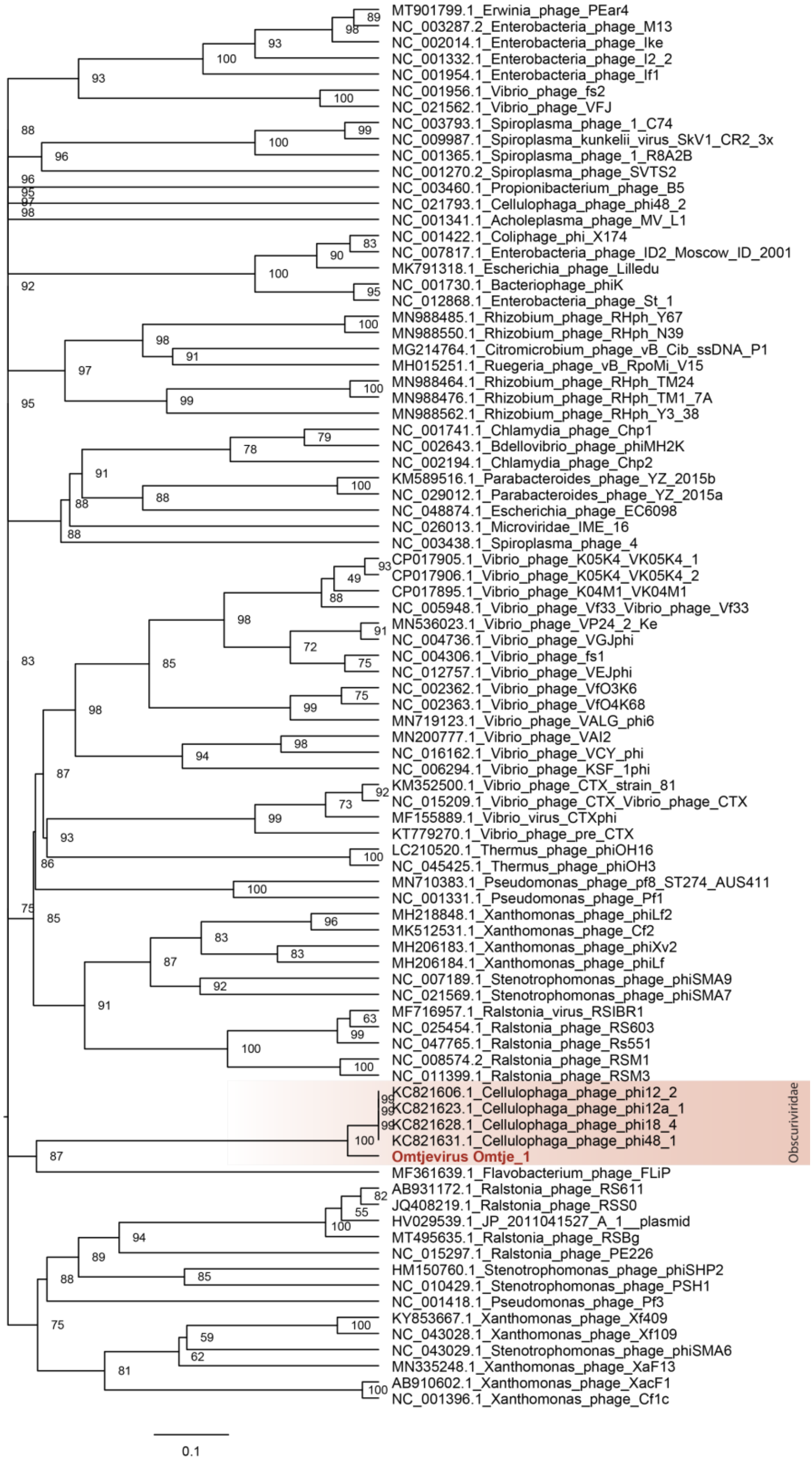

Figure 17: VirClust hierarchical clustering of the new ssDNA flavophages and their relatives, based on intergenomic distances calculated using the protein cluster content. Support values of approximately unbiased (au (40)) for hierarchical clustering tree are indicated at branching points.

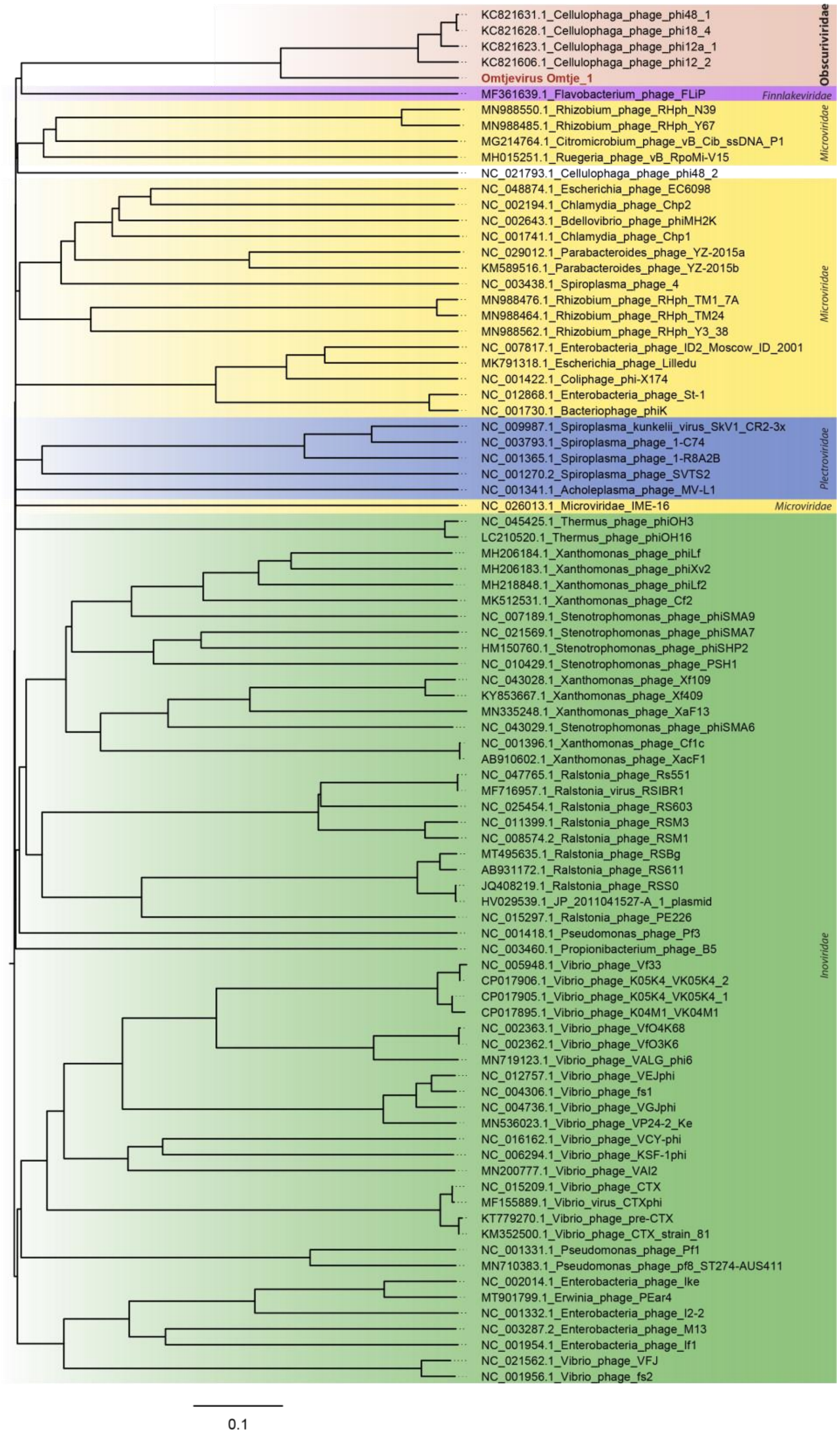

Figure 18: ViPTree of ssDNA viruses with our new phage isolate depicted in red.

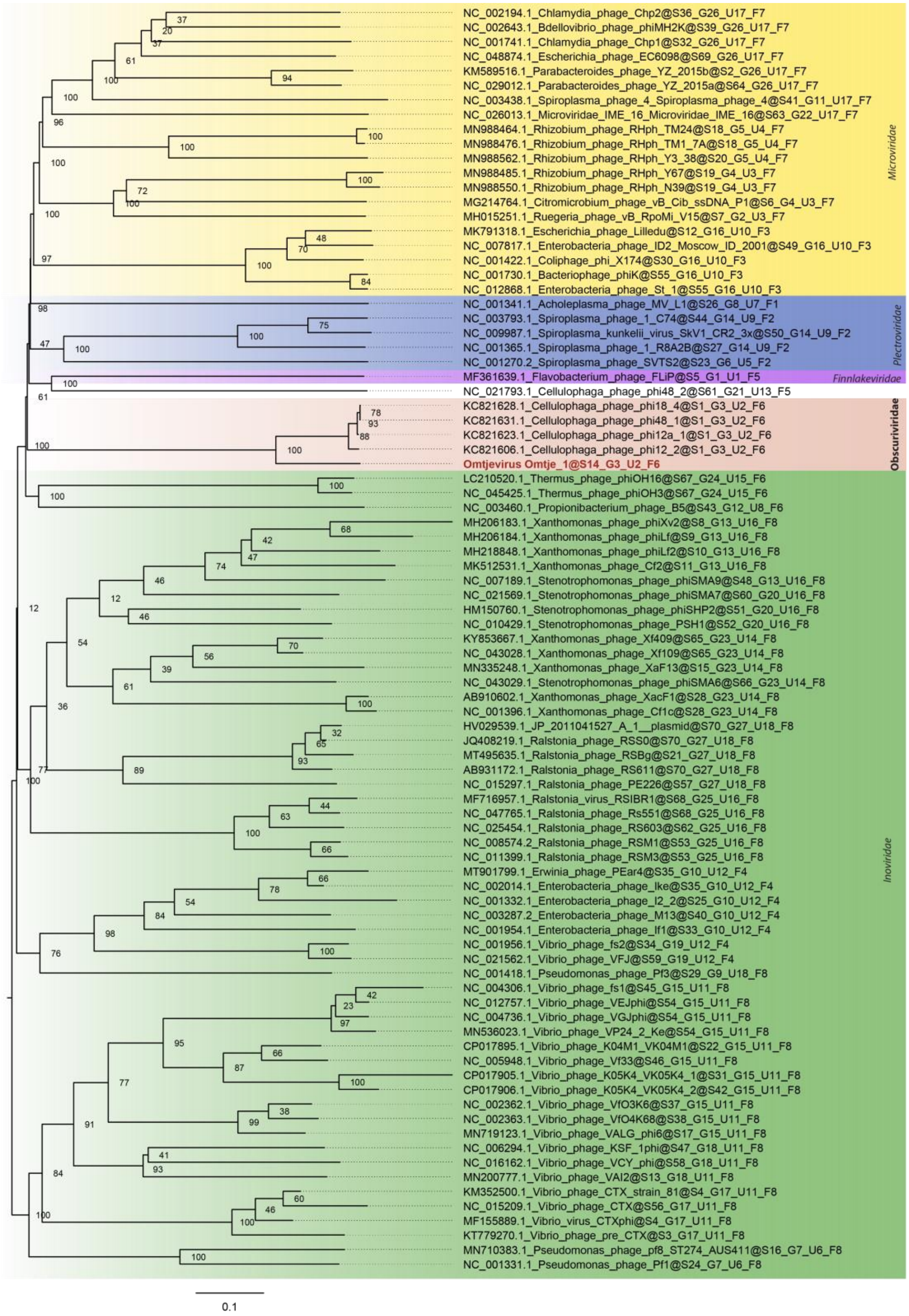

Figure 19: Whole-genome phylogeny determined with VICTOR (amino-acid based) for the ssDNA phages. Our new phage isolate is depicted in red. Pseudo-bootstrap values are indicated at branches. Family (F) and subfamily (U) clustering is indicated at the end of the genome names.

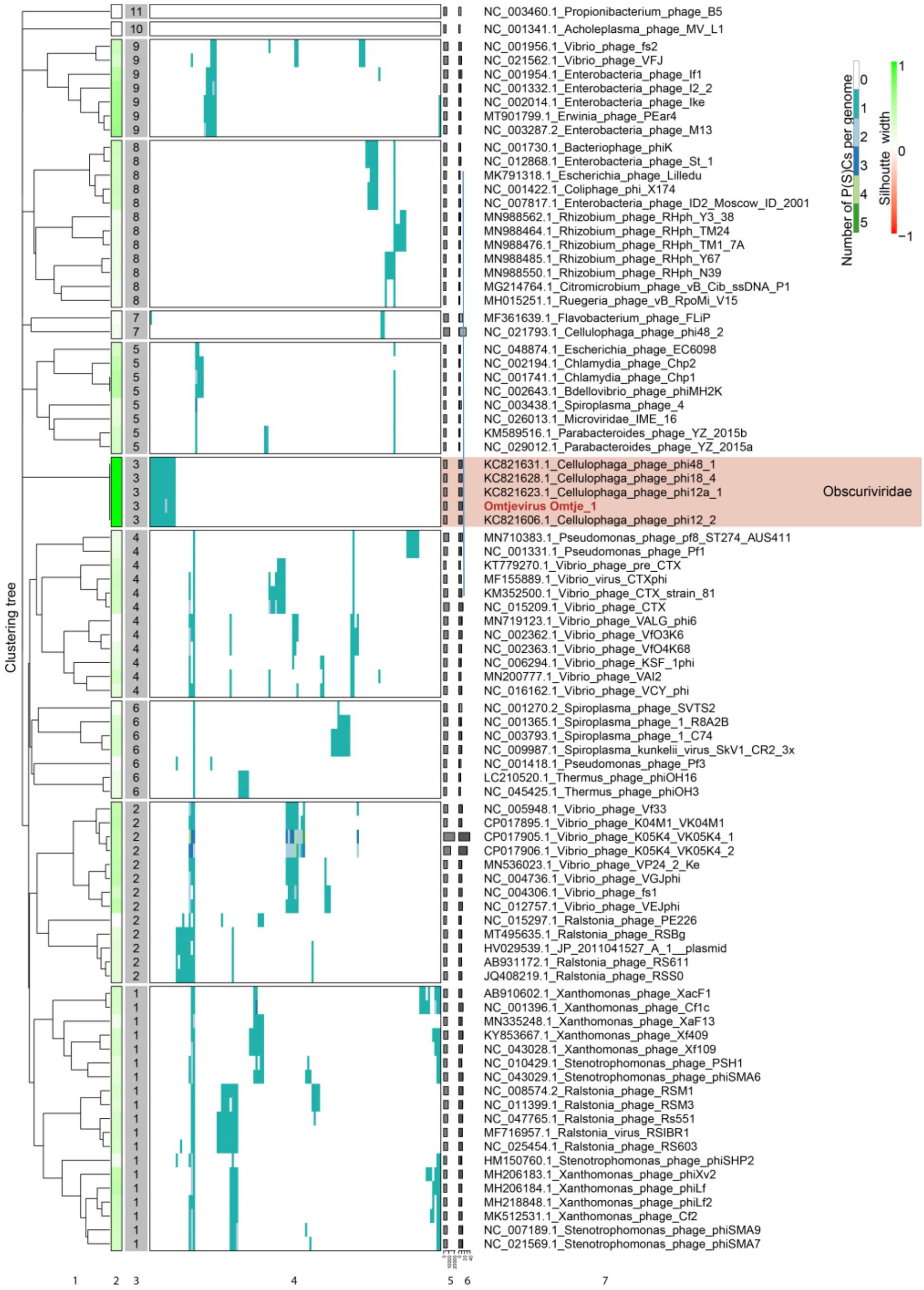

Figure 20: VirClust hierarchical clustering of the new **s**sDNA flavophages and their relatives, based on intergenomic distances calculated using the protein super-cluster content (PC clusters were grouped into super-clusters based on HMM profile homology). 1. Hierarchical clustering tree. The tree was cut into smaller viral genome clusters (VGCs) using a 0.9 distance threshold. Each VGC is framed in a rectangle in 2 and 3. 2. Silhouette width, measures how related is a virus with other viruses in the same VGCs. Similarity to other VGCs is indicated by values closer to -1 (red). Similarity to viruses in the same VGC is indicated by values closer to 1 (green). 3. Distribution of the protein super-clusters (PSCs) in the viral genomes. 4. Genome length (bps). 5. Fraction of proteins shared with other viruses (dark grey), based on protein assignment to PSCs. 6. Virus names; flavophage isolated in this study marked in red.

To note: The "Obscuriviridae" share only one protein with a phage from another cluster (the Flavobacterium phage FLIP).

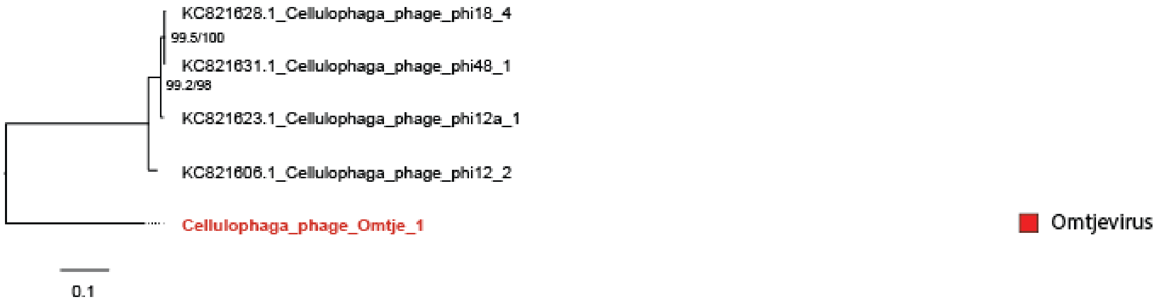

Figure 21: Core gene phylogeny of "Obscuriviridae" using MUSCLE aligned core proteins and IQ-Tree. This phylogeny is based on seven core genes which can be found in the annotation file of Omtje in the following protein clusters: 1 (hp), 2 (structural protein), 8 (structural protein), 9 (structural protein), 10 (structural protein), 11 (structural protein), 3 (replication initiation factor), 4 (structural protein), 5 (structural protein), 6 (structural protein), 7 (mannosyl-glycoprotein endo-beta-N-acetylglucosaminidase) (SI file 7). The first branch support value is the SH-aLRT support in %, the second value is the ultrafast bootstrap support. Using the model finder the LG+F+G4 substitution model was determined as best fitting substitution model and used for the tree calculation.

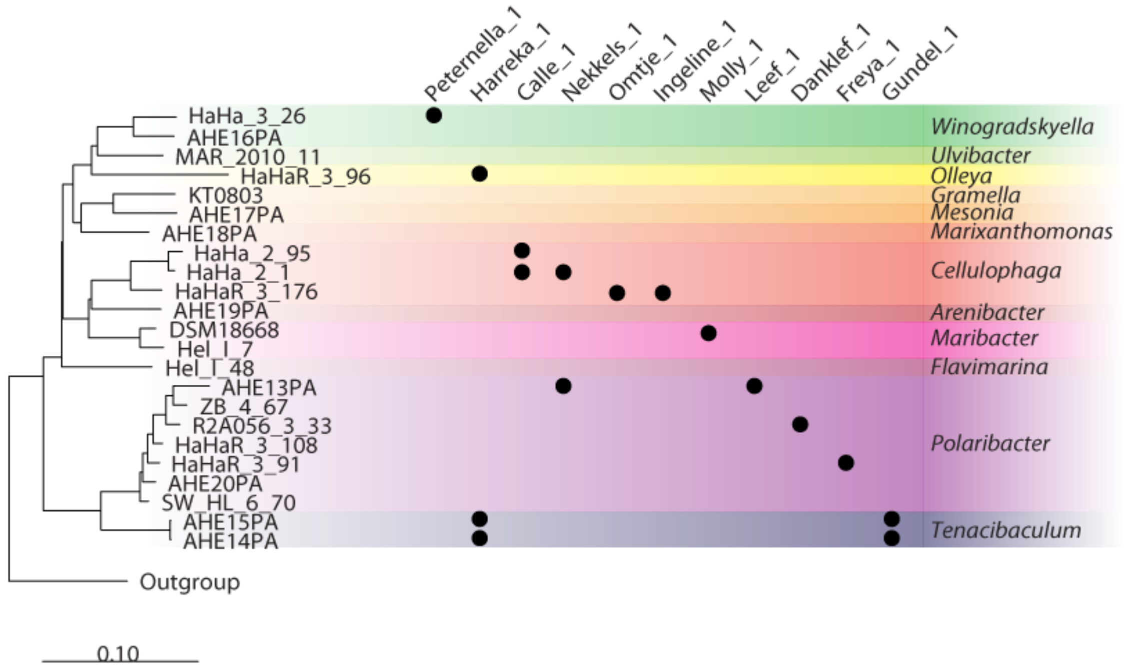

Figure 22: Determination of host range using all bacterial isolates, which were used as isolation source for phages in 2017 and 2018. Bacterial isolates are clustered in a 16S rRNA neighbor joining tree and on the top are the phages sorted by the host order in the tree. Colly_1 was not tested.

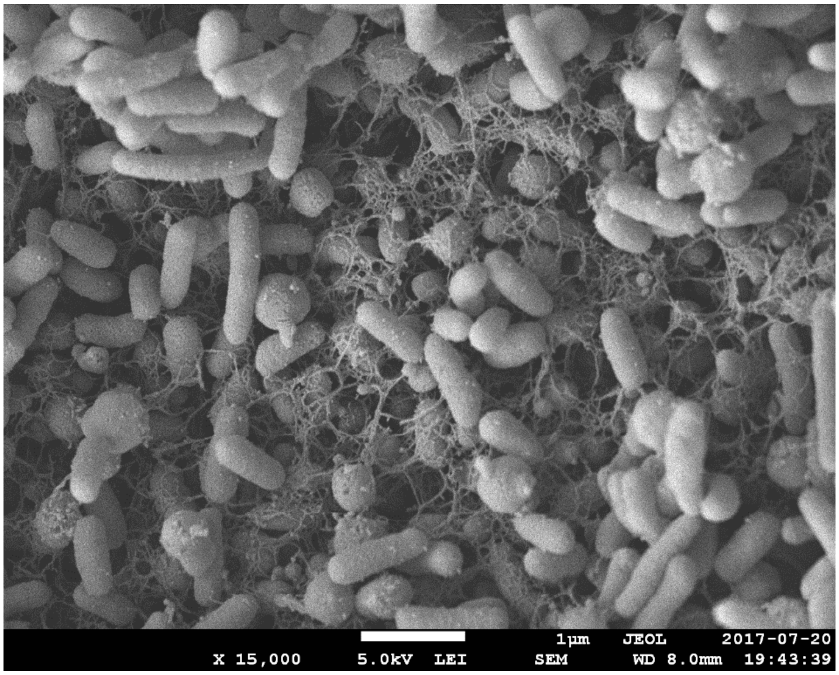

Figure 23: SEM image of *Cellulophaga* sp. HaHaR_3_176 colony grown on marine broth plates. The biofilm produced by the bacteria is visible as a fibrillar meshwork.

Tables

Table 1: Bacteria used for flavophage isolation in 2017 and 2018 and genome, 16S rRNA gene and culture collection accession numbers.

Genus	Strain	DSMZ accession number	Genome accession number from this study	Year of isolation	Associated studies	GenBank accession number for 16S sequence	Used in 2017	Used in 2018
Gramella	KT0803	DSM17595		1999	(41)	AF235117.1	+	+
Cellulophaga	HaHaR_3_176	DSM111152	CP058990	2016	(42)	LT724228.1	+	+
Maribacter	Hel_1_7	-		2010	(30)	JX854136.1	+	+
Maribacter	KT02ds 18-6	DSM18668		1998	(43)	AM712900.1	+	+
Leeuwenhoekiella	Hel_1_48	-		2010	(30)	JX854131.1	+	-
Ulvibacter	Mar_2010_11	-		2010	(30)	JX854389.1	+	-
Winogradskyella	AHE16PA	-		2017	this study	MT667377	-	+
Mesonia	AHE17PA	-		2017	this study	MT667380	-	+
Marixanthomonas	AHE18PA	-		2017	this study	MT667381	-	+
Winogradskyella	HaHa_3_26	DSM111041	CP058981	2016	(42)	MT704618	-	+
Cellulophaga	HaHa_2_1	DSM111038	CP058989	2016	(42)	MT704616	-	+
Polaribacter	HaHaR_3_91	DSM111048	CP058986	2016	(42)	MT704619	-	+
Cellulophaga	HaHa_2_95	DSM111037	CP058988	2016	(42)	MT704617	-	+
Arenibacter	AHE19PA	-		2017	this study	MT667378	-	+
Tenacibaculum	AHE14PA	DSM111040	CP058983	2017	this study	MT704614	-	+
Polaribacter	AHE20PA	-		2017	this study	MT667376	-	+
Tenacibaculum	AHE15PA	DSM111039	CP058982	2017	this study	MT704615	-	+
Polaribacter	HaHaR_3_108	-		2016	(42)	MT667379	-	+
Olleya	HaHaR_3_96	DSM111044	CP058987	2016	(42)	MT704620	-	+
Polaribacter	R2A056_3_33	DSM111047	CP058984	2016	(42)	MT704621	-	+
Polaribacter	SW_HL_6_70	-		2016	(42)	MT667382	-	+
Polaribacter	ZB_4_67	-		2016	(42)	MT667383	-	+
Polaribacter	AHE13PA	DSM111061	CP058985	2017	this study	MT704613	-	+

Table 2: Detailed information of metagenomes used for read mapping

Julian day	Date	Size fraction	Accession number
76	16.03.2016	0.2 - 3 µm	PRJNA441607
81	21.03.2016	0.2 - 3 µm	PRJNA441608
91	31.03.2016	0.2 - 3 µm	PRJNA441609
103	12.04.2016	0.2 - 3 µm	PRJNA441610
110	19.04.2016	0.2 - 3 µm	PRJNA441611
117	26.04.2016	0.2 - 3 µm	PRJNA441612
123	02.05.2016	0.2 - 3 µm	PRJNA441613
133	12.05.2016	0.2 - 3 µm	PRJNA441614
138	17.05.2016	0.2 - 3 µm	PRJNA441615
78	19.03.2018	0.2 - 3 µm	ERX4297004
		3 - 10 µm	ERX4297022
		> 10 µm	ERX4297030
93	03.04.2018	0.2 - 3 µm	ERX4297005
95	05.04.2018	0.2 - 3 µm	ERX4297006
100	10.04.2018	0.2 - 3 µm	ERX4297007
102	12.04.2018	0.2 - 3 µm	ERX4297008
		3 - 10 µm	ERX4297023
		> 10 µm	ERX4297031
107	17.04.2018	0.2 - 3 µm	ERX4297009
		3 - 10 µm	ERX4297024
		> 10 µm	ERX4297032
109	19.04.2018	0.2 - 3 µm	ERX4297010
114	24.04.2018	0.2 - 3 µm	ERX4297011
116	26.04.2018	0.2 - 3 µm	ERX4297012
		3 - 10 µm	ERX4297025
		> 10 µm	ERX4297033
122	02.05.2018	0.2 - 3 µm	ERX4297013
123	03.05.2018	0.2 - 3 µm	ERX4297014
128	08.05.2018	0.2 - 3 µm	ERX4297015
		3 - 10 µm	ERX4297026
		> 10 µm	ERX4297034
131	11.05.2018	0.2 - 3 µm	ERX4297016
		3 - 10 µm	ERX4297027
		> 10 µm	ERX4297035
135	15.05.2018	0.2 - 3 µm	ERX4297017
137	17.05.2018	0.2 - 3 µm	ERX4297018
142	22.05.2018	0.2 - 3 µm	ERX4297019
		3 - 10 µm	ERX4297028
		> 10 µm	ERX4297036
144	24.05.2018	0.2 - 3 µm	ERX4297020
149	29.05.2018	0.2 - 3 µm	ERX4297021
		3 - 10 µm	ERX4297029
		> 10 µm	ERX4297037

Table 3: Phage genome accession numbers of strains and culture collection accession numbers.

Organism	Genome Accession Number	DSMZ Accession Number
Cellulophaga_phage_Calle_1	MT732432	DSM111231
Cellulophaga_phage_Calle_2	MT732433	
Cellulophaga_phage_Calle_3	MT732434	
Cellulophaga_phage_Ingeline_1	MT732435	DSM111236
Cellulophaga_phage_Ingeline_2	MT732436	
Cellulophaga_phage_Ingeline_3	MT732437	
Cellulophaga_phage_Ingeline_4	MT732438	
Cellulophaga_phage_Ingeline_5	MT732439	
Cellulophaga_phage_Ingeline_6	MT732440	
Cellulophaga_phage_Ingeline_7	MT732441	
Cellulophaga_phage_Ingeline_8	MT732442	
Cellulophaga_phage_Nekkels_1	MT732443	DSM11123
Cellulophaga_phage_Nekkels_2	MT732444	
Cellulophaga_phage_Omtje_1	MT732445	DSM111240
Cellulophaga_phage_Omtje_2	MT732446	
Cellulophaga_phage_Omtje_3	MT732447	
Cellulophaga_phage_Omtje_4	MT732448	
Cellulophaga_phage_Omtje_5	MT732449	
Maribacter_phage_Colly_1	MT732450	DSM111252
Maribacter_phage_Molly_1	MT732451	DSM111257
Maribacter_phage_Molly_2	MT732452	
Maribacter_phage_Molly_3	MT732453	
Maribacter_phage_Molly_4	MT732454	
Maribacter_phage_Molly_5	MT732455	
Maribacter_phage_Molly_7	MT732456	
Olleya_phage_Harreka_1	MT732457	DSM111256
Polaribacter_phage_Danklef_1	MT732458	DSM111232
Polaribacter_phage_Danklef_2	MT732459	
Polaribacter_phage_Danklef_3	MT732460	
Polaribacter_phage_Danklef_4	MT732461	
Polaribacter_phage_Danklef_5	MT732462	
Polaribacter_phage_Freya_1	MT732463	DSM111233
Polaribacter_phage_Freya_2	MT732464	
Polaribacter_phage_Freya_3	MT732465	
Polaribacter_phage_Freya_4	MT732466	
Polaribacter_phage_Freya_5	MT732467	
Polaribacter_phage_Freya_6	MT732468	
Polaribacter_phage_Freya_7	MT732469	
Polaribacter_phage_Freya_8	MT732470	
Polaribacter_phage_Freya_9	MT732471	
Polaribacter_phage_Freya_10	MT732472	DSM111234
Polaribacter_phage_Leef_1	MT732473	DSM111238
Tenacibaculum_phage_Gundel_1	MT732474	DSM111235
Winogradskyella_phage_Peternella_1	MT732475	DSM111241

Table 4: Virus to bacteria ratios for Julian day 102, 128, and 144 using transmission electron microscopy (TEM) and epifluorescence light microscopy (LM).

Julian day	TEM	LM
102	1.8	36.3
128	2.6	79.3
144	3.1	52.3

Table 5: List of obtained flavophage isolates**.**

Phage isolation name	Culture accession	Sampling date	Host	Host genus	Phage subgroup	Phage group	Genome size
E30_2/2/2/1/F	DSM111231	03.04.2018	HaHa_2_95	*Cellulophaga*	Calle_1	Calle	72979
E30_2/2/2/2/F		03.04.2018	HaHa_2_95	*Cellulophaga*	Calle_2	Calle	72979
Re30_10/1s/1 final		03.05.2018	HaHa_2_95	*Cellulophaga*	Calle_3	Calle	72980
E30_2/1/1/F		03.04.2018	HaHa_2_95	*Cellulophaga*	Calle_2	Calle	72979
E47_12/2/1 final	DSM111232	15.05.2018	R2A056_3_33	*Polaribacter*	Danklef_1	Danklef	47186
E47_12/1/1 final		15.05.2018	R2A056_3_33	*Polaribacter*	Danklef_2	Danklef	47302
E47_12/3/1 final		15.05.2018	R2A056_3_33	*Polaribacter*	Danklef_2	Danklef	47302
E47_14/2/1 final		24.05.2018	R2A056_3_33	*Polaribacter*	Danklef_3	Danklef	47396
E47_14/3/1 final		24.05.2018	R2A056_3_33	*Polaribacter*	Danklef_4	Danklef	47426
E47_14/1/1 final		24.05.2018	R2A056_3_33	*Polaribacter*	Danklef_5	Danklef	48177
E27_2/2/1 final	DSM111233	03.04.2018	HaHaR_3_91	*Polaribacter*	Freya_1	Freya	43978
E27_2/2/2 final		03.04.2018	HaHaR_3_91	*Polaribacter*	Freya_1	Freya	43978
E27_4/1/2 final	DSM111234	10.04.2018	HaHaR_3_91	*Polaribacter*	Freya_10	Freya	48920
E27_8/1s/1 final		24.04.2018	HaHaR_3_91	*Polaribacter*	Freya_2	Freya	44820
E27_8/2s/1 final		24.04.2018	HaHaR_3_91	*Polaribacter*	Freya_3	Freya	44820
E27_8/2s/2 final		24.04.2018	HaHaR_3_91	*Polaribacter*	Freya_3	Freya	44820
E27_8/2s/3 final		24.04.2018	HaHaR_3_91	*Polaribacter*	Freya_3	Freya	44820
E27_8/2s/4 final		24.04.2018	HaHaR_3_91	*Polaribacter*	Freya_3	Freya	44820
E27_8/2s/5 final		24.04.2018	HaHaR_3_91	*Polaribacter*	Freya_3	Freya	44820
E27_8/2s/6 final		24.04.2018	HaHaR_3_91	*Polaribacter*	Freya_3	Freya	44820
E27_8/3s/2 final		24.04.2018	HaHaR_3_91	*Polaribacter*	Freya_3	Freya	44820
E27_8/3s/1 final		24.04.2018	HaHaR_3_91	*Polaribacter*	Freya_4	Freya	44820
E27_2/1/1 final		03.04.2018	HaHaR_3_91	*Polaribacter*	Freya_5	Freya	45722
E27_2/1/2 final		03.04.2018	HaHaR_3_91	*Polaribacter*	Freya_5	Freya	45722
E27_2/2/1/F		03.04.2018	HaHaR_3_91	*Polaribacter*	Freya_5	Freya	45722
E27_2/3/1 final		03.04.2018	HaHaR_3_91	*Polaribacter*	Freya_5	Freya	45722
E27_2/3/2 final		03.04.2018	HaHaR_3_91	*Polaribacter*	Freya_5	Freya	45722
E27_2/3/3 final		03.04.2018	HaHaR_3_91	*Polaribacter*	Freya_5	Freya	45722
E27_4/2/1 final		10.04.2018	HaHaR_3_91	*Polaribacter*	Freya_6	Freya	46194
E27_10/1s/1 final		03.05.2018	HaHaR_3_91	*Polaribacter*	Freya_7	Freya	46194
E27_4/1/3 final		10.04.2018	HaHaR_3_91	*Polaribacter*	Freya_8	Freya	48018
E27_2/1/1/F		03.04.2018	HaHaR_3_91	*Polaribacter*	Freya_9	Freya	48613
E27_4/1/1 final		10.04.2018	HaHaR_3_91	*Polaribacter*	Freya_9	Freya	48613
E27_4/3/1 final		10.04.2018	HaHaR_3_91	*Polaribacter*	Freya_9	Freya	48613
E37_7/1s/1 final	DSM111235	19.04.2018	AHE14PA	*Tenacibaculum*	Gundel_1	Gundel	78511
E37_7/1s/2 final		19.04.2018	AHE14PA	*Tenacibaculum*	Gundel_1	Gundel	78511
E37_7/1s/3 final		19.04.2018	AHE14PA	*Tenacibaculum*	Gundel_1	Gundel	78511
E42_11/1/1 final		08.05.2018	AHE15PA	*Tenacibaculum*	Gundel_1	Gundel	78511
E42_11/1s/1 final		08.05.2018	AHE15PA	*Tenacibaculum*	Gundel_1	Gundel	78511
E42_11/2/1 final		08.05.2018	AHE15PA	*Tenacibaculum*	Gundel_1	Gundel	78511
E42_11/2s/1 final		08.05.2018	AHE15PA	*Tenacibaculum*	Gundel_1	Gundel	78511
E42_11/3/1 final		08.05.2018	AHE15PA	*Tenacibaculum*	Gundel_1	Gundel	78511
E42_11/4/1 final		08.05.2018	AHE15PA	*Tenacibaculum*	Gundel_1	Gundel	78511
E42_11/5/1 final		08.05.2018	AHE15PA	*Tenacibaculum*	Gundel_1	Gundel	78511
E46_12/1/1 final	DSM111256	15.05.2018	HaHaR_3_96	*Olleya*	Harreka_1	Harreka	43175
E46_12/2/1 final		15.05.2018	HaHaR_3_96	*Olleya*	Harreka_1	Harreka	43175
E46_12/3/1 final		15.05.2018	HaHaR_3_96	*Olleya*	Harreka_1	Harreka	43175
E46_14/1/1 final		24.05.2018	HaHaR_3_96	*Olleya*	Harreka_1	Harreka	43175
E46_14/2/1 final		24.05.2018	HaHaR_3_96	*Olleya*	Harreka_1	Harreka	43175
E46_14/3/1 final		24.05.2018	HaHaR_3_96	*Olleya*	Harreka_1	Harreka	43175
E46_14/4/1 final		24.05.2018	HaHaR_3_96	*Olleya*	Harreka_1	Harreka	43175
4_2/1_II/2 final	DSM111236	21.03.2017	HaHaR_3_176	*Cellulophaga*	Ingeline_1	Ingeline	42624
4_2/1_II/4 final		21.03.2017	HaHaR_3_176	*Cellulophaga*	Ingeline_2	Ingeline	42624

4_2/1_II/1 final		21.03.2017	HaHaR_3_176	*Cellulophaga*	Ingeline_3	Ingeline	42624
4_2/2/2 final		21.03.2017	HaHaR_3_176	*Cellulophaga*	Ingeline_3	Ingeline	42624
4_2/2_II/1 final		21.03.2017	HaHaR_3_176	*Cellulophaga*	Ingeline_4	Ingeline	42624
4_2/3/3 final		21.03.2017	HaHaR_3_176	*Cellulophaga*	Ingeline_5	Ingeline	42625
E4_10/2/1 final		03.05.2018	HaHaR_3_176	*Cellulophaga*	Ingeline_6	Ingeline	42625
4_2/5/1 final		21.03.2017	HaHaR_3_176	*Cellulophaga*	Ingeline_7	Ingeline	42625
E4_10GS/2/1 final		03.05.2018	HaHaR_3_176	*Cellulophaga*	Ingeline_7	Ingeline	42625
4_2/2/3 final		21.03.2017	HaHaR_3_176	*Cellulophaga*	Ingeline_8	Ingeline	42775
E4_10/3/1 final		03.05.2018	HaHaR_3_176	*Cellulophaga*	Ingeline_8	Ingeline	42776
E4_10GS/3/1 final		03.05.2018	HaHaR_3_176	*Cellulophaga*	Ingeline_9	Ingeline	42797
E50_14/1/1 final	DSM111238	24.05.2018	AHE13PA	*Polaribacter*	Leef_1	Leef	37547
E50_14/2/1 final		24.05.2018	AHE13PA	*Polaribacter*	Leef_1	Leef	37547
E50_14/3/1 final		24.05.2018	AHE13PA	*Polaribacter*	Leef_1	Leef	37547
8_5/3/1 final	DSM111257	11.04.2017	DSM18668	*Maribacter*	Molly_1	Molly	124695
8_5/3/2 final		11.04.2017	DSM18668	*Maribacter*	Molly_1	Molly	124695
8_5/6/1 final		11.04.2017	DSM18668	*Maribacter*	Molly_1	Molly	124695
8_5/7/1 final		11.04.2017	DSM18668	*Maribacter*	Molly_1	Molly	124695
8_5/7/2 final		11.04.2017	DSM18668	*Maribacter*	Molly_1	Molly	124695
8_5/1/2 final		11.04.2017	DSM18668	*Maribacter*	Molly_2	Molly	124695
8_5/6/2 final		11.04.2017	DSM18668	*Maribacter*	Molly_3	Molly	124695
8_4/4/1 final		04.04.2017	DSM18668	*Maribacter*	Molly_4	Molly	125038
8_4/5/1 final		04.04.2017	DSM18668	*Maribacter*	Molly_5	Molly	124898
8_4/5/2 final		04.04.2017	DSM18668	*Maribacter*	Molly_5	Molly	124898
8_4/3/2 final		04.04.2017	DSM18668	*Maribacter*	Molly_7	Molly	125344
8_5/8/1 final	DSM111252	11.04.2017	DSM18668	*Maribacter*	Colly_1	Molly	124169
8_5/8/2 final		11.04.2017	DSM18668	*Maribacter*	Colly_1	Molly	124169
SW26_10/1s/1	DSM111239	03.05.2018	HaHa_2_1	*Cellulophaga*	Nekkels_1	Nekkels	53385
E26_2/1/1/F		03.04.2018	HaHa_2_1	*Cellulophaga*	Nekkels_2	Nekkels	54332
E26_2/1/2/F		03.04.2018	HaHa_2_1	*Cellulophaga*	Nekkels_2	Nekkels	54332
4_1/1/1 final	DSM111240	14.03.2017	HaHaR_3_176	*Cellulophaga*	Omtje_1	Omtje	6558
4_1/1/2 final		14.03.2017	HaHaR_3_176	*Cellulophaga*	Omtje_1	Omtje	6558
4_1/2/1 final		14.03.2017	HaHaR_3_176	*Cellulophaga*	Omtje_1	Omtje	6558
4_1/2/2 final		14.03.2017	HaHaR_3_176	*Cellulophaga*	Omtje_1	Omtje	6558
4_1/3/1 final		14.03.2017	HaHaR_3_176	*Cellulophaga*	Omtje_1	Omtje	6558
4_1/3/2 final		14.03.2017	HaHaR_3_176	*Cellulophaga*	Omtje_1	Omtje	6558
4_1/4/1 final		14.03.2017	HaHaR_3_176	*Cellulophaga*	Omtje_1	Omtje	6558
4_1/4/2 final		14.03.2017	HaHaR_3_176	*Cellulophaga*	Omtje_1	Omtje	6558
4_2/2/1 final		21.03.2017	HaHaR_3_176	*Cellulophaga*	Omtje_2	Omtje	6558
4_2/1_II/3 final		21.03.2017	HaHaR_3_176	*Cellulophaga*	Omtje_3	Omtje	6558
4_2/3/1 final		21.03.2017	HaHaR_3_176	*Cellulophaga*	Omtje_3	Omtje	6558
4_2/3_II/2 final		21.03.2017	HaHaR_3_176	*Cellulophaga*	Omtje_3	Omtje	6558
4_2/4/1 final		21.03.2017	HaHaR_3_176	*Cellulophaga*	Omtje_3	Omtje	6558
4_2/4/2 final		21.03.2017	HaHaR_3_176	*Cellulophaga*	Omtje_3	Omtje	6558
4_2/6/1 final		21.03.2017	HaHaR_3_176	*Cellulophaga*	Omtje_3	Omtje	6558
E4_10GS/1/1 final		03.05.2018	HaHaR_3_176	*Cellulophaga*	Omtje_3	Omtje	6558
E4_11/1/1 final		08.05.2018	HaHaR_3_176	*Cellulophaga*	Omtje_3	Omtje	6558
E4_11/2/1 final		08.05.2018	HaHaR_3_176	*Cellulophaga*	Omtje_3	Omtje	6558
E4_11/3/1 final		08.05.2018	HaHaR_3_176	*Cellulophaga*	Omtje_3	Omtje	6558
4_2/6/2 final		21.03.2017	HaHaR_3_176	*Cellulophaga*	Omtje_4	Omtje	6558
E4_10/1/1 final		03.05.2018	HaHaR_3_176	*Cellulophaga*	Omtje_5	Omtje	6559
4_2/3_II/1 final		21.03.2017	HaHaR_3_176	*Cellulophaga*	Omtje_6	Omtje	6558
E25_12/1s/1 final	DSM111241	15.05.2018	HaHa_3_26	*Winogradskyella*	Peternella_1	Peternella	39649
E25_12/1/1 final		15.05.2018	HaHa_3_26	*Winogradskyella*	Peternella_1	Peternella	39649
E25_12/2/1 final		15.05.2018	HaHa_3_26	*Winogradskyella*	Peternella_1	Peternella	39649
E25_12/3/1 final		15.05.2018	HaHa_3_26	*Winogradskyella*	Peternella_1	Peternella	39649

Table 6: Sampling site of metagenome derived contigs and their BLASTN bacterial hit. * indicated multiple hits.

Family	ID	Source	%identity	Start	End	Length	E-value	Bacteria_taxonomic_affiliation	Bacterium hit	Annotation of hit	
Forsetiviridae	GOV2_Station158_MES_NODE_1201	marine (Norwegian Sea)	68	38647	39569	922	2.76E-77	Flavobacteriaceae	Tenacibaculum sp. DSM 106434	integrase	*
**Forsetiviridae**	**GOV2_Station158_SUR_NODE_1364**	**marine (Norwegian Sea)**	**83**	**1**	**2399**	**2398**	**0**	***Flavobacteriaceae***	***Polaribacter vadi* strain LPB0003**	**C4-dicarboxylate ABC transporter**	*
**Forsetiviridae**	GOV2_Station76_MES_NODE_1002	marine (South Atlantic Ocean)	100	47019	47708	689	0	*Flavobacteriaceae*	*Maribacter cobaltidurans* strain B1	Paa1 family thioesterase	*
**Forsetiviridae**	GOV2_Station138_MES_NODE_1133	marine (North Pacific Ocean)	68	43953	45601	1648	8.68E-154	*Flavobacteriaceae*	*Elizabethkingia anophelis* strain E6809	hypothetical protein	*
**Pachyviridae**	IMGVR2_3300001278_____BBAY75_10000041	marine (macroalgae surface)	74	26456	26848	392	4.86E-57	*Flavobacteriaceae*	*Flavivirga eckloniae* strain ECD14	hypothetical protein	
**Pachyviridae**	IMGVR2_3300005056_____Ga0071102_1000080	marine (Atlantic Ocean)	75	54907	55242	335	3.45E-53	*Flavobacteriaceae*	*Formosa* sp. PS13	DUF3127	*
**Winoviridae**	IMGVR2_3300007093_____Ga0104055_1000085	human, oral	71	18546	19837	1291	1.1E-162	*Flavobacteriaceae*	*Capnocytophaga* sp. H2931	hypothetical protein	*
**Winoviridae**	IMGVR2_3300007713_____Ga0105659_1000065	human, oral	72	3	971	968	1.74E-147	*Flavobacteriaceae*	*Chryseobacterium balustinum* strain KC_1863	ribosome biogenesis GTPase Der	*
**Winoviridae**	IMGVR2_3300006459_____Ga0100222_100241	human, oral	70	33833	35432	1599	0	*Flavobacteriaceae*	*Chryseobacterium* sp. NBC122	hypothetical protein	*
**Winoviridae**	IMGVR2_3300007126_____Ga0102717_100371	human, oral	72	7	800	793	0	*Flavobacteriaceae*	*Elizabethkingia anophelis*	integrase	
**Winoviridae**	IMGVR2_3300008130_____Ga0114850_100310	human, oral	70	6522	10322	3800	0	*Flavobacteriaceae*	*Chryseobacterium* sp. F5649	2-oxoglutarate dehydrogenase complex	*

										dihydrolipoyllysine-residue succinyltransferase	
**Winoviridae**	IMGVR2_3300006742_____Ga0101805_100074	human, oral	81	3882	5124	1242	0	*Flavobacteriaceae*	*Chryseobacterium carnipullorum* strain F9942	ADP-forming succinate--CoA ligase subunit beta	*
**Winoviridae**	IMGVR2_3300012252_____Ga0122200_100146	city subway wood	74	800	3867	3067	0	*Flavobacteriaceae*	*Flavobacterium columnare* ATCC 49512	cytosine-specific methyltransferase	
**Winoviridae**	IMGVR2_3300010054_____Ga0098069_100157	marine (Subarctic Pacific Ocean)	68	195	1711	1516	2.03E-128	*Flavobacteriaceae*	*Flavobacterium columnare* strain 94-081	ABC transporter ATP-binding protein	*
**Winoviridae**	IMGVR2_3300019758_____Ga0193951_1000082	Freshwater microbial mat	89	1	1869	1868	0	*Flavobacteriaceae*	*Tenacibaculum mesophilum* strain DSM 13764	DUF4870	*
**Winoviridae**	IMGVR2_3300014204_____Ga0172381_10001225	landfill leachate	66	660	1247	587	3.57E-29	*Flavobacteriaceae*	*Chryseobacterium lactis* strain KC_1864	DNA adenine methylase	
**Helgolandviridae**	IMGVR2_3300001122_____JGI12148J13107_100002	marine (South Atlantic Ocean)	70	29050	29967	917	5.05E-105	*Flavobacteriaceae*	*Gillisia* sp. Hel1_33_143	hypothetical protein	*
**Helgolandviridae**	IMGVR2_3300012032_____Ga0136554_1000067	saline lake	91	29643	30564	921	0	*Flavobacteriaceae*	*Psychroflexus torquis* ATCC 700755	EndoU-type ribonuclease	*
**Helgolandviridae**	IMGVR2_3300009508_____Ga0115567_10000451	marine (Kabeltonne, North Sea)	94	28021	33865	5844	0	*Flavobacteriaceae*	*Gillisia* sp. Hel1_33_143	hypothetical protein	*
**Helgolandviridae**	GOV2_Station168_DCM_NODE_1833	marine (Barents Sea)	93	40662	41265	603	0	*Flavobacteriaceae*	*Polaribacter* sp. SA4-12	hypothetical protein	*
**Helgolandviridae**	IMGVR2_3300001605_____Draft_10001254	waste water	67	32641	34090	1449	1.58E-116	*Flavobacteriaceae*	*Tenacibaculum dicentrarchi* strain AY7486TD	hypothetical protein	*

**Helgolandviridae**	IMGVR2_3300005080_____Ga0069611_10000122	waste water	72	23590	24439	849	1E-119	*Flavobacteriaceae*	*Weeksella virosa* strain NCTC11634	site-specific DNA methylase	*
**Helgolandviridae**	IMGVR2_3300005080_____Ga0069611_10000213	waste water	71	17787	18276	489	6.43E-58	*Flavobacteriaceae*	*Chryseobacterium* sp. IHB B 17019	hypothetical protein	
**Helgolandviridae**	IMGVR2_3300015214_____Ga0172382_10001576	landfill leachate	69	31915	34020	2105	0	*Flavobacteriaceae*	*Chryseobacterium gleum* strain 3012STDY6944375	modification methylase DpnIIA	
**Duneviridae**	IMGVR2_3300008250_____Ga0105354_1000171	marine (Gulf of Mexico)	75	2531	4121	1590	0	*Flavobacteriaceae*	*Owenweeksia hongkongensis* DSM 17368	putative transcriptional regulator with HTH domain	*
**Winoviridae**	AP013511.1_Uncultured_Mediterranean_phage_uvMED,_group_G21,_isolate__uvMED-CGR-C117A-MedDCM-OCT-S32-C49	marine (Mediterranian Sea)	69	32267	32606	340	1E-24	*Flavobacteriales*	*Elizabethkingia anophelis* JUNP 353	hypothetical protein	*
**Pervagoviridae**	MN693163.1_Marine_virus_AFVG_25M177	marine (North Pacific Subtropical Gyre)	72	48199	48534	340	3E-36	*Flavobacteriales*	*Fluviicola taffensis* DSM 16823	RNA polymerase, sigma 70 subunit, RpoD subfamily	*
**Pervagoviridae**	MN693182.1_Marine_virus_AFVG_25M24	marine (North Pacific Subtropical Gyre)	95	5424	5497	74	5.51E-20	*Bacteroidetes*	*Ichthyobacterium seriolicida*	tRNA-Arg	*
**Pervagoviridae**	MN693201.1_Marine_virus_AFVG_25M427	marine (North Pacific Subtropical Gyre)	71	53839	54072	234	2E-18	*Firmicutes*	*Clostridium botulinum* B str. Eklund 17B	DNA methyltransferase	*
**Pervagoviridae**	MN693279.1_Marine_virus_AFVG_25M346	marine (North Pacific	93	10901	10975	75	7E-19	*Planctomycetes*	*Planctomycetes* bacterium K23_9	tRNA-Arg	*

		Subtropical Gyre)									
**Pervagoviridae**	MN693496.1_Marine_virus_AFVG_25M103	marine (North Pacific Subtropical Gyre)	-	-	-	-	-	-	-	-	-
**Pervagoviridae**	MN693779.1_Marine_virus_AFVG_250M346	marine (North Pacific Subtropical Gyre)	72	54677	55085	413	7E-38	*Firmicutes*	*Clostridioides difficile* strain FDAARGOS_723	replicative DNA helicase	
**Pervagoviridae**	MN582082.1_Podoviridae_sp._ctrTa16	freshwater	78	14179	14291	113	2E-12	*Firmicutes*	*Faecalibacterium prausnitzii* strain Indica	hypothetical protein	

Table 7: Spacer hits from IMG/VR blast against the viral spacer database and the metagenomic spacer database. When blasting against metagenomics spacer database only hits with one and zero mismatches were taken into account. (see Table 8).

Phage_name	Family_name	Spacer_BLAST/belongs to *Bacteroidetes*	Metagenome_SPACER_blast/belongs to *Bacteroidetes*
**Calle_1**	Pervagoviridae	0	0
**Colly_1**	Molycolviridae	0	0
**Danklef_1**	Forsetiviridae	0	0
**Freya_1**	Forsetiviridae	0	0
**Gundel_1**	Pachyviridae	0	0
**Harreka_1**	Aggregaviridae	0	0
**Ingeline_1**	Duneviridae	0	0
**Leef_1**	Helgolandviridae	0	0
**Molly_1**	Molycolviridae	0	0
**Nekkels_1**	Assiduviridae	0	8*/6
**Omtje_1**	Obscuriviridae	0	0
**Peternella_1**	Winoviridae	0	0
**KC821607.1_Cellulophaga_phage_phi19_1**	Assiduviridae	0	0
**KC821618.1_Cellulophaga_phage_phi10_1**	Assiduviridae	0	0
**MK764437.1_Flavobacterium_phage_FPSV-D15**	Duneviridae	104/104	1/0
**MK764440.1_Flavobacterium_phage_FPSV-F7**	Duneviridae	57/57	0
**MK764450.1_Flavobacterium_phage_FPSV-D35**	Duneviridae	99/99	1/0

**MN812211.1_Flavobacterium_phage_vB_FspS_laban6-1**	Duneviridae	0	0
**KC959568.1_Flavobacterium_phage_6H**	Duneviridae	77/77	1/0
**KU599887.1_Flavobacterium_phage_2A**	Duneviridae	69/69	1/0
**KU599888.1_Flavobacterium_phage_23T**	Duneviridae	72/72	0
**IMGVR2_3300008250_____Ga0105354_1000171**	Duneviridae	0	0
**GOV2_Station158_MES_ALL_assembly_NODE_1201**	Forsetiviridae	0	0
**GOV2_Station158_SUR_ALL_assembly_NODE_1364**	Forsetiviridae	0	0
**GOV2_Station76_MES_COMBINED_FINAL_NODE_1002**	Forsetiviridae	0	0
**GOV2_Station138_MES_COMBINED_FINAL_NODE_1133**	Forsetiviridae	0	0
**GOV2_Station168_DCM_ALL_assembly_NODE_1833**	Helgolandviridae	0	2/2
**IMGVR2_3300001122_____JGI12148J13107_100002**	Helgolandviridae	1/1	28/6
**IMGVR2_3300001605_____Draft_10001254**	Helgolandviridae	0	0
**IMGVR2_3300005080_____Ga0069611_10000122**	Helgolandviridae	0	0
**IMGVR2_3300005080_____Ga0069611_10000213**	Helgolandviridae	0	0
**IMGVR2_3300009508_____Ga0115567_10000451**	Helgolandviridae	0	0
**IMGVR2_3300012032_____Ga0136554_1000067**	Helgolandviridae	0	44/20
**IMGVR2_3300015214_____Ga0172382_10001576**	Helgolandviridae	0	0
**KC821608.1_Cellulophaga_phage_phi19_3**	Pachyviridae	0	0
**KC821620.1_Cellulophaga_phage_phi18_3**	Pachyviridae	0	0
**KC821622.1_Cellulophaga_phage_phi46_3**	Pachyviridae	0	0

**KC821633.1_Cellulophaga_phage_phi13_2**	Pachyviridae	0	0
**IMGVR2_3300001278_____BBAY75_10000041**	Pachyviridae	0	0
**IMGVR2_3300005056_____Ga0071102_1000080**	Pachyviridae	0	0
**MN582082.1_Podoviridae_sp._ctrTa16**	Pervagoviridae	0	0
**MN693163.1_Marine_virus_AFVG_25M177**	Pervagoviridae	0	0
**MN693182.1_Marine_virus_AFVG_25M24**	Pervagoviridae	0	0
**MN693201.1_Marine_virus_AFVG_25M427**	Pervagoviridae	0	0
**MN693279.1_Marine_virus_AFVG_25M346**	Pervagoviridae	0	0
**MN693496.1_Marine_virus_AFVG_25M103**	Pervagoviridae	0	0
**MN693779.1_Marine_virus_AFVG_250M346**	Pervagoviridae	0	0
**MT497123.1_Flavobacterium_phage_vB_Fsp_elemo8-9A**	Pervagoviridae	0	1/0
**KC821612.1_Cellulophaga_phage_phi40_1**	Pervagoviridae	0	0
**KC821614.1_Cellulophaga_phage_phi38_1**	Pervagoviridae	0	0
**IMGVR2_3300006459_____Ga0100222_100241**	Winoviridae	0	1/0
**IMGVR2_3300006742_____Ga0101805_100074**	Winoviridae	0	1/1
**IMGVR2_3300007093_____Ga0104055_1000085**	Winoviridae	0	1/0
**IMGVR2_3300007126_____Ga0102717_100371**	Winoviridae	0	1/0
**IMGVR2_3300007713_____Ga0105659_1000065**	Winoviridae	0	3/2
**IMGVR2_3300008130_____Ga0114850_100310**	Winoviridae	0	5/2
**IMGVR2_3300010054_____Ga0098069_100157**	Winoviridae	0	0

**IMGVR2_3300012252_____Ga0122200_100146**	Winoviridae	0	0
**IMGVR2_3300014204_____Ga0172381_10001225**	Winoviridae	2/0	7/2
**IMGVR2_3300019758_____Ga0193951_1000082**	Winoviridae	0	9/2
**AP013511.1_Uncultured_Mediterranean_phage_uvMED,_group_G21,_isolate__uvMED-CGR-C117A-MedDCM-OCT-S32-C49**	Winoviridae	0	0
**MK764442.1_Flavobacterium_phage_FPSV-S1**	Winoviridae	0	0
**MN812203.1_Flavobacterium_phage_vB_FspM_lotta8-1**	Winoviridae	0	0
**MN812204.1_Flavobacterium_phage_vB_FspM_lotta8-2**	Winoviridae	0	0
**MN812205.1_Flavobacterium_phage_vB_FspM_pippi8-1**	Winoviridae	0	0
**MN850656.1_Flavobacterium_phage_fF4**	Winoviridae	1/1	0
**MW421582.1_ Flavobacterium_phage_FPSV-S8**	Winoviridae	0	0

*** the viral spacer IDs: 3300027009:Ga0209093_1001256:1:1555, 3300027262:Ga0209303_1000810:1:15572, 3300027498:Ga0209185_1193139:1:249, 3300009417:Ga0114953_1000198:1:2830, 3300009421:Ga0114952_1377632:1:220, 3300009415:Ga0115029_1000158:2:63503, 3300009072:Ga0115030_1000872:1:653, 3300027325:Ga0209186_1000073:1:10669**

Table 8: IMG/VR Spacer blast to phages in families in detail.

Query Sequence Name	Query Start Coord	Query End Coord	Bit Score	E-value	Identities	Subject Length	Mismatches	Habitat	Host phylum
Colly_1	7895	7942	60.8	7.00E-06	88	50	6	freshwater, sediment	
Nekkels_1	2795	2830	60.8	3.00E-06	97	36	1	marine rhodophyta (red algae), host-associated, algal blade	Bacteroidetes
Nekkels_1	2795	2830	60.8	3.00E-06	97	36	1	marine rhodophyta (red algae), host-associated, algal blade	Bacteroidetes
Nekkels_1	2795	2830	60.8	3.00E-06	97	36	1	marine rhodophyta (red algae), host-associated, algal blade	
Nekkels_1	2795	2830	60.8	3.00E-06	97	36	1	marine rhodophyta (red algae), host-associated, algal blade, Porphyra	Bacteroidetes
Nekkels_1	2795	2830	60.8	3.00E-06	97	36	1	marine rhodophyta (red algae), host-associated, algal blade, Porphyra	
Nekkels_1	2795	2830	60.8	3.00E-06	97	36	1	marine rhodophyta (red algae), host-associated, algal blade, Porphyra	Bacteroidetes
Nekkels_1	2795	2830	60.8	3.00E-06	97	36	1	marine rhodophyta (red algae), host-associated, algal blade, Porphyra	Bacteroidetes
Nekkels_1	2795	2830	60.8	3.00E-06	97	36	1	marine rhodophyta (red algae), host-associated, algal blade, Porphyra	Bacteroidetes
GOV2_Station158_SUR_ALL_NODE_1364	49665	49711	59	9.00E-06	89	49	5	Capra hircus, goat feces, digestive system	Firmicutes
GOV2_Station168_DCM_ALL_NODE_1833	1513	1548	66.2	5.00E-08	100	37	0	saline lake, Non-marine Saline and Alkaline	Bacteroidetes
GOV2_Station168_DCM_ALL_NODE_1833	1513	1548	66.2	5.00E-08	100	37	0	saline lake, Non-marine Saline and Alkaline	Bacteroidetes
IMGVR2_3300001122_____JGI12148J13107_100002	22735	22770	60.8	2.00E-07	97	36	1	marine	Bacteroidetes
IMGVR2_3300001122_____JGI12148J13107_100002	30843	30878	66.2	5.00E-08	100	36	0	marine, Mediterranean Sea	
IMGVR2_3300001122_____JGI12148J13107_100002	1559	1595	62.6	7.00E-07	97	37	1	marine	
IMGVR2_3300001122_____JGI12148J13107_100002	1559	1595	62.6	7.00E-07	97	37	1	marine, TARA_142	
IMGVR2_3300001122_____JGI12148J13107_100002	22735	22770	60.8	2.00E-06	97	36	1	marine, TARA_146	Bacteroidetes
IMGVR2_3300001122_____JGI12148J13107_100002	22735	22770	60.8	2.00E-06	97	36	1	marine, TARA_056	Bacteroidetes
IMGVR2_3300001122_____JGI12148J13107_100002	22735	22770	60.8	2.00E-06	97	36	1	marine, TARA_065	
IMGVR2_3300001122_____JGI12148J13107_100002	22735	22770	60.8	2.00E-06	97	36	1	marine, TARA_122	

IMGVR2_3300001122_____JGI12148J13107_100002	22735	22770	60.8	2.00E-06	97	36	1	marine, TARA_112	Bacteroidetes
IMGVR2_3300001122_____JGI12148J13107_100002	22735	22770	60.8	2.00E-06	97	36	1	marine, TARA_122	
IMGVR2_3300001122_____JGI12148J13107_100002	22735	22770	60.8	2.00E-06	97	36	1	marine, TARA_102	
IMGVR2_3300001122_____JGI12148J13107_100002	22735	22770	60.8	2.00E-06	97	36	1	marine, TARA_109	
IMGVR2_3300001122_____JGI12148J13107_100002	22735	22770	60.8	2.00E-06	97	36	1	marine, TARA_142	
IMGVR2_3300001122_____JGI12148J13107_100002	22735	22770	60.8	2.00E-06	97	36	1	marine, TARA_148b	Bacteroidetes
IMGVR2_3300001122_____JGI12148J13107_100002	22735	22770	60.8	2.00E-06	97	36	1	marine, TARA_109	
IMGVR2_3300001122_____JGI12148J13107_100002	22735	22770	60.8	2.00E-06	97	36	1	marine, TARA_125	
IMGVR2_3300001122_____JGI12148J13107_100002	22735	22770	60.8	2.00E-06	97	36	1	marine, TARA_112	Bacteroidetes
IMGVR2_3300001122_____JGI12148J13107_100002	22735	22770	60.8	2.00E-06	97	36	1	marine, TARA_149	
IMGVR2_3300001122_____JGI12148J13107_100002	22735	22770	60.8	2.00E-06	97	36	1	marine, TARA_142	
IMGVR2_3300001122_____JGI12148J13107_100002	22735	22770	60.8	2.00E-06	97	36	1	marine, TARA_111	
IMGVR2_3300001122_____JGI12148J13107_100002	22735	22770	60.8	2.00E-06	97	36	1	marine, TARA_152	
IMGVR2_3300001122_____JGI12148J13107_100002	22735	22770	60.8	2.00E-06	97	36	1	marine, TARA_128	
IMGVR2_3300001122_____JGI12148J13107_100002	22735	22770	60.8	2.00E-06	97	36	1	marine, TARA_122	
IMGVR2_3300001122_____JGI12148J13107_100002	22735	22770	60.8	2.00E-06	97	36	1	marine, TARA_122	
IMGVR2_3300001122_____JGI12148J13107_100002	22735	22770	60.8	2.00E-06	97	36	1	marine, TARA_145	
IMGVR2_3300001122_____JGI12148J13107_100002	22735	22770	60.8	2.00E-06	97	36	1	marine, TARA_122	
IMGVR2_3300001122_____JGI12148J13107_100002	22735	22770	60.8	2.00E-06	97	36	1	marine, TARA_111	Bacteroidetes
IMGVR2_3300001122_____JGI12148J13107_100002	22735	22770	60.8	2.00E-06	97	36	1	marine, TARA_093	
IMGVR2_3300001122_____JGI12148J13107_100002	22735	22770	60.8	2.00E-06	97	36	1	marine, TARA_110	
IMGVR2_3300012032_____Ga0136554_1000067	10765	10801	68	1.00E-08	100	39	0	Non-marine Saline and Alkaline, saline lake	

IMGVR2_3300012032_____Ga0136554_1000067	10765	10801	68	1.00E-08	100	39	0	Non-marine Saline and Alkaline, saline lake	Bacteroidetes
IMGVR2_3300012032_____Ga0136554_1000067	10765	10801	68	1.00E-08	100	37	0	Non-marine Saline and Alkaline, saline lake	
IMGVR2_3300012032_____Ga0136554_1000067	10765	10801	68	1.00E-08	100	39	0	Non-marine Saline and Alkaline, saline lake	Bacteroidetes
IMGVR2_3300012032_____Ga0136554_1000067	10765	10801	68	1.00E-08	100	39	0	Non-marine Saline and Alkaline, saline lake	Bacteroidetes
IMGVR2_3300012032_____Ga0136554_1000067	10765	10801	68	1.00E-08	100	39	0	Non-marine Saline and Alkaline, saline lake	Bacteroidetes
IMGVR2_3300012032_____Ga0136554_1000067	10765	10801	68	1.00E-08	100	37	0	Non-marine Saline and Alkaline, saline lake	
IMGVR2_3300012032_____Ga0136554_1000067	10765	10801	68	1.00E-08	100	37	0	Non-marine Saline and Alkaline, saline lake	
IMGVR2_3300012032_____Ga0136554_1000067	10765	10801	68	1.00E-08	100	39	0	Non-marine Saline and Alkaline, saline lake	Bacteroidetes
IMGVR2_3300012032_____Ga0136554_1000067	10765	10801	68	1.00E-08	100	39	0	Non-marine Saline and Alkaline, saline lake	Bacteroidetes
IMGVR2_3300012032_____Ga0136554_1000067	10765	10801	68	1.00E-08	100	39	0	Non-marine Saline and Alkaline, saline lake	Bacteroidetes
IMGVR2_3300012032_____Ga0136554_1000067	10765	10801	68	1.00E-08	100	37	0	Non-marine Saline and Alkaline, saline lake	Bacteroidetes
IMGVR2_3300012032_____Ga0136554_1000067	10765	10801	68	1.00E-08	100	37	0	Non-marine Saline and Alkaline, saline lake	
IMGVR2_3300012032_____Ga0136554_1000067	10765	10801	68	1.00E-08	100	39	0	Non-marine Saline and Alkaline, saline lake	Bacteroidetes
IMGVR2_3300012032_____Ga0136554_1000067	10765	10801	68	1.00E-08	100	39	0	Non-marine Saline and Alkaline, saline lake	
IMGVR2_3300012032_____Ga0136554_1000067	10765	10801	68	1.00E-08	100	37	0	Non-marine Saline and Alkaline, saline lake	
IMGVR2_3300012032_____Ga0136554_1000067	10765	10801	68	1.00E-08	100	39	0	Non-marine Saline and Alkaline, saline lake	Bacteroidetes
IMGVR2_3300012032_____Ga0136554_1000067	10765	10801	68	1.00E-08	100	39	0	Non-marine Saline and Alkaline, saline lake	Bacteroidetes
IMGVR2_3300012032_____Ga0136554_1000067	10765	10801	68	1.00E-08	100	37	0	Non-marine Saline and Alkaline, saline lake	
IMGVR2_3300012032_____Ga0136554_1000067	10765	10801	68	1.00E-08	100	39	0	Non-marine Saline and Alkaline, saline lake	Bacteroidetes
IMGVR2_3300012032_____Ga0136554_1000067	10765	10801	68	1.00E-08	100	37	0	Non-marine Saline and Alkaline, saline lake	unavailable
IMGVR2_3300012032_____Ga0136554_1000067	10765	10801	68	1.00E-08	100	39	0	Non-marine Saline and Alkaline, saline lake	Bacteroidetes
IMGVR2_3300012032_____Ga0136554_1000067	10765	10801	68	1.00E-08	100	37	0	Non-marine Saline and Alkaline, saline lake	Bacteroidetes

IMGVR2_3300012032_____Ga0136554_1000067	10765	10801	68	1.00E-08	100	39	0	Non-marine Saline and Alkaline, saline lake	Bacteroidetes
IMGVR2_3300012032_____Ga0136554_1000067	10765	10801	68	1.00E-08	100	39	0	Non-marine Saline and Alkaline, saline lake	Bacteroidetes
IMGVR2_3300012032_____Ga0136554_1000067	10765	10801	68	1.00E-08	100	37	0	Non-marine Saline and Alkaline, saline lake	
IMGVR2_3300012032_____Ga0136554_1000067	10765	10801	68	1.00E-08	100	37	0	Non-marine Saline and Alkaline, saline lake	
IMGVR2_3300012032_____Ga0136554_1000067	10765	10801	68	1.00E-08	100	37	0	Non-marine Saline and Alkaline, saline lake	
IMGVR2_3300012032_____Ga0136554_1000067	10765	10801	68	1.00E-08	100	39	0	Non-marine Saline and Alkaline, saline lake	Bacteroidetes
IMGVR2_3300012032_____Ga0136554_1000067	10765	10801	68	1.00E-08	100	37	0	Non-marine Saline and Alkaline, saline lake	
IMGVR2_3300012032_____Ga0136554_1000067	10765	10801	68	1.00E-08	100	39	0	Non-marine Saline and Alkaline, saline lake	Bacteroidetes
IMGVR2_3300012032_____Ga0136554_1000067	10765	10801	68	1.00E-08	100	39	0	Non-marine Saline and Alkaline, saline lake	Bacteroidetes
IMGVR2_3300012032_____Ga0136554_1000067	10765	10801	68	1.00E-08	100	39	0	Non-marine Saline and Alkaline, saline lake	Bacteroidetes
IMGVR2_3300012032_____Ga0136554_1000067	10765	10800	66.2	5.00E-08	100	36	0	Non-marine Saline and Alkaline, saline lake	
IMGVR2_3300012032_____Ga0136554_1000067	10765	10800	66.2	5.00E-08	100	36	0	Non-marine Saline and Alkaline, saline lake	
IMGVR2_3300012032_____Ga0136554_1000067	34152	34187	66.2	5.00E-08	100	36	0	Non-marine Saline and Alkaline, saline lake	
IMGVR2_3300012032_____Ga0136554_1000067	34152	34187	66.2	5.00E-08	100	36	0	Non-marine Saline and Alkaline, saline lake	
IMGVR2_3300012032_____Ga0136554_1000067	34152	34187	66.2	5.00E-08	100	36	0	Non-marine Saline and Alkaline, saline lake	
IMGVR2_3300012032_____Ga0136554_1000067	34152	34187	66.2	5.00E-08	100	36	0	Non-marine Saline and Alkaline, saline lake	
IMGVR2_3300012032_____Ga0136554_1000067	26021	26057	62.6	6.00E-07	97	37	1	Non-marine Saline and Alkaline, saline lake	
IMGVR2_3300012032_____Ga0136554_1000067	26021	26057	62.6	6.00E-07	97	37	1	Non-marine Saline and Alkaline, saline lake	
IMGVR2_3300012032_____Ga0136554_1000067	26021	26057	62.6	6.00E-07	97	37	1	Non-marine Saline and Alkaline, saline lake	
IMGVR2_3300012032_____Ga0136554_1000067	26021	26057	62.6	6.00E-07	97	37	1	Non-marine Saline and Alkaline, saline lake	
IMGVR2_3300012032_____Ga0136554_1000067	26022	26057	60.8	2.00E-06	97	36	1	Non-marine Saline and Alkaline, saline lake	
IMGVR2_3300006459_____Ga0100222_100241	26194	26228	59	9.00E-06	97	35	1	oral cavity, human, digestive system	

IMGVR2_3300006742_____Ga0101805_100074	19751	19785	59	7.00E-06	97	36	1	oral cavity, human, digestive system	Bacteroidetes
IMGVR2_3300007093_____Ga0104055_1000085	6262	6296	59	9.00E-06	97	35	1	oral cavity, human, digestive system	
IMGVR2_3300007126_____Ga0102717_100371	29736	29770	59	9.00E-06	97	35	1	oral cavity, human, digestive system	
IMGVR2_3300007713_____Ga0105659_1000065	7286	7320	59	9.00E-06	97	35	1	oral cavity, human, digestive system	
IMGVR2_3300007713_____Ga0105659_1000065	28111	28145	59	9.00E-06	97	35	1	oral cavity, human, digestive system	Bacteroidetes
IMGVR2_3300007713_____Ga0105659_1000065	28111	28145	59	9.00E-06	97	35	1	oral cavity, human, digestive system	Bacteroidetes
IMGVR2_3300008130_____Ga0114850_100310	34251	34285	64.4	2.00E-07	100	36	0	oral cavity, human, digestive system	Bacteroidetes
IMGVR2_3300008130_____Ga0114850_100310	21375	21410	60.8	2.00E-06	97	36	1	oral cavity, human, digestive system	Bacteroidetes
IMGVR2_3300008130_____Ga0114850_100310	21375	21410	60.8	2.00E-06	97	36	1	oral cavity, human, digestive system	
IMGVR2_3300008130_____Ga0114850_100310	42830	42861	59	8.00E-06	100	35	0	oral cavity, human, digestive system	
IMGVR2_3300008130_____Ga0114850_100310	21802	21836	59	8.00E-06	97	35	1	oral cavity, human, digestive system	
IMGVR2_3300014204_____Ga0172381_10001225	24844	24880	59	4.00E-07	95	38	2		Proteobacteria
IMGVR2_3300014204_____Ga0172381_10001225	24843	24875	55.4	5.00E-06	97	38	1		Proteobacteria
IMGVR2_3300014204_____Ga0172381_10001225	3315	3352	69.8	4.00E-09	100	38	0	leachate well, landfill, solid waste	
IMGVR2_3300014204_____Ga0172381_10001225	19063	19098	66.2	5.00E-08	100	36	0	leachate well, landfill, solid waste	Bacteroidetes
IMGVR2_3300014204_____Ga0172381_10001225	2058	2093	66.2	5.00E-08	100	36	0	leachate well, landfill, solid waste	
IMGVR2_3300014204_____Ga0172381_10001225	30106	30141	66.2	5.00E-08	100	36	0	leachate well, landfill, solid waste	
IMGVR2_3300014204_____Ga0172381_10001225	20977	21010	62.6	7.00E-07	100	34	0	leachate well, landfill, solid waste	
IMGVR2_3300014204_____Ga0172381_10001225	23196	23229	62.6	7.00E-07	100	34	0	leachate well, landfill, solid waste	Bacteroidetes
IMGVR2_3300014204_____Ga0172381_10001225	17487	17524	60.8	2.00E-06	95	38	2	leachate well, landfill, solid waste	unavailable
IMGVR2_3300014204_____Ga0172381_10001225	17487	17522	60.8	2.00E-06	97	36	1	leachate well, landfill, solid waste	
IMGVR2_3300014204_____Ga0172381_10001225	3123	3157	59	8.00E-06	97	35	1	Anaerobic digestor sludge, wastewater	

MN693163.1_Marine_virus_AFVG_25 M177	10871	10925	68	3.00E -08	87	55	7	freshwaer, river	
MN693163.1_Marine_virus_AFVG_25 M177	54288	54336	75.2	2.00E -10	94	49	3	soil, wetland	
MN693163.1_Marine_virus_AFVG_25 M177	10871	10918	60.8	4.00E -06	88	57	7	freshwater, lake, epilimnion	
MN693182.1_Marine_virus_AFVG_25 M24	5434	5469	60.8	4.00E -06	97	81	2	Wastewater effluent	
MN693182.1_Marine_virus_AFVG_25 M24	5434	5469	60.8	4.00E -06	97	81	2	Wastewater effluent	
MN693182.1_Marine_virus_AFVG_25 M24	5436	5484	66.2	9.00E -08	90	49	5	soil, wetland	
MN693201.1_Marine_virus_AFVG_25 M427	43184	43231	64.4	3.00E -07	90	54	5	Macroalgal surface, seawater, botany bay, Australia	Haptista (Eukaryote)
MN693201.1_Marine_virus_AFVG_25 M427	43216	43264	62.6	1.00E -06	88	54	6	Subarctic Pacific Ocean	
MN693201.1_Marine_virus_AFVG_25 M427	43209	43258	64.4	3.00E -07	88	51	6		Haptista (Eukaryote)
MN693201.1_Marine_virus_AFVG_25 M427	43210	43264	64.4	3.00E -07	85	55	8	surface sea water	Bacteroidetes
MN693201.1_Marine_virus_AFVG_25 M427	43238	43283	60.8	4.00E -06	89	54	6		
MN693201.1_Marine_virus_AFVG_25 M427	43222	43324	60.8	4.00E -06	73	109	29		
MN693201.1_Marine_virus_AFVG_25 M427	43184	43231	69.8	7.00E -09	92	54	4		
MN693496.1_Marine_virus_AFVG_25 M103	44461	44507	71.6	2.00E -09	94	55	3		Haptista (Eukaryote)
MN693496.1_Marine_virus_AFVG_25 M103	44461	44507	71.6	2.00E -09	94	55	3		
MN693496.1_Marine_virus_AFVG_25 M103	44461	44513	60.8	4.00E -06	85	55	8		Bacteroidetes
MN693496.1_Marine_virus_AFVG_25 M103	44443	44486	62.6	1.00E -06	91	54	5		
MN693496.1_Marine_virus_AFVG_25 M103	44461	44507	71.6	2.00E -09	94	55	3		
MN693496.1_Marine_virus_AFVG_25 M103	44461	44507	71.6	2.00E -09	94	55	3		
MN693496.1_Marine_virus_AFVG_25 M103	44377	44424	60.8	4.00E -06	88	55	7		
MN693496.1_Marine_virus_AFVG_25 M103	44461	44507	71.6	2.00E -09	94	55	3		
MT497123.1_Flavobacterium_phage_vB_Fsp_elemo8-9A	21778	21810	60.8	4.00E -06	100	54	0		

KC821620.1_Cellulophaga_phage_phi18_3	14951	14988	60.8	4.00E-06	95	39	2	
KC821622.1_Cellulophaga_phage_phi46_3	14443	14480	60.8	5.00E-06	95	38	2	Bacteroidetes
MK764437.1_Flavobacterium_phage_FPSV-D15	24373	24409	62.6	8.00E-07	97	37	1	
MK764450.1_Flavobacterium_phage_FPSV-D35	17203	17239	62.6	8.00E-07	97	37	1	
KC959568.1_Flavobacterium_phage_6H	36945	36981	62.6	8.00E-07	97	37	1	
KU599887.1_Flavobacterium_phage_2A	37788	37824	62.6	8.00E-07	97	37	1	

Table 9: WIsH results for all dsDNA phages with the GEM dataset as host database.

Phage	Phage family	Otu ID	LogLikelihood	Taxonomy of host
**Harreka_1**	Aggregaviridae	OTU-29678	-1.31876	Bacteria; Bacteroidetes; Flavobacteriia; Flavobacteriales; Flavobacteriaceae
**KC821607.1_Cellulophaga phage phi19_1**	Assiduviridae	OTU-29271	-1.31133	Bacteria; Bacteroidetes; Flavobacteriia; Flavobacteriales; Flavobacteriaceae; Tenacibaculum
**KC821618.1_Cellulophaga phage phi10_1**	Assiduviridae	OTU-34689	-1.31184	Bacteria; Bacteroidetes; Flavobacteriia; Flavobacteriales; Flavobacteriaceae; Tenacibaculum; unclassified Tenacibaculum
**Nekkels_1**	Assiduviridae	OTU-34689	-1.31162	Bacteria; Bacteroidetes; Flavobacteriia; Flavobacteriales; Flavobacteriaceae; Tenacibaculum; unclassified Tenacibaculum
**IMGVR2_3300008250_Ga0105354_1000171**	Duneviridae	OTU-40845	-1.31409	Bacteria; Bacteroidetes; Flavobacteriia; Flavobacteriales; Flavobacteriaceae; Kordia
**Ingeline_1**	Duneviridae	OTU-32853	-1.30209	Bacteria; Bacteroidetes; Flavobacteriia; Flavobacteriales; Flavobacteriaceae; Tenacibaculum
**KC959568.1_Flavobacterium phage 6H**	Duneviridae	OTU-45523	-1.30558	Bacteria; Bacteroidetes; Flavobacteriia; Flavobacteriales; Flavobacteriaceae; Flavobacterium
**KU599887.1_Flavobacterium phage 2A**	Duneviridae	OTU-45523	-1.30634	Bacteria; Bacteroidetes; Flavobacteriia; Flavobacteriales; Flavobacteriaceae; Flavobacterium
**MK764437.1_Flavobacterium_phage_FPSV-D15**	Duneviridae	OTU-30565	-130.652	Bacteria; Bacteroidota; Bacteroidia; Flavobacteriales; Flavobacteriaceae; Flavobacterium; Flavobacterium sp000813005
**MK764440.1_Flavobacterium_phage_FPSV-F7**	Duneviridae	OTU-45523	-130.918	Bacteria; Bacteroidota; Bacteroidia; Flavobacteriales; Flavobacteriaceae; Flavobacterium; Flavobacterium hydatis
**MK764450.1_Flavobacterium_phage_FPSV-D35**	Duneviridae	OTU-30565	-130.678	Bacteria; Bacteroidota; Bacteroidia; Flavobacteriales; Flavobacteriaceae; Flavobacterium; Flavobacterium sp000813005

**MN812211.1_Flavobacterium_phage_vB_FspS_laban6-1**	Duneviridae	OTU-29938	-129.766	Bacteria; Bacteroidota; Bacteroidia; Flavobacteriales; Flavobacteriaceae; Flavobacterium; Flavobacterium sp000967805
**NC_041859.1_Flavobacterium phage 23T**	Duneviridae	OTU-45523	-1.30566	Bacteria; Bacteroidetes; Flavobacteriia; Flavobacteriales; Flavobacteriaceae; Flavobacterium
**Danklef_1**	Forsetiviridae	OTU-34689	-1.27206	Bacteria; Bacteroidetes; Flavobacteriia; Flavobacteriales; Flavobacteriaceae; Tenacibaculum; unclassified Tenacibaculum
**Freya_1**	Forsetiviridae	OTU-28695	-1.27432	Bacteria; Bacteroidetes; Flavobacteriia; Flavobacteriales; Flavobacteriaceae; Polaribacter
**GOV2_Station138_MES_COMBINED_FINAL_NODE_1133**	Forsetiviridae	OTU-44723	-1.32366	Bacteria; Bacteroidetes; Flavobacteriia; Flavobacteriales; Flavobacteriaceae; Winogradskyella
**GOV2_Station158_MES_ALL_assembly_NODE_1201**	Forsetiviridae	OTU-33717	-1.27825	Bacteria; Bacteroidetes; Flavobacteriia; Flavobacteriales; Flavobacteriaceae; Polaribacter
**GOV2_Station158_SUR_ALL_assembly_NODE_1364**	Forsetiviridae	OTU-34689	-1.27436	Bacteria; Bacteroidetes; Flavobacteriia; Flavobacteriales; Flavobacteriaceae; Tenacibaculum; unclassified Tenacibaculum
**GOV2_Station76_MES_COMBINED_FINAL_NODE_1002**	Forsetiviridae	OTU-20048	-1.28155	Bacteria; Bacteroidetes; Flavobacteriia; Flavobacteriales; Flavobacteriaceae
**GOV2_Station168_DCM_ALL_assembly_NODE_1833**	Helgolandviridae	OTU-18259	-1.27982	Bacteria; Bacteroidetes; Flavobacteriia; Flavobacteriales; Flavobacteriaceae; Polaribacter
**IMGVR2_3300001122_JGI12148J13107_100002**	Helgolandviridae	OTU-17680	-1.325	Bacteria; Bacteroidota; Bacteroidia; Flavobacteriales; Flavobacteriaceae; Mesonia; Mesonia mobilis
**IMGVR2_3300001605_Draft_10001254**	Helgolandviridae	OTU-37678	-1.29676	Bacteria; Bacteroidetes; Flavobacteriia; Flavobacteriales; Flavobacteriaceae; Lutibacter; unclassified Lutibacter
**IMGVR2_3300005080_Ga0069611_10000122**	Helgolandviridae	OTU-4361	-1.29869	Bacteria; Bacteroidota; Bacteroidia; Flavobacteriales; Crocinitomicaceae; UBA2040;
**IMGVR2_3300005080_Ga0069611_10000213**	Helgolandviridae	OTU-12527	-1.30945	Bacteria; Bacteroidota; Bacteroidia; Flavobacteriales; Weeksellaceae; Empedobacter; Empedobacter falsenii

**IMGVR2_3300009508_Ga0115567_10000451**	Helgolandviridae	OTU-44183	-1.3274	Bacteria; Bacteroidetes; Flavobacteriia; Flavobacteriales; Flavobacteriaceae; Gillisia
**IMGVR2_3300012032_Ga0136554_1000067**	Helgolandviridae	OTU-2207	-1.32328	Bacteria; Bacteroidota; Bacteroidia; Flavobacteriales; Flavobacteriaceae; Psychroflexus;
**IMGVR2_3300015214_Ga0172382_10001576**	Helgolandviridae	OTU-23484	-1.34026	Bacteria; Bacteroidetes; Flavobacteriia; Flavobacteriales; Crocinitomicaceae; Fluviicola
**Leef_1**	Helgolandviridae	OTU-39008	-1.26233	Bacteria; Bacteroidetes; Flavobacteriia; Flavobacteriales; Flavobacteriaceae; Polaribacter
**Colly_1**	Molycolviridae	OTU-23493	-1.35566	Bacteria; Bacteroidetes; Flavobacteriia; Flavobacteriales; Flavobacteriaceae; Myroides
**Molly_1**	Molycolviridae	OTU-23493	-1.35534	Bacteria; Bacteroidetes; Flavobacteriia; Flavobacteriales; Flavobacteriaceae; Myroides
**Gundel_1**	Pachyviridae	OTU-34689	-1.2915	Bacteria; Bacteroidetes; Flavobacteriia; Flavobacteriales; Flavobacteriaceae; Tenacibaculum; unclassified Tenacibaculum
**IMGVR2_3300001278_BBAY75_10000041**	Pachyviridae	OTU-35776	-1.29951	Bacteria; Bacteroidetes; Flavobacteriia; Flavobacteriales; Flavobacteriaceae; Aquimarina
**IMGVR2_3300005056_Ga0071102_1000080**	Pachyviridae	OTU-38197	-1.32839	Bacteria; Bacteroidetes; Flavobacteriia; Flavobacteriales; Flavobacteriaceae; Tenacibaculum
**KC821608.1_Cellulophaga_phage_phi19_3**	Pachyviridae	OTU-35776	-131.754	Bacteria; Bacteroidota; Bacteroidia; Flavobacteriales; Flavobacteriaceae; Aquimarina; Aquimarina latercula
**KC821620.1_Cellulophaga_phage_phi18_3**	Pachyviridae	OTU-35776	-13.198	Bacteria; Bacteroidota; Bacteroidia; Flavobacteriales; Flavobacteriaceae; Aquimarina; Aquimarina latercula
**KC821622.1, Cellulophaga phage phi46_3**	Pachyviridae	OTU-35776	-1.31769	Bacteria; Bacteroidetes; Flavobacteriia; Flavobacteriales; Flavobacteriaceae; Aquimarina
**KC821633.1_Cellulophaga_phage_phi13_2**	Pachyviridae	OTU-35776	-131.941	Bacteria; Bacteroidota; Bacteroidia; Flavobacteriales; Flavobacteriaceae; Aquimarina; Aquimarina latercula

**Calle_1**	Pervagoviridae	OTU-23493	-1.36104	Bacteria; Bacteroidetes; Flavobacteriia; Flavobacteriales; Flavobacteriaceae; Myroides
**KC821612.1_Cellulophaga phage phi40_1**	Pervagoviridae	OTU-14582	-1.3603	Bacteria; Bacteroidota; Bacteroidia; Flavobacteriales; Flavobacteriaceae; Myroides; Myroides odoratimimus
**KC821614.1_Cellulophaga phage phi38_1**	Pervagoviridae	OTU-14582	-1.36017	Bacteria; Bacteroidota; Bacteroidia; Flavobacteriales; Flavobacteriaceae; Myroides; Myroides odoratimimus
**MN582082.1_Podoviridae_sp._ctrTa16**	Pervagoviridae	OTU-19123	-13.612	Bacteria; Firmicutes_A; Clostridia; Lachnospirales; Lachnospiraceae; Butyrivibrio_A; Butyrivibrio_A crossotus
**MN693163.1_Marine_virus_AFVG_25M177**	Pervagoviridae	OTU-23910	-138.013	Archaea; Nanoarchaeota; Nanoarchaeia; Pacearchaeales; ARS1160; ARS10; ARS10 sp002690445
**MN693182.1_Marine_virus_AFVG_25M24**	Pervagoviridae	OTU-23910	-138.142	Archaea; Nanoarchaeota; Nanoarchaeia; Pacearchaeales; ARS1160; ARS10; ARS10 sp002690445
**MN693201.1_Marine_virus_AFVG_25M427**	Pervagoviridae	OTU-23493	-134.921	Bacteria; Bacteroidota; Bacteroidia; Flavobacteriales; Flavobacteriaceae; Myroides; Myroides marinus
**MN693279.1_Marine_virus_AFVG_25M346**	Pervagoviridae	OTU-23910	-138.298	Archaea; Nanoarchaeota; Nanoarchaeia; Pacearchaeales; ARS1160; ARS10; ARS10 sp002690445
**MN693496.1_Marine_virus_AFVG_25M103**	Pervagoviridae	OTU-25198	-134.669	Bacteria; Bacteroidota; Bacteroidia; Flavobacteriales; Flavobacteriaceae; Aquimarina; Aquimarina aggregata
**MN693779.1_Marine_virus_AFVG_250M346**	Pervagoviridae	OTU-14576	-129.138	Bacteria; Firmicutes_A; Clostridia; Clostridiales; Clostridiaceae; Clostridium
**MT497123.1_Flavobacterium_phage_vB_Fsp_elemo8-9A**	Pervagoviridae	OTU-23610	-131.072	Bacteria; Bacteroidota; Bacteroidia; Cytophagales; Flammeovirgaceae; Flammeovirga
**AP013511.1_Uncultured_Mediterranean_phage_uvMED_G21_uvMED-CGR-C117A-MedDCM-OCT-S32-C49**	Winoviridae	OTU-37651	-137.033	Bacteria; Firmicutes; Bacilli; Bacillales; Bacillaceae_A; Bacillus_W; Bacillus_W soli
**IMGVR2_3300006459_Ga0100222_100241**	Winoviridae	OTU-2442	-1.31926	Bacteria; Bacteroidota; Bacteroidia; Flavobacteriales; Weeksellaceae; UBA7623

**IMGVR2_3300006742_Ga0101805_100074**	Winoviridae	OTU-2442	-1.3256	Bacteria; Bacteroidota; Bacteroidia; Flavobacteriales; Weeksellaceae; UBA7623
**IMGVR2_3300007093_Ga0104055_1000085**	Winoviridae	OTU-6037	-1.33031	Bacteria; Bacteroidota; Bacteroidia; Flavobacteriales; Weeksellaceae
**IMGVR2_3300007126_Ga0102717_100371**	Winoviridae	OTU-2442	-1.31653	Bacteria; Bacteroidota; Bacteroidia; Flavobacteriales; Weeksellaceae; UBA7623
**IMGVR2_3300007713_Ga0105659_1000065**	Winoviridae	OTU-2090	-1.3308	Bacteria; Bacteroidota; Bacteroidia; Flavobacteriales; Weeksellaceae
**IMGVR2_3300008130_Ga0114850_100310**	Winoviridae	OTU-5922	-1.31712	Bacteria; Bacteroidota; Bacteroidia; Flavobacteriales; Weeksellaceae
**IMGVR2_3300010054_Ga0098069_100157**	Winoviridae	OTU-39445	-1.36389	Bacteria; Bacteroidetes; Sphingobacteriia; Sphingobacteriales; Sphingobacteriaceae; Pedobacter; unclassified Pedobacter
**IMGVR2_3300012252_Ga0122200_100146**	Winoviridae	OTU-12671	-1.29325	Bacteria; Bacteroidota; Bacteroidia; Flavobacteriales; Weeksellaceae; Soonwooa
**IMGVR2_3300014204_Ga0172381_10001225**	Winoviridae	OTU-15497	-1.37279	Bacteria; Bacteroidota; Bacteroidia; Cytophagales; Cyclobacteriaceae
**IMGVR2_3300019758_Ga0193951_1000082**	Winoviridae	OTU-34689	-1.31244	Bacteria; Bacteroidetes; Flavobacteriia; Flavobacteriales; Flavobacteriaceae; Tenacibaculum; unclassified Tenacibaculum
**MK764442.1_Flavobacterium_phage_FPSV-S1**	Winoviridae	OTU-3342	-13.376	Bacteria; Firmicutes_A; Clostridia; 4C28d-15; CAG-1252
**MN812203.1_Flavobacterium_phage_vB_FspM_lotta8-1**	Winoviridae	OTU-25293	-132.968	Bacteria; Bacteroidota; Bacteroidia; Flavobacteriales; Flavobacteriaceae; Flavobacterium; Flavobacterium denitrificans
**MN812204.1_Flavobacterium_phage_vB_FspM_lotta8-2**	Winoviridae	OTU-25293	-132.985	Bacteria; Bacteroidota; Bacteroidia; Flavobacteriales; Flavobacteriaceae; Flavobacterium; Flavobacterium denitrificans
**MN812205.1_Flavobacterium_phage_vB_FspM_pippi8-1**	Winoviridae	OTU-25293	-133.054	Bacteria; Bacteroidota; Bacteroidia; Flavobacteriales; Flavobacteriaceae; Flavobacterium; Flavobacterium denitrificans

**MN850656.1_Flavobacterium_phage_fF4**	Winoviridae	OTU-765	-133.865	Bacteria; Bacteroidota; Bacteroidia; Flavobacteriales; Flavobacteriaceae; Flavobacterium
**MW421582.1_Flavobacterium_phage_FPSV-S8**	Winoviridae	OTU-3342	-133.747	Bacteria; Firmicutes_A; Clostridia; 4C28d-15; CAG-1252
**Peternella_1**	Winoviridae	OTU-32105	-1.32776	Bacteria; Bacteroidetes; Flavobacteriia; Flavobacteriales; Flavobacteriaceae

Table 10: Special genomic features of the "Helgolandviridae" and "Duneviridae".

Genome names	Family	Integrase	LuxR	AI-E2	BACON	HicA/B
**IMGVR2_3300001122_____JGI12148J13107_100002**	Helgolandviridae	-	+	-	+	-
**IMGVR2_3300012032_____Ga0136554_1000067**	Helgolandviridae	+	+	-	+	-
**IMGVR2_3300009508_____Ga0115567_10000451**	Helgolandviridae	-	+	+	-	-
**GOV2_Station168_DCM_ALL_assembly_NODE_1833**	Helgolandviridae	+	+	-	+	-
**Leef_1**	Helgolandviridae	+	+	-	+	-
**IMGVR2_3300001605_____Draft_10001254**	Helgolandviridae	+	+	-	-	-
**IMGVR2_3300005080_____Ga0069611_10000122**	Helgolandviridae	+	-	-	-	+
**IMGVR2_3300015214_____Ga0172382_10001576**	Helgolandviridae	+	+	-	-	-
**IMGVR2_3300005080_____Ga0069611_10000213**	Helgolandviridae	-	-	-	-	-
**Flavobacterium_phage_vB_FspS_laban6−1**	Duneviridae	+	+	-	-	-
**Flavobacterium_phage_FPSV−D15**	Duneviridae	+	+	-	-	-
**Flavobacterium_phage_FPSV−D35**	Duneviridae	+	+	-	-	-
**Flavobacterium_phage_FPSV−F7**	Duneviridae	+	-	-	-	-
**KU599887.1_Flavobacterium_phage_2A**	Duneviridae	+	+	-	-	-
**KC959568.1_Flavobacterium_phage_6H**	Duneviridae	+	+	-	-	-
**KU599889.1_Flavobacterium_phage_23T**	Duneviridae	+	+	-	-	-
**IMGVR2_3300008250_____Ga0105354_1000171**	Duneviridae	+	+	-	-	-
**Ingeline_1**	Duneviridae	+	+	-	+	-

Table 11: CRISPR spacers of bacterial host isolates were mapped on new flavophage genomes and mismatches were counted. For Calle_1, Harreka_1, Ingeline_1, Molly_1, and Omtje_1 no match was found.

Bacterium	Length of spacer	Danklef_1	Freya_1	Gundel_1	Leef_1	Nekkels_1	Peternella_1
HaHaR_3_91	30						9
HaHaR_3_91	30		3				
HaHaR_3_91	30	8	8				
HaHaR_3_91	30		0				
HaHaR_3_91	30		3				
HaHaR_3_91	30	9	9			9	
HaHaR_3_91	30			8			
R2A056_3_33	31	1	1				
R2A056_3_33	30	3					
R2A056_3_33	30	1					
R2A056_3_33	30	8			1		
R2A056_3_33	30			9			

Table 12: Read mapping results of phage Danklef and its host.

Danklef						host 95% identity					phage 70% identity					phage 100% identity				
metagenome	date	Julian Day	total reads	total bases	size fraction	genome coverage [%]	mapped reads	total bases reads	relative read abundance [%]	normalized coverage [%]	genome coverage [%]	mapped reads	total bases reads	relative read abundance [%]	normalized coverage [%]	genome coverage [%]	mapped reads	total bases reads	relative read abundance [%]	normalized coverage [%]
	19.03.2018	78	132647062	3.216E+10	0.2 -3 µm	9.43	4994	1188625	0.0038	0.0085	5.03	22	5334	0.0000	0.0035	0.84	2	499	0.0000	0.0003
	03.04.2018	93	130734852	3.094E+10	0.2 -3 µm	54.99	24581	5897035	0.0188	0.0440	36.11	165	39487	0.0001	0.0270	6.69	21	4745	0.0000	0.0033
	05.04.2018	95	130836262	3.131E+10	0.2 -3 µm	12.02	7086	1666568	0.0054	0.0123	5.75	20	4901	0.0000	0.0033	1.15	4	942	0.0000	0.0006
	10.04.2018	100	131511730	3.167E+10	0.2 -3 µm	13.20	6380	1513284	0.0049	0.0110	4.80	17	4254	0.0000	0.0028	1.34	4	994	0.0000	0.0007
	12.04.2018	102	137437816	3.31E+10	0.2 -3 µm	14.19	6571	1566388	0.0048	0.0109	5.68	35	8393	0.0000	0.0054	NA	0	0	0.0000	0.0000
	17.04.2018	108	134007214	3.216E+10	0.2 -3 µm	23.07	11848	2800784	0.0088	0.0201	6.68	28	6956	0.0000	0.0046	0.67	2	500	0.0000	0.0003
	19.04.2018	110	145428602	3.516E+10	0.2 -3 µm	2.49	5360	1262124	0.0037	0.0083	3.38	9	2210	0.0000	0.0013	NA	0	0	0.0000	0.0000
	24.04.2018	115	148226228	3.577E+10	0.2 -3 µm	9.04	6485	1536719	0.0044	0.0099	4.14	27	6038	0.0000	0.0036	1.80	6	1199	0.0000	0.0007
	26.04.2018	117	186004950	4.485E+10	0.2 -3 µm	17.38	14495	3445399	0.0078	0.0177	8.88	118	28607	0.0001	0.0135	0.53	1	251	0.0000	0.0001
	02.05.2018	123	172417734	4.171E+10	0.2 -3 µm	69.26	50034	11884495	0.0290	0.0658	29.52	285	67909	0.0002	0.0345	3.67	8	1944	0.0000	0.0010
	03.05.2018	124	175408924	4.239E+10	0.2 -3 µm	16.15	14667	3490138	0.0084	0.0190	5.90	81	19063	0.0000	0.0095	NA	0	0	0.0000	0.0000
	08.05.2018	129	157697368	3.823E+10	0.2 -3 µm	4.09	9794	2315843	0.0062	0.0140	3.93	70	16889	0.0000	0.0094	NA	0	0	0.0000	0.0000
	11.05.2018	134	174367208	4.196E+10	0.2 -3 µm	2.19	7828	1837712	0.0045	0.0101	2.19	40	9605	0.0000	0.0049	NA	0	0	0.0000	0.0000
	15.05.2018	136	167144802	4.036E+10	0.2 -3 µm	1.68	6884	1614687	0.0041	0.0092	2.76	31	7690	0.0000	0.0040	NA	0	0	0.0000	0.0000
	17.05.2018	142	178481766	4.291E+10	0.2 -3 µm	2.54	6735	1581558	0.0038	0.0085	2.46	39	9563	0.0000	0.0047	NA	0	0	0.0000	0.0000
	22.05.2018	143	162504646	3.904E+10	0.2 -3 µm	45.15	23120	5492681	0.0142	0.0325	2.65	34	8124	0.0000	0.0044	NA	0	0	0.0000	0.0000
	24.05.2018	145	159197066	3.831E+10	0.2 -3 µm	11.96	11956	2806414	0.0075	0.0169	2.35	20	4787	0.0000	0.0026	NA	0	0	0.0000	0.0000
	29.05.2018	150	171271466	4.143E+10	0.2 -3 µm	9.28	8498	2006371	0.0050	0.0112	3.34	35	8514	0.0000	0.0044	NA	0	0	0.0000	0.0000
	19.03.2018	78	408373164	9.731E+10	3-10 µm	27.12	12534	2990531	0.0031	0.0071	22.10	91	21556	0.0000	0.0047	1.90	7	1427	0.0000	0.0003
	12.04.2018	102	332059730	7.927E+10	3-10 µm	23.33	11040	2641373	0.0033	0.0077	21.81	91	21294	0.0000	0.0057	4.00	13	2844	0.0000	0.0008
	17.04.2018	108	420871688	9.615E+10	3-10 µm	24.59	16273	3816006	0.0039	0.0092	11.67	71	16859	0.0000	0.0037	2.35	11	2583	0.0000	0.0006
	26.04.2018	117	341246096	8.148E+10	3-10 µm	29.41	15328	3672789	0.0045	0.0104	24.53	158	36915	0.0000	0.0096	3.45	8	1907	0.0000	0.0005
	08.05.2018	129	390032052	8.751E+10	3-10 µm	2.66	4425	980248	0.0011	0.0026	9.37	50	11268	0.0000	0.0027	2.17	10	2089	0.0000	0.0005
	11.05.2018	134	346400002	8.256E+10	3-10 µm	2.02	2851	678199	0.0008	0.0019	19.02	109	25778	0.0000	0.0066	3.90	11	2662	0.0000	0.0007
	22.05.2018	143	322898796	7.6E+10	3-10 µm	50.62	78041	18478370	0.0242	0.0562	3.98	97	22638	0.0000	0.0063	0.53	5	1215	0.0000	0.0003

29.05.2018	150	361105792	8.634E+10	3-10 µm	9.71	5784	1380526	0.0016	0.0037	16.49	66	14788	0.0000	0.0036	4.06	15	3227	0.0000	0.0008
19.03.2018	78	229237742	5.467E+10	>10 µm	2.94	7886	1838420	0.0034	0.0078	6.65	26	5165	0.0000	0.0020	4.17	16	2936	0.0000	0.0011
12.04.2018	102	366243118	8.751E+10	>10 µm	89.55	221392	53121088	0.0604	0.1402	98.42	790340	186175735	0.2158	45.0849	85.36	180645	42088011	0.0493	10.1922
17.04.2018	108	349480760	8.224E+10	>10 µm	7.10	19387	4643716	0.0055	0.0130	17.20	92	21466	0.0000	0.0055	4.38	14	3222	0.0000	0.0008
26.04.2018	117	357085094	8.282E+10	>10 µm	84.44	93975	21978405	0.0263	0.0613	11.02	492	114141	0.0001	0.0292	0.56	4	524	0.0000	0.0001
08.05.2018	129	332293300	7.447E+10	>10 µm	10.03	10802	2379436	0.0033	0.0074	9.18	156	34633	0.0000	0.0099	3.40	16	3250	0.0000	0.0009
11.05.2018	134	340848252	8.026E+10	>10 µm	3.83	3193	758983	0.0009	0.0022	14.13	68	15466	0.0000	0.0041	3.02	11	2241	0.0000	0.0006
22.05.2018	143	416648526	9.496E+10	>10 µm	97.97	780276	181559855	0.1873	0.4416	67.39	628	145880	0.0002	0.0326	15.34	51	11736	0.0000	0.0026
29.05.2018	150	369290526	7.388E+10	>10 µm	46.66	56043	11208341	0.0152	0.0350	9.53	144	28872	0.0000	0.0083	3.22	19	4129	0.0000	0.0012

Table 13: Read mapping results of phage Freya and its host

Freya						host 95% identity					phage 70% identity					phage 100% identity				
metagenome	date	Julian Day	total reads	total bases	size fraction	genome coverage [%]	mapped reads	total bases reads	relative read abundance [%]	normalized coverage [%]	genome coverage [%]	mapped reads	total bases reads	relative read abundance [%]	normalized coverage [%]	genome coverage [%]	mapped reads	total bases reads	relative read abundance [%]	normalized coverage [%]
	19.03.2018	78	132647062	3.216E+10	0.2 -3 µm	9.77	5004	1190814	0.0038	0.0088	4.06	20	4802	0.0000	0.0034	NA	0	0	0.0000	0.0000
	03.04.2018	93	130734852	3.094E+10	0.2 -3 µm	56.31	24590	5899122	0.0188	0.0453	41.04	179	42572	0.0001	0.0313	15.56	46	10810	0.0000	0.0079
	05.04.2018	95	130836262	3.131E+10	0.2 -3 µm	12.44	7098	1668773	0.0054	0.0127	6.92	21	5163	0.0000	0.0037	1.81	4	954	0.0000	0.0007
	10.04.2018	100	131511730	3.167E+10	0.2 -3 µm	13.63	6386	1515156	0.0049	0.0114	5.47	17	4253	0.0000	0.0031	2.01	5	1245	0.0000	0.0009
	12.04.2018	102	137437816	3.31E+10	0.2 -3 µm	14.67	6574	1566772	0.0048	0.0112	5.69	34	8332	0.0000	0.0057	NA	0	0	0.0000	0.0000
	17.04.2018	108	134007214	3.216E+10	0.2 -3 µm	23.74	11865	2805163	0.0089	0.0207	5.53	26	6463	0.0000	0.0046	1.14	2	501	0.0000	0.0004
	19.04.2018	110	145428602	3.516E+10	0.2 -3 µm	2.52	5337	1256293	0.0037	0.0085	2.40	8	1974	0.0000	0.0013	0.55	1	243	0.0000	0.0002
	24.04.2018	115	148226228	3.577E+10	0.2 -3 µm	9.41	6514	1543024	0.0044	0.0103	4.28	34	7607	0.0000	0.0048	0.95	3	587	0.0000	0.0004
	26.04.2018	117	186004950	4.485E+10	0.2 -3 µm	17.92	14636	3480717	0.0079	0.0184	9.29	109	26337	0.0001	0.0134	3.19	7	1624	0.0000	0.0008
	02.05.2018	123	172417734	4.171E+10	0.2 -3 µm	70.63	49858	11843314	0.0289	0.0675	27.45	272	64448	0.0002	0.0351	9.76	24	5764	0.0000	0.0031
	03.05.2018	124	175408924	4.239E+10	0.2 -3 µm	16.87	14788	3519557	0.0084	0.0197	6.96	88	20730	0.0001	0.0111	0.86	2	498	0.0000	0.0003
	08.05.2018	129	157697368	3.823E+10	0.2 -3 µm	4.28	9831	2324291	0.0062	0.0144	3.65	59	14136	0.0000	0.0084	NA	0	0	0.0000	0.0000
	11.05.2018	134	174367208	4.196E+10	0.2 -3 µm	2.30	7838	1839997	0.0045	0.0104	2.36	45	10759	0.0000	0.0058	NA	0	0	0.0000	0.0000
	15.05.2018	136	167144802	4.036E+10	0.2 -3 µm	1.76	6901	1617856	0.0041	0.0095	1.88	32	7889	0.0000	0.0044	NA	0	0	0.0000	0.0000
	17.05.2018	142	178481766	4.291E+10	0.2 -3 µm	2.67	6766	1589226	0.0038	0.0088	2.64	36	8918	0.0000	0.0047	NA	0	0	0.0000	0.0000
	22.05.2018	143	162504646	3.904E+10	0.2 -3 µm	46.23	23108	5488630	0.0142	0.0334	2.18	31	7265	0.0000	0.0042	NA	0	0	0.0000	0.0000

	24.05.2018	145	159197066	3.831E+10	0.2 -3 µm	12.19	11947	2803060	0.0075	0.0174	2.52	17	4035	0.0000	0.0024	NA	0	0	0.0000	0.0000
	29.05.2018	150	171271466	4.143E+10	0.2 -3 µm	9.68	8528	2014164	0.0050	0.0116	4.15	36	8765	0.0000	0.0048	0.56	1	248	0.0000	0.0001
	19.03.2018	78	408373164	9.731E+10	3-10 µm	28.03	12535	2992590	0.0031	0.0073	23.33	87	20723	0.0000	0.0048	6.23	19	4286	0.0000	0.0010
	12.04.2018	102	332059730	7.927E+10	3-10 µm	23.93	11013	2630933	0.0033	0.0079	30.07	113	26470	0.0000	0.0076	20.53	62	14457	0.0000	0.0041
	17.04.2018	108	420871688	9.615E+10	3-10 µm	25.34	16327	3823644	0.0039	0.0094	15.33	89	21284	0.0000	0.0050	6.77	23	5412	0.0000	0.0013
	26.04.2018	117	341246096	8.148E+10	3-10 µm	29.92	15261	3654928	0.0045	0.0107	31.34	176	40872	0.0001	0.0114	21.11	65	14571	0.0000	0.0041
	08.05.2018	129	390032052	8.751E+10	3-10 µm	2.79	4626	1017508	0.0012	0.0028	11.30	70	15650	0.0000	0.0041	7.94	38	8572	0.0000	0.0022
	11.05.2018	134	346400002	8.256E+10	3-10 µm	2.15	3015	704747	0.0009	0.0020	25.64	131	31052	0.0000	0.0086	21.64	74	17316	0.0000	0.0048
	22.05.2018	143	322898796	7.6E+10	3-10 µm	51.84	77576	18356831	0.0240	0.0574	3.79	135	31736	0.0000	0.0095	1.02	9	2219	0.0000	0.0007
	29.05.2018	150	361105792	8.634E+10	3-10 µm	9.94	5781	1377963	0.0016	0.0038	19.02	77	17065	0.0000	0.0045	15.86	52	11517	0.0000	0.0030
	19.03.2018	78	229237742	5.467E+10	>10 µm	3.05	7871	1833101	0.0034	0.0080	5.03	44	9492	0.0000	0.0039	0.60	4	523	0.0000	0.0002
	12.04.2018	102	366243118	8.751E+10	>10 µm	91.68	221532	53163739	0.0605	0.1444	100.00	1017402	239788612	0.2778	62.3038	100.00	862986	203769042	0.2356	52.9449
	17.04.2018	108	349480760	8.224E+10	>10 µm	7.27	19439	4639441	0.0056	0.0134	25.67	128	29918	0.0000	0.0083	20.06	65	14670	0.0000	0.0041
	26.04.2018	117	357085094	8.282E+10	>10 µm	85.92	93420	21843750	0.0262	0.0627	8.02	511	119352	0.0001	0.0328	NA	0	0	0.0000	0.0000
	08.05.2018	129	332293300	7.447E+10	>10 µm	10.26	10703	2357850	0.0032	0.0075	10.20	151	33976	0.0000	0.0104	2.58	11	2309	0.0000	0.0007
	11.05.2018	134	340848252	8.026E+10	>10 µm	3.77	3202	755997	0.0009	0.0022	18.26	83	18751	0.0000	0.0053	15.61	52	11565	0.0000	0.0033
	22.05.2018	143	416648526	9.496E+10	>10 µm	97.83	777821	180995463	0.1867	0.4529	77.85	656	152642	0.0002	0.0365	38.06	137	31079	0.0000	0.0074
	29.05.2018	150	369290526	7.388E+10	>10 µm	47.44	55357	11074665	0.0150	0.0356	9.31	139	28185	0.0000	0.0087	5.23	30	6314	0.0000	0.0019

Table 14: Read mapping results of phage Harreka and its host

Harreka						host 95% identity					phage 70% identity					phage 100% identity				
metagenome	date	Julian Day	total reads	total bases	size fraction	genome coverage [%]	mapped reads	total bases reads	relative read abundance [%]	normalized coverage [%]	genome coverage [%]	mapped reads	total bases reads	relative read abundance [%]	normalized coverage [%]	genome coverage [%]	mapped reads	total bases reads	relative read abundance [%]	normalized coverage [%]
	19.03.2018	78	132647062	3.216E+10	0.2 -3 µm	5.69	4202	996396	0.0032	0.0070	4.50	12	2778	0.0000	0.0020	1.26	3	751	0.0000	0.0005
	03.04.2018	93	130734852	3.094E+10	0.2 -3 µm	26.98	12212	2905325	0.0093	0.0213	36.91	127	30850	0.0001	0.0231	0.98	2	501	0.0000	0.0004
	05.04.2018	95	130836262	3.131E+10	0.2 -3 µm	3.03	5394	1251525	0.0041	0.0091	0.79	2	501	0.0000	0.0004	NA	0	0	0.0000	0.0000
	10.04.2018	100	131511730	3.167E+10	0.2 -3 µm	4.50	4365	1026578	0.0033	0.0074	0.61	2	496	0.0000	0.0004	NA	0	0	0.0000	0.0000
	12.04.2018	102	137437816	3.31E+10	0.2 -3 µm	4.90	4379	1035734	0.0032	0.0071	1.61	4	1003	0.0000	0.0007	NA	0	0	0.0000	0.0000
	17.04.2018	108	134007214	3.216E+10	0.2 -3 µm	8.91	7223	1694746	0.0054	0.0119	0.96	2	416	0.0000	0.0003	NA	0	0	0.0000	0.0000
	19.04.2018	110	145428602	3.516E+10	0.2 -3 µm	0.94	5141	1208712	0.0035	0.0078	1.15	2	501	0.0000	0.0003	NA	0	0	0.0000	0.0000

24.04.2018	115	148226228	3.577E+10	0.2 -3 µm	4.55	5030	1187657	0.0034	0.0075	NA	0	0	0.0000	0.0000	NA	0	0	0.0000	0.0000
26.04.2018	117	186004950	4.485E+10	0.2 -3 µm	8.06	9685	2288183	0.0052	0.0116	2.22	4	957	0.0000	0.0005	NA	0	0	0.0000	0.0000
02.05.2018	123	172417734	4.171E+10	0.2 -3 µm	59.11	34669	8195312	0.0201	0.0446	11.15	29	7107	0.0000	0.0039	1.81	4	954	0.0000	0.0005
03.05.2018	124	175408924	4.239E+10	0.2 -3 µm	9.21	12262	2909762	0.0070	0.0156	2.25	8	1905	0.0000	0.0010	NA	0	0	0.0000	0.0000
08.05.2018	129	157697368	3.823E+10	0.2 -3 µm	2.25	11830	2793998	0.0075	0.0166	NA	0	0	0.0000	0.0000	NA	0	0	0.0000	0.0000
11.05.2018	134	174367208	4.196E+10	0.2 -3 µm	1.56	10251	2401621	0.0059	0.0130	NA	0	0	0.0000	0.0000	NA	0	0	0.0000	0.0000
15.05.2018	136	167144802	4.036E+10	0.2 -3 µm	1.24	9985	2346676	0.0060	0.0132	0.58	1	251	0.0000	0.0001	NA	0	0	0.0000	0.0000
17.05.2018	142	178481766	4.291E+10	0.2 -3 µm	1.74	8796	2062928	0.0049	0.0109	2.52	5	1236	0.0000	0.0007	NA	0	0	0.0000	0.0000
22.05.2018	143	162504646	3.904E+10	0.2 -3 µm	3.67	9578	2236473	0.0059	0.0130	1.16	2	502	0.0000	0.0003	NA	0	0	0.0000	0.0000
24.05.2018	145	159197066	3.831E+10	0.2 -3 µm	1.59	10564	2468194	0.0066	0.0146	1.41	5	974	0.0000	0.0006	NA	0	0	0.0000	0.0000
29.05.2018	150	171271466	4.143E+10	0.2 -3 µm	1.47	7214	1694820	0.0042	0.0093	0.99	5	997	0.0000	0.0006	NA	0	0	0.0000	0.0000
19.03.2018	78	408373164	9.731E+10	3-10 µm	11.63	7308	1740784	0.0018	0.0041	10.18	44	8804	0.0000	0.0021	1.98	6	1177	0.0000	0.0003
12.04.2018	102	332059730	7.927E+10	3-10 µm	6.23	4417	1057338	0.0013	0.0030	9.01	24	5829	0.0000	0.0017	0.58	1	251	0.0000	0.0001
17.04.2018	108	420871688	9.615E+10	3-10 µm	12.11	8315	1939600	0.0020	0.0046	8.15	36	7691	0.0000	0.0019	0.54	1	234	0.0000	0.0001
26.04.2018	117	341246096	8.148E+10	3-10 µm	20.38	9099	2186167	0.0027	0.0061	7.10	23	5755	0.0000	0.0016	NA	0	0	0.0000	0.0000
08.05.2018	129	390032052	8.751E+10	3-10 µm	1.62	4521	1015272	0.0012	0.0026	100.00	128332	21163852	0.0329	5.6013	100.00	41276	6833569	0.0106	1.8086
11.05.2018	134	346400002	8.256E+10	3-10 µm	1.72	4522	1081727	0.0013	0.0030	0.77	3	751	0.0000	0.0002	NA	0	0	0.0000	0.0000
22.05.2018	143	322898796	7.6E+10	3-10 µm	3.44	10539	2467150	0.0033	0.0074	1.98	9	2025	0.0000	0.0006	NA	0	0	0.0000	0.0000
29.05.2018	150	361105792	8.634E+10	3-10 µm	1.71	4993	1193566	0.0014	0.0031	1.96	4	958	0.0000	0.0003	NA	0	0	0.0000	0.0000
19.03.2018	78	229237742	5.467E+10	>10 µm	1.31	4438	1026359	0.0019	0.0043	1.16	28	6675	0.0000	0.0028	NA	0	0	0.0000	0.0000
12.04.2018	102	366243118	8.751E+10	>10 µm	58.53	71613	17163734	0.0196	0.0445	3.25	11	2390	0.0000	0.0006	0.32	2	275	0.0000	0.0001
17.04.2018	108	349480760	8.224E+10	>10 µm	3.90	11635	2777313	0.0033	0.0077	2.62	58	14207	0.0000	0.0040	NA	0	0	0.0000	0.0000
26.04.2018	117	357085094	8.282E+10	>10 µm	63.36	43395	10135769	0.0122	0.0278	10.76	35	7517	0.0000	0.0021	NA	0	0	0.0000	0.0000
08.05.2018	129	332293300	7.447E+10	>10 µm	6.60	7271	1600647	0.0022	0.0049	4.22	17	3648	0.0000	0.0011	NA	0	0	0.0000	0.0000
11.05.2018	134	340848252	8.026E+10	>10 µm	4.39	3577	858813	0.0010	0.0024	2.31	9	1564	0.0000	0.0005	0.36	2	312	0.0000	0.0001
22.05.2018	143	416648526	9.496E+10	>10 µm	44.62	30459	6982053	0.0073	0.0167	3.29	8	1841	0.0000	0.0004	NA	0	0	0.0000	0.0000
29.05.2018	150	369290526	7.388E+10	>10 µm	3.82	11398	2253368	0.0031	0.0069	2.66	35	6355	0.0000	0.0020	NA	0	0	0.0000	0.0000

Table 15: Read mapping results of phage Ingeline and its host

Ingeline						host 95% identity					phage 70% identity					phage 100% identity				
metagenome	date	Julian Day	total reads	total bases	size fraction	genome coverage [%]	mapped reads	total bases reads	relative read abundance [%]	normalized coverage [%]	genome coverage [%]	mapped reads	total bases reads	relative read abundance [%]	normalized coverage [%]	genome coverage [%]	mapped reads	total bases reads	relative read abundance [%]	normalized coverage [%]
	19.03.2018	78	132647062	3.216E+10	0.2 -3 µm	0.75	1806	421932	0.0014	0.0034	NA	0	0	0.0000	0.0000	NA	0	0	0.0000	0.0000
	03.04.2018	93	130734852	3.094E+10	0.2 -3 µm	6.63	3860	907066	0.0030	0.0077	2.26	5	1192	0.0000	0.0009	0.91	2	496	0.0000	0.0004
	05.04.2018	95	130836262	3.131E+10	0.2 -3 µm	0.61	2580	592503	0.0020	0.0050	NA	0	0	0.0000	0.0000	NA	0	0	0.0000	0.0000
	10.04.2018	100	131511730	3.167E+10	0.2 -3 µm	0.91	2373	551176	0.0018	0.0046	NA	0	0	0.0000	0.0000	NA	0	0	0.0000	0.0000
	12.04.2018	102	137437816	3.31E+10	0.2 -3 µm	0.72	2511	585454	0.0018	0.0046	NA	0	0	0.0000	0.0000	NA	0	0	0.0000	0.0000
	17.04.2018	108	134007214	3.216E+10	0.2 -3 µm	2.10	4162	959789	0.0031	0.0078	NA	0	0	0.0000	0.0000	NA	0	0	0.0000	0.0000
	19.04.2018	110	145428602	3.516E+10	0.2 -3 µm	0.32	3307	772074	0.0023	0.0058	NA	0	0	0.0000	0.0000	NA	0	0	0.0000	0.0000
	24.04.2018	115	148226228	3.577E+10	0.2 -3 µm	0.70	3271	760049	0.0022	0.0056	NA	0	0	0.0000	0.0000	NA	0	0	0.0000	0.0000
	26.04.2018	117	186004950	4.485E+10	0.2 -3 µm	0.93	5333	1250110	0.0029	0.0073	0.59	1	250	0.0000	0.0001	NA	0	0	0.0000	0.0000
	02.05.2018	123	172417734	4.171E+10	0.2 -3 µm	9.43	9073	2102765	0.0053	0.0133	3.76	7	1732	0.0000	0.0010	NA	0	0	0.0000	0.0000
	03.05.2018	124	175408924	4.239E+10	0.2 -3 µm	1.45	6294	1481953	0.0036	0.0092	0.36	1	155	0.0000	0.0001	NA	0	0	0.0000	0.0000
	08.05.2018	129	157697368	3.823E+10	0.2 -3 µm	0.62	6111	1442289	0.0039	0.0099	NA	0	0	0.0000	0.0000	NA	0	0	0.0000	0.0000
	11.05.2018	134	174367208	4.196E+10	0.2 -3 µm	0.29	5321	1237808	0.0031	0.0078	NA	0	0	0.0000	0.0000	NA	0	0	0.0000	0.0000
	15.05.2018	136	167144802	4.036E+10	0.2 -3 µm	0.30	4826	1124904	0.0029	0.0073	NA	0	0	0.0000	0.0000	NA	0	0	0.0000	0.0000
	17.05.2018	142	178481766	4.291E+10	0.2 -3 µm	0.39	4426	1034588	0.0025	0.0063	NA	0	0	0.0000	0.0000	NA	0	0	0.0000	0.0000
	22.05.2018	143	162504646	3.904E+10	0.2 -3 µm	0.79	5674	1314451	0.0035	0.0089	NA	0	0	0.0000	0.0000	NA	0	0	0.0000	0.0000
	24.05.2018	145	159197066	3.831E+10	0.2 -3 µm	0.43	7626	1773688	0.0048	0.0122	NA	0	0	0.0000	0.0000	NA	0	0	0.0000	0.0000
	29.05.2018	150	171271466	4.143E+10	0.2 -3 µm	0.39	5813	1358236	0.0034	0.0086	NA	0	0	0.0000	0.0000	NA	0	0	0.0000	0.0000
	19.03.2018	78	408373164	9.731E+10	3-10 µm	2.09	2374	553524	0.0006	0.0015	NA	0	0	0.0000	0.0000	NA	0	0	0.0000	0.0000
	12.04.2018	102	332059730	7.927E+10	3-10 µm	1.02	1452	343084	0.0004	0.0011	2.49	7	1603	0.0000	0.0005	1.42	3	720	0.0000	0.0002
	17.04.2018	108	420871688	9.615E+10	3-10 µm	1.78	1866	419786	0.0004	0.0011	0.59	5	1212	0.0000	0.0003	NA	0	0	0.0000	0.0000
	26.04.2018	117	341246096	8.148E+10	3-10 µm	1.95	1629	384084	0.0005	0.0012	1.06	7	1124	0.0000	0.0003	1.06	6	947	0.0000	0.0003
	08.05.2018	129	390032052	8.751E+10	3-10 µm	0.38	1730	375951	0.0004	0.0011	0.61	5	1177	0.0000	0.0003	NA	0	0	0.0000	0.0000
	11.05.2018	134	346400002	8.256E+10	3-10 µm	0.21	1616	383164	0.0005	0.0012	NA	0	0	0.0000	0.0000	NA	0	0	0.0000	0.0000
	22.05.2018	143	322898796	7.6E+10	3-10 µm	0.51	3232	740108	0.0010	0.0026	0.38	10	1840	0.0000	0.0006	NA	0	0	0.0000	0.0000
	29.05.2018	150	361105792	8.634E+10	3-10 µm	0.44	2210	520325	0.0006	0.0016	0.48	1	206	0.0000	0.0001	0.48	1	206	0.0000	0.0001

19.03.2018	78	229237742	5.467E+10	>10 µm	0.22	1449	327028	0.0006	0.0016	NA	0	0	0.0000	0.0000	NA	0	0	0.0000	0.0000
12.04.2018	102	366243118	8.751E+10	>10 µm	7.56	11438	2699344	0.0031	0.0081	99.99	12452	2975865	0.0034	0.7978	99.98	10893	2607306	0.0030	0.6990
17.04.2018	108	349480760	8.224E+10	>10 µm	0.72	3032	721762	0.0009	0.0023	NA	0	0	0.0000	0.0000	NA	0	0	0.0000	0.0000
26.04.2018	117	357085094	8.282E+10	>10 µm	6.39	6043	1370596	0.0017	0.0044	NA	0	0	0.0000	0.0000	NA	0	0	0.0000	0.0000
08.05.2018	129	332293300	7.447E+10	>10 µm	1.36	2474	526918	0.0007	0.0019	0.42	1	177	0.0000	0.0001	NA	0	0	0.0000	0.0000
11.05.2018	134	340848252	8.026E+10	>10 µm	0.41	1056	248855	0.0003	0.0008	1.52	4	1004	0.0000	0.0003	NA	0	0	0.0000	0.0000
22.05.2018	143	416648526	9.496E+10	>10 µm	4.35	5792	1292102	0.0014	0.0036	0.62	4	525	0.0000	0.0001	0.32	2	267	0.0000	0.0001
29.05.2018	150	369290526	7.388E+10	>10 µm	1.30	4502	872547	0.0012	0.0031	0.73	5	885	0.0000	0.0003	NA	0	0	0.0000	0.0000

Table 16: Read mapping results of phage Leef and its host

Leef metagenome	date	Julian Day	total reads	total bases	size fraction	phage 70% identity genome coverage [%]	mapped reads	total bases reads	relative read abundance [%]	normalized coverage [%]	phage 100% identity genome coverage [%]	mapped reads	total bases reads	relative read abundance [%]	normalized coverage [%]
	19.03.2018	78	132647062	3.216E+10	0.2 -3 µm	1.90	3	749	0.0000	0.0006	NA	0	0	0.0000	0.0000
	03.04.2018	93	130734852	3.094E+10	0.2 -3 µm	17.57	42	10062	0.0000	0.0087	3.33	8	1964	0.0000	0.0017
	05.04.2018	95	130836262	3.131E+10	0.2 -3 µm	2.04	4	1003	0.0000	0.0009	NA	0	0	0.0000	0.0000
	10.04.2018	100	131511730	3.167E+10	0.2 -3 µm	2.00	3	753	0.0000	0.0006	NA	0	0	0.0000	0.0000
	12.04.2018	102	137437816	3.31E+10	0.2 -3 µm	2.78	7	1508	0.0000	0.0012	1.11	2	455	0.0000	0.0004
	17.04.2018	108	134007214	3.216E+10	0.2 -3 µm	3.86	6	1471	0.0000	0.0012	2.59	4	973	0.0000	0.0008
	19.04.2018	110	145428602	3.516E+10	0.2 -3 µm	NA	0	0	0.0000	0.0000		0	0	0.0000	0.0000
	24.04.2018	115	148226228	3.577E+10	0.2 -3 µm	2.47	4	939	0.0000	0.0007	NA	0	0	0.0000	0.0000
	26.04.2018	117	186004950	4.485E+10	0.2 -3 µm	9.61	28	6592	0.0000	0.0039	NA	0	0	0.0000	0.0000
	02.05.2018	123	172417734	4.171E+10	0.2 -3 µm	18.37	48	11750	0.0000	0.0075	2.36	5	1236	0.0000	0.0008
	03.05.2018	124	175408924	4.239E+10	0.2 -3 µm	5.68	13	2945	0.0000	0.0019	1.19	3	641	0.0000	0.0004
	08.05.2018	129	157697368	3.823E+10	0.2 -3 µm	6.67	14	3451	0.0000	0.0024	NA	0	0	0.0000	0.0000
	11.05.2018	134	174367208	4.196E+10	0.2 -3 µm	2.17	4	955	0.0000	0.0006	0.54	1	204	0.0000	0.0001
	15.05.2018	136	167144802	4.036E+10	0.2 -3 µm	2.90	5	1199	0.0000	0.0008	NA	0	0	0.0000	0.0000
	17.05.2018	142	178481766	4.291E+10	0.2 -3 µm	1.99	3	749	0.0000	0.0005	NA	0	0	0.0000	0.0000
	22.05.2018	143	162504646	3.904E+10	0.2 -3 µm	6.50	14	3489	0.0000	0.0024	1.33	3	750	0.0000	0.0005
	24.05.2018	145	159197066	3.831E+10	0.2 -3 µm	0.60	2	445	0.0000	0.0003	NA	0	0	0.0000	0.0000
	29.05.2018	150	171271466	4.143E+10	0.2 -3 µm	4.70	12	2757	0.0000	0.0018	NA	0	0	0.0000	0.0000

19.03.2018	78	408373164	9.731E+10	3-10 µm		14.71	61	14854	0.0000	0.0041		1.34	5	1252	0.0000	0.0003
12.04.2018	102	332059730	7.927E+10	3-10 µm		7.97	23	5501	0.0000	0.0018	NA		0	0	0.0000	0.0000
17.04.2018	108	420871688	9.615E+10	3-10 µm		9.07	36	9030	0.0000	0.0025		1.20	4	1004	0.0000	0.0003
26.04.2018	117	341246096	8.148E+10	3-10 µm		17.39	54	12787	0.0000	0.0042		1.58	4	1002	0.0000	0.0003
08.05.2018	129	390032052	8.751E+10	3-10 µm		2.67	6	1506	0.0000	0.0005	NA		0	0	0.0000	0.0000
11.05.2018	134	346400002	8.256E+10	3-10 µm		1.26	4	856	0.0000	0.0003	NA		0	0	0.0000	0.0000
22.05.2018	143	322898796	7.6E+10	3-10 µm		1.33	8	2003	0.0000	0.0007	NA		0	0	0.0000	0.0000
29.05.2018	150	361105792	8.634E+10	3-10 µm		8.94	19	4492	0.0000	0.0014	NA		0	0	0.0000	0.0000
19.03.2018	78	229237742	5.467E+10	>10 µm	NA		0	0	0.0000	0.0000			0	0	0.0000	0.0000
12.04.2018	102	366243118	8.751E+10	>10 µm		31.54	12562	2964733	0.0034	0.9023		9.73	47	11267	0.0000	0.0034
17.04.2018	108	349480760	8.224E+10	>10 µm		1.81	10	2361	0.0000	0.0008	NA		0	0	0.0000	0.0000
26.04.2018	117	357085094	8.282E+10	>10 µm		28.24	123	28151	0.0000	0.0091		3.04	9	1940	0.0000	0.0006
08.05.2018	129	332293300	7.447E+10	>10 µm		10.89	30	6873	0.0000	0.0025		1.50	3	748	0.0000	0.0003
11.05.2018	134	340848252	8.026E+10	>10 µm		1.06	2	501	0.0000	0.0002	NA		0	0	0.0000	0.0000
22.05.2018	143	416648526	9.496E+10	>10 µm		61.98	301	70233	0.0001	0.0197		33.33	110	26002	0.0000	0.0073
29.05.2018	150	369290526	7.388E+10	>10 µm		5.65	42	7238	0.0000	0.0026	NA		0	0	0.0000	0.0000

Table 17: Isolation and cultivation specifics of eight strains obtained from two particle fractions at Helgoland Roads (54°11'03"N, 7°54'00"E) during mid-March and mid-May 2017.

Genus	Strain	DSMZ accession number	Sampling date	Medium
Polaribacter	AHE13PA	DSM111061	15.03.2017	Laminarin
Tenacibaculum	AHE14PA	DSM111040	15.03.2017	Laminarin
Tenacibaculum	AHE15PA	DSM111039	15.03.2017	Laminarin
Winogradskyella	AHE16PA		15.05.2017	2216
Mesonia	AHE17PA		15.03.2017	2216
Marixanthomonas	AHE18PA		15.03.2017	Laminarin
Arenibacter	AHE19PA		15.03.2017	2216
Polaribacter	AHE20PA		15.05.2017	Laminarin

References

1. Bennke CM, Reintjes G, Schattenhofer M, Ellrott A, Wulf J, Zeder M, et al. Modification of a high-throughput automatic microbial cell enumeration system for shipboard analyses. Applied and Environmental Microbiology. 2016;82(11):3289-96.
2. Manz W, Amann R, Ludwig W, Vancanneyt M, Schleifer K-H. Application of a suite of 16S rRNA-specific oligonucleotide probes designed to investigate bacteria of the phylum *Cytophaga-Flavobacter-Bacteroides* in the natural environment. Microbiology. 1996;142(5):1097-106.
3. Pernthaler J, Zöllner E, Warnecke F, Jürgens K. Bloom of filamentous bacteria in a mesotrophic lake: identity and potential controlling mechanism. Applied and Environmental Microbiology. 2004;70(10):6272-81.
4. Noble RT, Fuhrman JA. Use of SYBR Green I for rapid epifluorescence counts of marine viruses and bacteria. Aquatic Microbial Ecology. 1998;14(2):113-8.
5. Patel A, Noble RT, Steele JA, Schwalbach MS, Hewson I, Fuhrman JA. Virus and prokaryote enumeration from planktonic aquatic environments by epifluorescence microscopy with SYBR Green I. Nature Protocols. 2007;2(2):269-76.
6. Brum JR, Steward GF, Jiang SC, Jellison R. Spatial and temporal variability of prokaryotes, viruses, and viral infections of prokaryotes in an alkaline, hypersaline lake. Aquatic Microbial Ecology. 2005;41(3):247-60.
7. Zobell CE. Studies on marine bacteria. I. The cultural requirements of heterotrophic aerobes. Journal of Marine Research. 1941;4:41-75.
8. Moebus K. A method for the detection of bacteriophages from ocean water. Helgoländer Meeresuntersuchungen. 1980;34(1):1-14.
9. Yamamoto KR, Alberts BM, Benzinger R, Lawhorne L, Treiber G. Rapid bacteriophage sedimentation in the presence of polyethylene glycol and its application to large-scale virus purification. Virology. 1970;40(3):734-44.
10. Sullivan MB, Huang KH, Ignacio-Espinoza JC, Berlin AM, Kelly L, Weigele PR, et al. Genomic analysis of oceanic cyanobacterial myoviruses compared with T4-like myoviruses from diverse hosts and environments. Environmental Microbiology. 2010;12(11):3035-56.
11. Bankevich A, Nurk S, Antipov D, Gurevich AA, Dvorkin M, Kulikov AS, et al. SPAdes: a new genome assembly algorithm and its applications to single-cell sequencing. Journal of Computational Biology. 2012;19(5):455-77.
12. Wick RR, Schultz MB, Zobel J, Holt KE. Bandage: interactive visualization of de novo genome assemblies. Bioinformatics. 2015;31(20):3350-2.
13. Santos F, Meyerdierks A, Peña A, Rosselló-Mora R, Amann R, Antón J. Metagenomic approach to the study of halophages: the environmental halophage 1. Environmental Microbiology. 2007;9(7):1711-23.
14. Noguchi H, Taniguchi T, Itoh T. MetaGeneAnnotator: detecting species-specific patterns of ribosomal binding site for precise gene prediction in anonymous prokaryotic and phage genomes. DNA Research. 2008;15(6):387-96.
15. Camacho C, Coulouris G, Avagyan V, Ma N, Papadopoulos J, Bealer K, et al. BLAST+: architecture and applications. BMC Bioinformatics. 2009;10(1):421.
16. Mizuno CM, Ghai R, Saghaï A, López-García P, Rodriguez-Valera F. Genomes of abundant and widespread viruses from the deep ocean. mBio. 2016;7(4):e00805-16.

17. Mizuno CM, Rodriguez-Valera F, Kimes NE, Ghai R. Expanding the marine virosphere using metagenomics. PLOS Genetics. 2013;9(12):e1003987.
18. Nishimura Y, Watai H, Honda T, Mihara T, Omae K, Roux S, et al. Environmental viral genomes shed new light on virus-host interactions in the ocean. mSphere. 2017;2(2):e00359-16.
19. Paez-Espino D, Roux S, Chen IMA, Palaniappan K, Ratner A, Chu K, et al. IMG/VR v.2.0: an integrated data management and analysis system for cultivated and environmental viral genomes. Nucleic Acids Research. 2019;47(D1):D678-D86.
20. Gregory AC, Zayed AA, Conceição-Neto N, Temperton B, Bolduc B, Alberti A, et al. Marine DNA viral macro- and microdiversity from pole to pole. Cell. 2019;177(5):1109-23.e14.
21. Labonté JM, Swan BK, Poulos B, Luo H, Koren S, Hallam SJ, et al. Single-cell genomics-based analysis of virus–host interactions in marine surface bacterioplankton. The ISME Journal. 2015;9(11):2386-99.
22. Martinez-Hernandez F, Fornas O, Lluesma Gomez M, Bolduc B, de la Cruz Peña MJ, Martínez JM, et al. Single-virus genomics reveals hidden cosmopolitan and abundant viruses. Nature Communications. 2017;8(1):15892.
23. Jang HB, Bolduc B, Zablocki O, Kuhn JH, Roux S, Adriaenssens EM, et al. Taxonomic assignment of uncultivated prokaryotic virus genomes is enabled by gene-sharing networks. Nature Biotechnology. 2019;37(6):632-9.
24. Bolduc B, Jang HB, Doulcier G, You Z-Q, Roux S, Sullivan MB. vConTACT: an iVirus tool to classify double-stranded DNA viruses that infect *Archaea* and *Bacteria*. PeerJ. 2017;5:e3243.
25. Chan PP, Lowe TM. tRNAscan-SE: searching for tRNA genes in genomic sequences. Methods in Molecular Biology. 2019;1962:1-14.
26. Lowe TM, Eddy SR. tRNAscan-SE: a program for improved detection of transfer RNA genes in genomic sequence. Nucleic Acids Research. 1997;25(5):955-64.
27. Laslett D, Canback B. ARAGORN, a program to detect tRNA genes and tmRNA genes in nucleotide sequences. Nucleic Acids Research. 2004;32(1):11-6.
28. Jones P, Binns D, Chang H-Y, Fraser M, Li W, McAnulla C, et al. InterProScan 5: genome-scale protein function classification. Bioinformatics. 2014;30(9):1236-40.
29. Kearse M, Moir R, Wilson A, Stones-Havas S, Cheung M, Sturrock S, et al. Geneious Basic: an integrated and extendable desktop software platform for the organization and analysis of sequence data. Bioinformatics. 2012;28(12):1647-9.
30. Hahnke RL, Harder J. Phylogenetic diversity of *Flavobacteria* isolated from the North Sea on solid media. Systematic and Applied Microbiology. 2013;36(7):497-504.
31. Muyzer G, Teske A, Wirsen CO, Jannasch HW. Phylogenetic relationships of *Thiomicrospira* species and their identification in deep-sea hydrothermal vent samples by denaturing gradient gel electrophoresis of 16S rDNA fragments. Archives of Microbiology. 1995;164(3):165-72.
32. Chin C-S, Alexander DH, Marks P, Klammer AA, Drake J, Heiner C, et al. Nonhybrid, finished microbial genome assemblies from long-read SMRT sequencing data. Nature Methods. 2013;10(6):563-9.
33. Koren S, Walenz BP, Berlin K, Miller JR, Bergman NH, Phillippy AM. Canu: scalable and accurate long-read assembly via adaptive k-mer weighting and repeat separation. Genome Research. 2017.
34. Rodriguez-R LM, Gunturu S, Harvey WT, Rosselló-Mora R, Tiedje JM, Cole JR, et al. The Microbial Genomes Atlas (MiGA) webserver: taxonomic and gene diversity analysis of *Archaea* and *Bacteria* at the whole genome level. Nucleic Acids Research. 2018;46(W1):W282-W8.

35. Rodriguez-R LM, Konstantinidis KT. The enveomics collection: a toolbox for specialized analyses of microbial genomes and metagenomes. PeerJ Preprints. 2016;4:e1900v1.
36. Holmfeldt K, Middelboe M, Nybroe O, Riemann L. Large variabilities in host strain susceptibility and phage host range govern interactions between lytic marine phages and their *Flavobacterium* hosts. Applied and Environmental Microbiology. 2007;73(21):6730-9.
37. Holmfeldt K, Odić D, Sullivan MB, Middelboe M, Riemann L. Cultivated single-stranded DNA phages that infect marine *Bacteroidetes* prove difficult to detect with DNA-binding stains. Applied and Environmental Microbiology. 2012;78(3):892-4.
38. Roux S, Brum JR, Dutilh BE, Sunagawa S, Duhaime MB, Loy A, et al. Ecogenomics and potential biogeochemical impacts of globally abundant ocean viruses. Nature. 2016;537(7622):689-93.
39. Shimodaira H, Terada Y. Selective inference for testing trees and edges in phylogenetics. Frontiers in Ecology and Evolution. 2019;7:174.
40. Suzuki R, Shimodaira H. Pvclust: an R package for assessing the uncertainty in hierarchical clustering. Bioinformatics. 2006;22(12):1540-2.
41. Panschin I, Becher M, Verbarg S, Spröer C, Rohde M, Schüler M, et al. Description of *Gramella forsetii* sp. nov., a marine *Flavobacteriaceae* isolated from North Sea water, and emended description of *Gramella gaetbulicola* Cho et al. 2011. International Journal of Systematic and Evolutionary Microbiology. 2017;67(3):697-703.
42. Alejandre-Colomo C, Harder J, Fuchs BM, Rosselló-Móra R, Amann R. High-throughput cultivation of heterotrophic bacteria during a spring phytoplankton bloom in the North Sea. Systematic and Applied Microbiology. 2020;43(2):126066.
43. Barbeyron T, Carpentier F, L'haridon S, Schüler M, Michel G, Amann R. Description of *Maribacter forsetii* sp. nov., a marine *Flavobacteriaceae* isolated from North Sea water, and emended description of the genus *Maribacter*. International Journal of Systematic and Evolutionary Microbiology. 2008;58(4):790-7.
