# Supplementary figures and images for: Highly diverse flavobacterial phages isolated from North Sea spring blooms
Source: ISME J. 2021 Sep 2;16(2):555–68. doi: 10.1038/s41396-021-01097-4 (PMC8776804; doi:10.1038/s41396-021-01097-4)

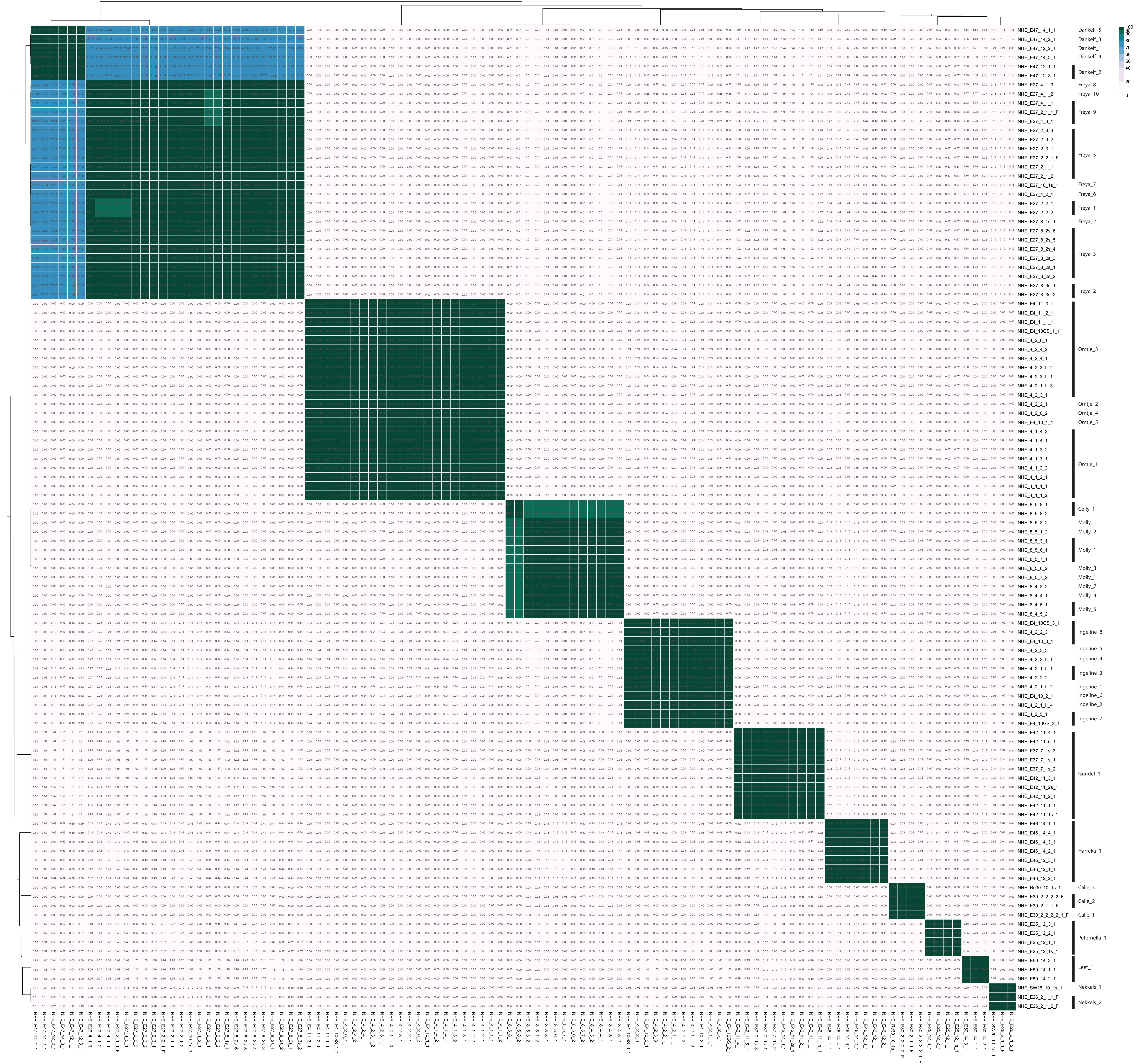

Supplement: Supplementary file 2 — SI_File_2 [file 41396_2021_1097_MOESM2_ESM.pdf]

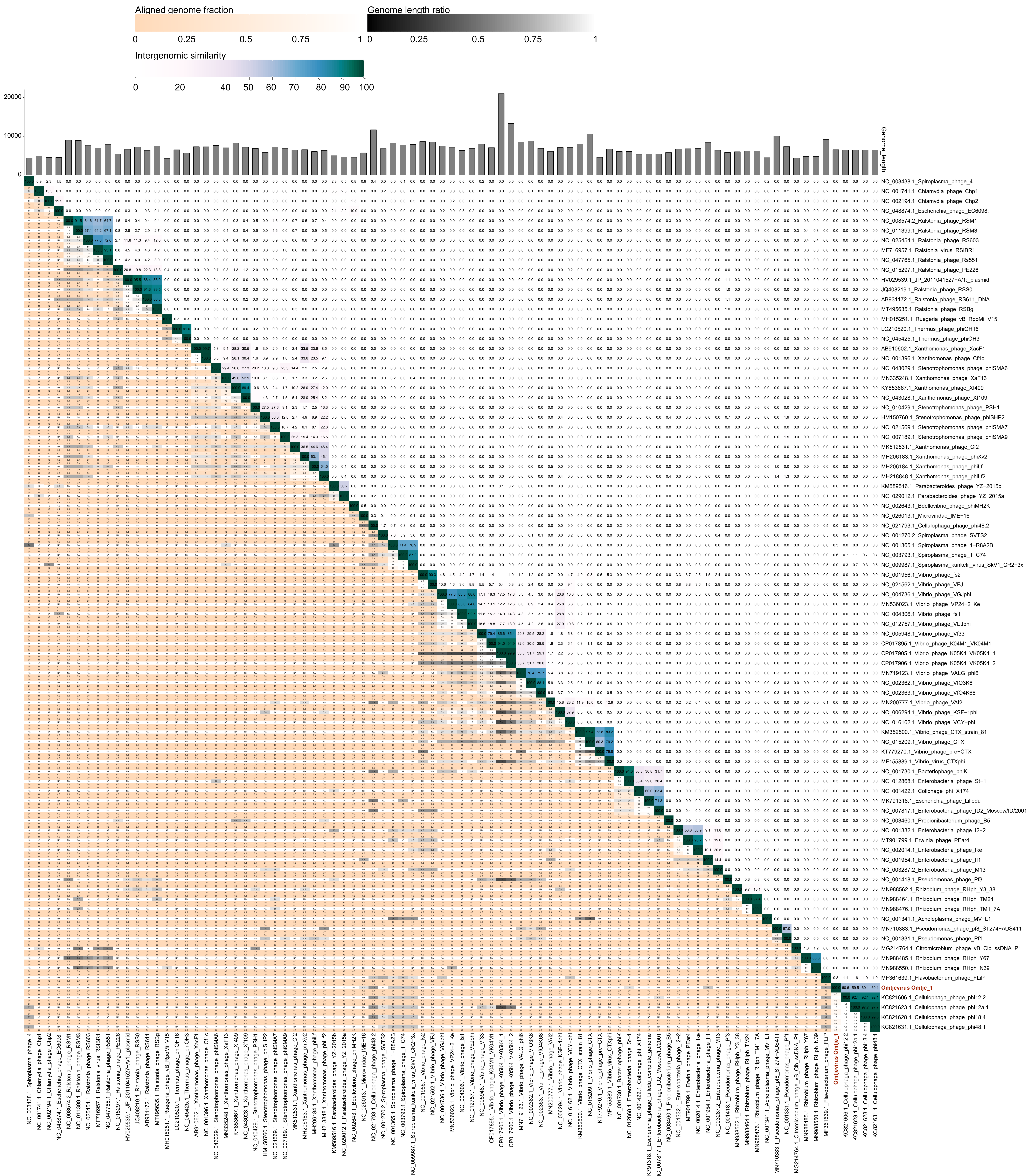

Supplement: Supplementary file 6 — SI_File_6 [file 41396_2021_1097_MOESM6_ESM.pdf]
