# Supplementary material for: Highly diverse flavobacterial phages isolated from North Sea spring blooms
Source: ISME J. 2021 Sep 2;16(2):555–68. doi: 10.1038/s41396-021-01097-4 (PMC8776804; doi:10.1038/s41396-021-01097-4)
Supplement: Supplementary file 5 — SI_File_5 [file 41396_2021_1097_MOESM5_ESM.pdf]

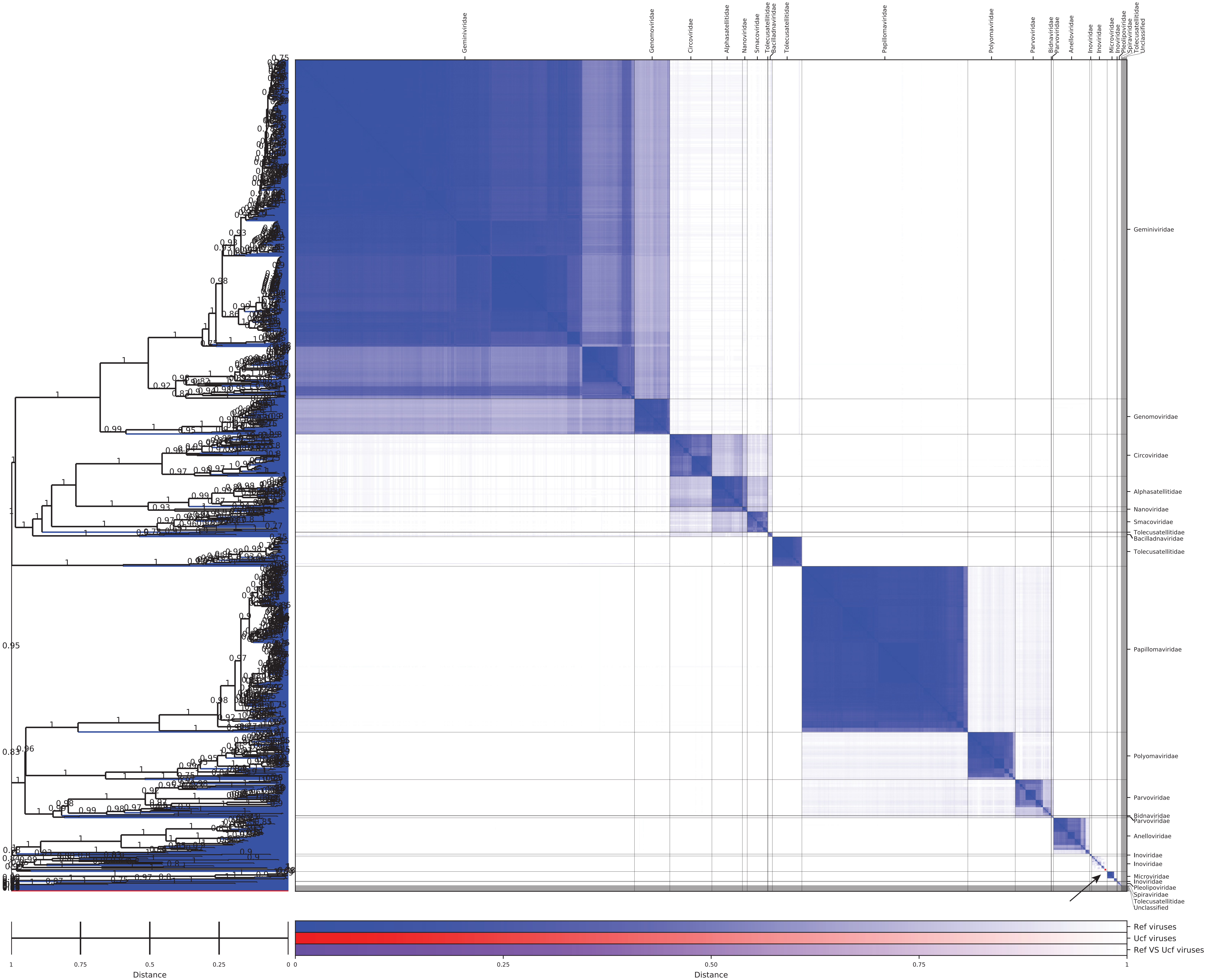

SI file 5: Heatmap from GRAViTy with new ssDNA phage isolate, ssDNA Cellulophaga phages and the Baltimore Group II - ssDNA viruses + *Papillomaviridae* and *Polyomaviridae* (VMRv34) database. Red squares indicate the position in the heat map of the
