# Supplementary material for: Highly diverse flavobacterial phages isolated from North Sea spring blooms
Source: ISME J. 2021 Sep 2;16(2):555–68. doi: 10.1038/s41396-021-01097-4 (PMC8776804; doi:10.1038/s41396-021-01097-4)
Supplement: Supplementary file 3 — SI_File_3 [file 41396_2021_1097_MOESM3_ESM.pdf]

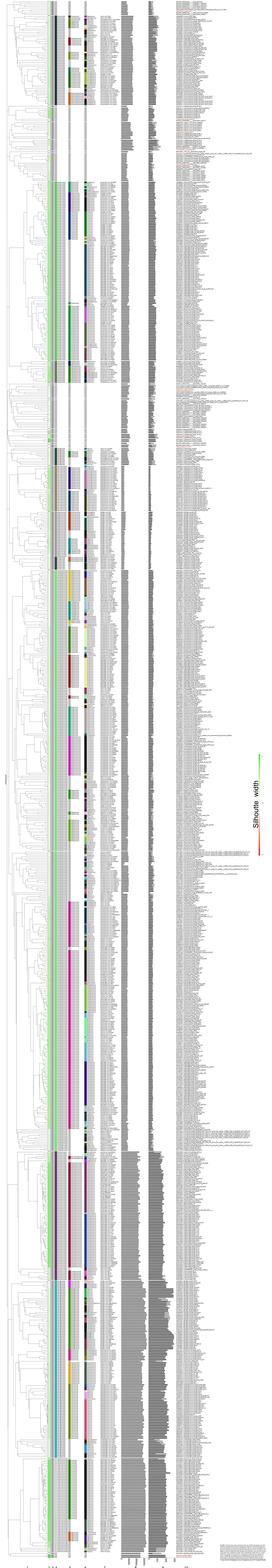

SI. File 3: VirClust hierarchical clustering of the new dsDNA flavophages and their relatives, extended dataset, based on intergenomic distances calculated using the protein cluster content. **1.** Hierarchical clustering tree. The tree was cut into smaller viral genome clusters (VGCs) using an 0.9 distance threshold. Each VGC is framed in a rectangle in 2 and 3. **2.** Silhouette width, measures how related is a virus with other viruses in the same VGCs. Similarity to other VGCs is indicated by values closer to -1 (red). Similarity to viruses in the same VGC is indicated by values closer to 1 (green). **3**. VGCs - numbering. **4.** Family (ICTV). **5.** Subfamily (ICTV). **6.** Genus (ICTV). **7.** Species (ICTV). **8.** Genome length (bps). **9.** Fraction of proteins shared with other viruses (dark grey), based on protein assignment to PCs. **10.** Virus genome names. **To note: the new flavophages are not yet assigned to any ICTV recognized family.**
