# Supplementary material for: Highly diverse flavobacterial phages isolated from North Sea spring blooms
Source: ISME J. 2021 Sep 2;16(2):555–68. doi: 10.1038/s41396-021-01097-4 (PMC8776804; doi:10.1038/s41396-021-01097-4)
Supplement: Supplementary file 4 — SI_File_4 [file 41396_2021_1097_MOESM4_ESM.pdf]

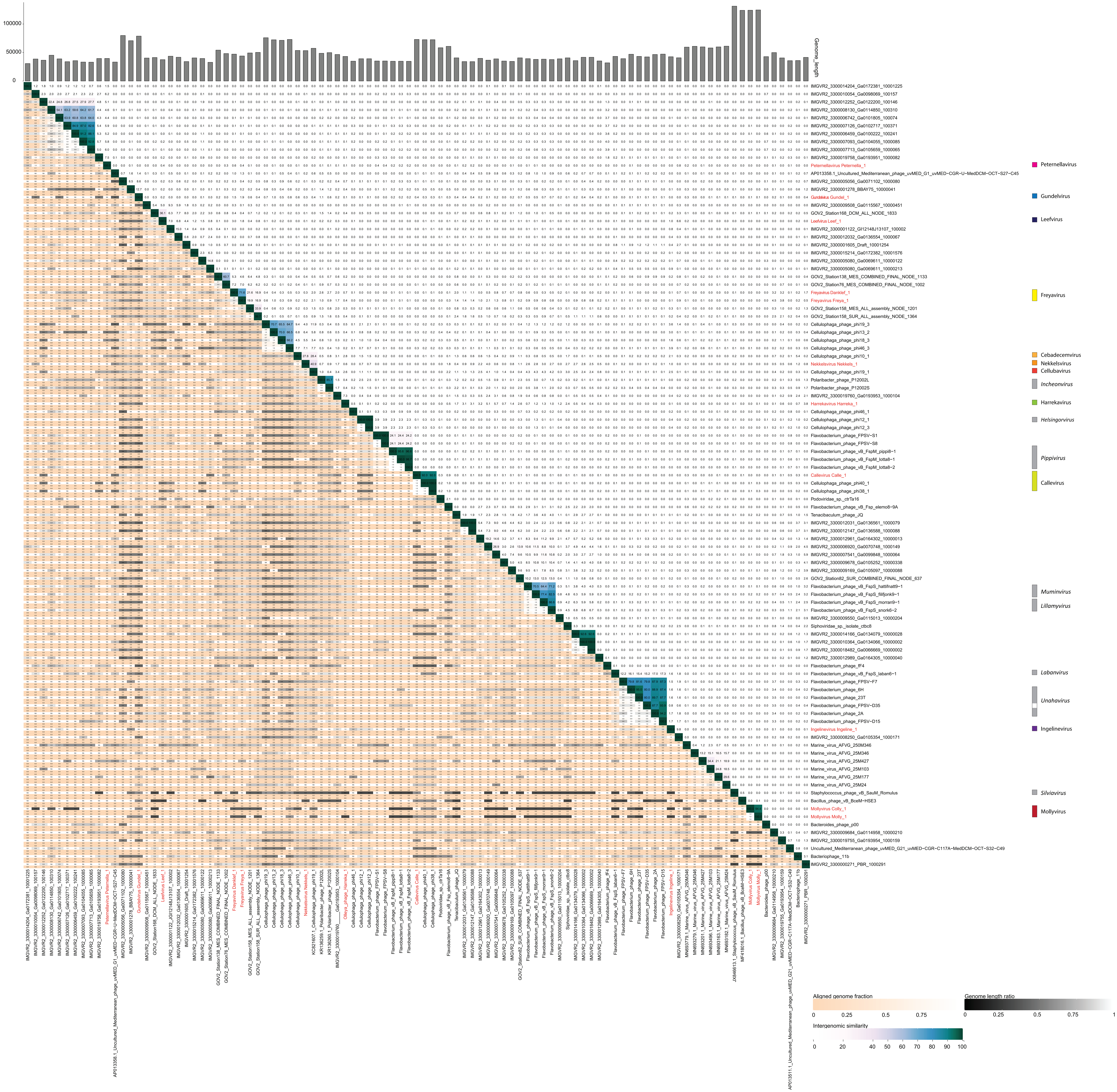

SI **file** 4: Intergenomic nucleotide similarity matrix of new isolates, environmental genomes and reference genomes. Phage genera are indicated on the right side. Phage genera in grey indicate official ICTV accepted genera.
